# Amine–borane complex-initiated SF_5_Cl radical addition on alkenes and alkynes

**DOI:** 10.3762/bjoc.16.256

**Published:** 2020-12-16

**Authors:** Audrey Gilbert, Pauline Langowski, Marine Delgado, Laurent Chabaud, Mathieu Pucheault, Jean-François Paquin

**Affiliations:** 1 Départment de chimie, Université Laval, Québec, QC, G1V 0A6, Canada; 2 Institut des Sciences Moléculaires - Groupe ORGA - UMR 5255, Université de Bordeaux, 351 Cours de la libération, 33405 Talence, France

**Keywords:** amine-borane complex, pentafluorosulfanyl chloride, pentafluorosulfanyl substituent, radical addition, radical initiation

## Abstract

The SF_5_Cl radical addition on unsaturated compounds was performed using an air-stable amine–borane complex as the radical initiator. This method showed to be complementary to the classic Et_3_B-mediated SF_5_Cl addition on alkenes and alkynes. A total of seven alkene and three alkyne derivatives were tested in the reaction, with yields ranging from 3% to 85%.

## Introduction

The pentafluorosulfanyl (SF_5_) substituent has been attracting its share of attention since its discovery in 1950 [1]. Often referred to as a "super CF_3_", the SF_5_ shows enhanced properties when compared to its trifluoromethylated (CF_3_) analog [2]. Indeed, the SF_5_ moiety is more electronegative, more lipophilic, bulkier, and more thermally and chemically stable than the CF_3_ substituent [3–8]. Furthermore, it induces a stronger dipole moment, which can dramatically affect the properties of the neighboring functional groups on a molecule [3–8]. Considering the wide range of trifluoromethylated compounds of interest, the synthesis of their SF_5_-analogs has become a trend to increase the properties of these valuable molecules [9]. Due to the unique properties, the SF_5_ group has been used in various fields of chemistry, including pharmaceuticals [10–16], agrochemistry [17–20], and materials sciences [21–26]. The applications have, however, been limited by the poor synthetic accessibility of SF_5_-containing molecules. As such, the development of alternative methods for the introduction of the SF_5_ group is highly relevant.

**Scheme 1:**
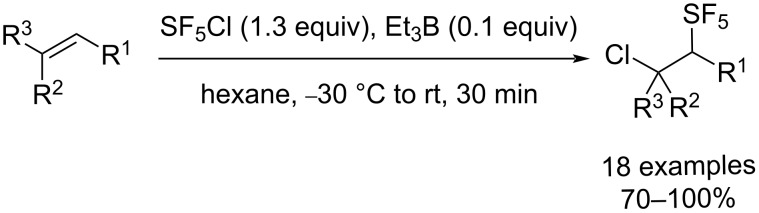
Dolbier’s protocol for the SF_5_Cl radical addition on alkenes.

Although the number of synthetic routes towards the SF_5_ substituent remains limited, a few methods have been developed in the past 20 years in order to include a SF_5_ moiety on various organic substrates [27–28]. The main strategy towards pentafluorosulfanylated aliphatic compounds, reported for the first time by Dolbier and co-workers in 2002, is the Et_3_B-mediated radical addition of SF_5_Cl on alkenes and alkynes (Scheme 1) [29–30]. This strategy represented a tremendous step forward in the pentafluorosulfanyl aliphatic chemistry, since it addressed the drawbacks that were previously reported with the use of SF_5_Cl as a reagent. It allows the SF_5_Cl addition to occur in the liquid phase (SF_5_Cl being a gas that boils at −21 °C) in milder reaction conditions and in normal glassware, instead of using special apparatus such as autoclaves and photochemical reactors [31]. Moreover, this method leads to significantly higher yields in shorter reaction times compared to the previous methods. Since this first report, this reaction has been extensively used to obtain a wide range of SF_5_-containing aliphatic derivatives, and represents the most versatile route towards pentafluorosulfanylated aliphatic compounds [28].

However, some limitations have emerged from the Dolbier protocol. The SF_5_Cl addition on unsaturated compounds goes through a free-radical mechanism, and is promoted by the radical activation of SF_5_Cl by Et_3_B, which leads to the formation of the propagating species SF_5_^•^ [32–33]. Trialkylboranes are common low-temperature radical initiators, and Et_3_B is one of the most used in the literature [34–35]. The use of the reagent allows the radicals to form, even at very low temperature, due to its strong reactivity with oxygen. However, the disadvantage for the use of the reagent comes from the same property: Et_3_B is an oxygen-sensitive and pyrophoric compound even at low temperatures, which requires the use of air-free techniques in the laboratory. Et_3_B in the pure form has limited commercial availability and is known to spontaneously react with oxygen to produce a green flame [36]. To avoid the pyrophoric properties of triethylborane, this reagent is mostly sold in low-concentrated solutions (typically 1–2 M in hexane, THF or Et_2_O). Another concern is the fluctuation in the quality/concentration in commercial solutions, even among the same batch from the same supplier. Finally, in some cases, the reaction yields can be poorly reproducible if the trialkylborane reagent is not freshly prepared [37]. It is therefore of interest to address this challenge in order to widen the scope of this transformation.

Compared to trialkylboranes, amine–borane complexes have shown to be more stable [38]. Indeed, they are usually air-stable, and their preparation from NaBH_4_, H_2_SO_4_ and amines involves traditional mild acidic work-up without degradation. They are therefore much easier to handle on a laboratory scale and can be stored on the shelf for months without noticeable alteration of their properties and purity [39]. Amine–borane complexes have been extensively used in the literature as hydrogen reservoirs [40], as reducing agents in various transformations, including the reduction of aldehydes, amides and ketones, reductive aminations, alkene hydroboration, and carbon bond forming reaction [41–42], as well as various boronate and borinic acid precursors [43–47]. More recently, it has been shown that some of these common amine–borane complexes can also be used as radical initiators for atom transfer radical addition of alkyl halides to alkenes [48]. They were also used in the free-radical polymerization of alkene-containing monomers such as methyl methacrylate or styrene [48–50]. We envisioned that it could be possible to replace the Et_3_B in Dolbier’s protocol by a stable amine–borane complex that could perform the radical initiation of SF_5_Cl on its addition on alkenes. This would address the drawbacks associated with the use of Et_3_B as the radical initiator, and therefore facilitate the access to various pentafluorosulfanylated derivatives.

**Scheme 2:**
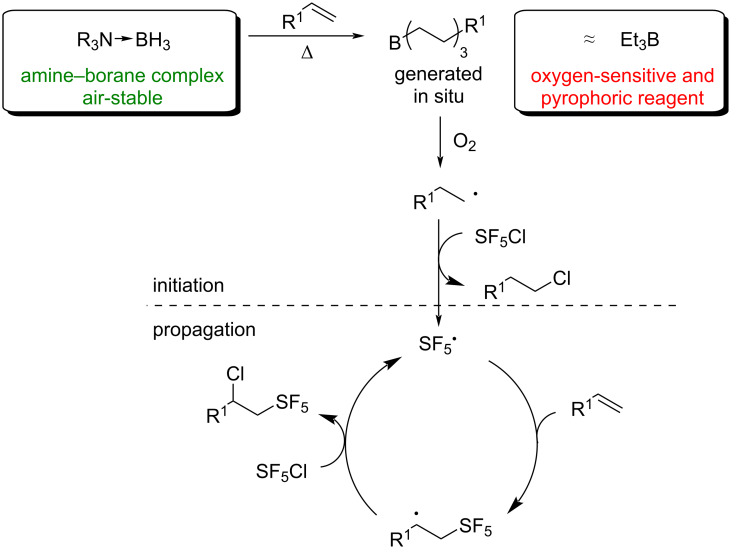
Proposed mechanism for the amine–borane complex-initiated radical addition of SF_5_Cl on alkenes.

As shown in Scheme 2, we envisioned that the amine–borane complex-initiated reaction of SF_5_Cl with alkenes would proceed following a mechanism similar to the amine–borane complex-initiated carbohalogenation of alkenes [48]. The first step would involve the formation of a trialkylborane species via the hydroboration of the alkene, as previously observed by ^11^B NMR spectroscopy [48–49]. In the presence of oxygen, the trialkylborane would, similarly to Et_3_B, generate an alkyl radical. The latter would react with SF_5_Cl to produce a chloroalkane as well as the key SF_5_^•^ radical. The propagation steps would occur exactly as reported by Dolbier and co-workers for the Et_3_B-mediated radical addition of SF_5_Cl on alkenes and alkynes [29–30]. Overall, while similar mechanistically, the use of an amine–borane complex as the initiator would avoid the need to manipulate an oxygen-sensitive and pyrophoric reagent.

## Results and Discussion

**Figure 1:**
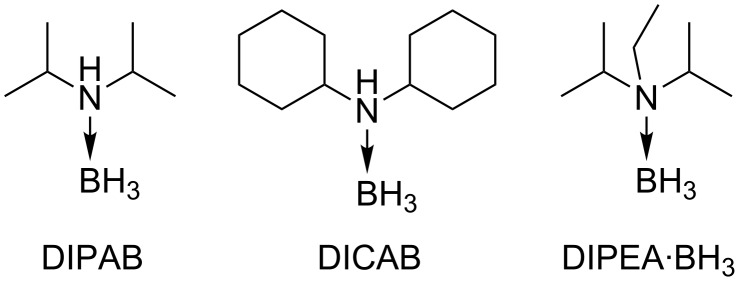
Structures and acronyms of the amine-borane complexes investigated.

Table 1 and Table 2 show the selected optimization results for the use of amine–borane complexes in the SF_5_Cl radical addition on alkenes. We chose allyl benzyl ether (**1**) as the model substrate for our optimization, since it has been previously shown that this compound undergoes SF_5_Cl radical addition following Dolbier’s protocol in various solvents with high yields [51]. We started the optimization with 3 equivalents of SF_5_Cl, 10 mol % of the amine–borane complex, with temperatures going from 30 °C to 60 °C for 3 hours. The addition of all reagents was performed at −40 °C, before the reaction vessel was hermetically sealed and heated to avoid evaporation of SF_5_Cl, since it is gaseous above −21 °C. The reaction was performed in common organic solvents that remain liquid both at −40 °C and at the tested reaction temperatures. The results in hexane, ethyl acetate (EtOAc), and methyl *tert*-butyl ether (MTBE) are shown in Table 1 and Table 2, but more solvents were tested and did not lead to higher yields of the desired compound (see Supporting Information File 1 for the complete optimization results). Three commercially available borane complexes were tested in the reaction, i.e., diisopropylamine borane (DIPAB), dicyclohexylamine borane (DICAB), and *N,N*-diisopropylethylamine borane (DIPEA·BH_3_) (Figure 1). The use of DIPAB and DICAB led to higher yields, and the results are respectively shown in Table 1 and Table 2 (see Supporting Information File 1 for the results with DIPEA·BH_3_).

We started the optimization with DIPAB as the amine–borane complex. To our delight, in all tested solvents and temperatures, we observed the formation of the desired product **2a**, which is in accordance with our hypothesis that the amine–borane complexes can indeed be used as radical initiators under thermal activation in the SF_5_Cl addition on alkenes. DIPAB showed to be generally more productive at lower temperatures. Indeed, when performing the reaction in EtOAc, a full conversion was observed at all tested temperatures, but with a decrease in the yield when the reaction temperature was increased (Table 1, entries 5–8). The same effect was observed in MTBE, with the best results obtained at 30 °C and 50 °C, while 60 °C led to a low yield of 26% (Table 1, entries 9–12). Surprisingly, the intermediate temperature of 40 °C led to only 6% of the desired compound, and the reason for this result remains unclear (Table 1, entry 10). Moreover, the use of hexane as the solvent did not show to be compatible with DIPAB as the amine–borane complex, since it led to low yields at all tested temperatures (Table 1, entries 1–4). When using DIPAB as the radical initiator, the best result was obtained in EtOAc at 30 °C, with a yield of 72% of the desired addition product (Table 1, entry 5).

**Table 1:** Selected optimization results for the SF_5_Cl addition on allyl benzyl ether (**1**) using DIPAB as the radical initiator.

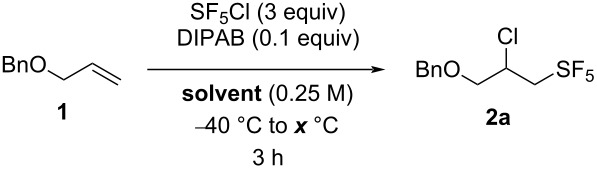				

entry	solvent	*x* (°C)	conversion (%)^a^	yield (%)^b^

1	hexane	30	28	2
2	hexane	40	32	6
3	hexane	50	33	7
4	hexane	60	55	5
5	EtOAc	30	100	72
6	EtOAc	40	100	64
7	EtOAc	50	100	5
8	EtOAc	60	100	29
9	MTBE	30	77	40
10	MTBE	40	69	6
11	MTBE	50	79	41
12	MTBE	60	92	26

^a^Disappearance of the starting material, estimated by ^1^H NMR analysis of the crude mixture using 2-fluoro-4-nitrotoluene as an internal standard. ^b^Yield estimated by ^19^F NMR analysis of the crude mixture using 2-fluoro-4-nitrotoluene as an internal standard.

We next turned our attention to the amine–borane complex DICAB as the radical initiator for the addition of SF_5_Cl on allyl benzyl ether (**1**) (Table 2). In this case, increasing the reaction temperature generally led to higher yields. When the reaction was performed in hexane, the yields went from 1–2% to 72% when heating the reaction at 50 °C, compared to 30 °C and 40 °C, while a low yield of 32% was obtained with the reaction temperature of 60 °C (Table 2, entries 1–4). This tendency was also observed when using EtOAc as the solvent (Table 2, entries 5–8), with the best yield of 62% obtained at 50 °C (Table 2, entry 7). We hypothesized that a higher temperature is necessary to activate the crystalline and more sterically hindered DICAB, compared to the liquid DIPAB, but that a too high reaction temperature such as 60 °C tends to increase the degradation pathways instead of the formation of the desired compound. However, this effect was not observed when performing the reaction in MTBE. A high yield of 86% was obtained at 60 °C (Table 2, entry 12), while performing the reaction at 40 °C led to a higher yield than at 50 °C (Table 2, entries 10 and 11). With the use of DICAB, the best result obtained was with MTBE as the solvent at 60 °C, affording the product with a yield of 86% (Table 2, entry 12).

**Table 2:** Selected optimization results for the SF_5_Cl addition on allyl benzyl ether (**1**) using DICAB as the radical initiator.

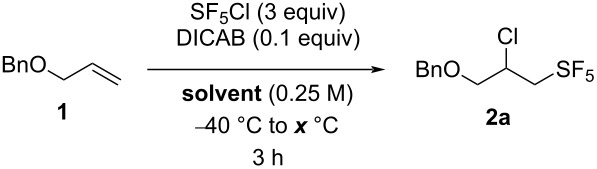				

entry	solvent	*x* (°C)	conversion (%)^a^	yield (%)^b^

1	hexane	30	25	1
2	hexane	40	17	2
3	hexane	50	82	72
4	hexane	60	76	32
5	EtOAc	30	100	traces
6	EtOAc	40	100	4
7	EtOAc	50	100	62
8	EtOAc	60	100	56
9	MTBE	30	49	3
10	MTBE	40	100	65
11	MTBE	50	94	21
12	MTBE	60	100	86 (77)^c^

^a^Disappearance of the starting material, estimated by ^1^H NMR analysis of the crude mixture using 2-fluoro-4-nitrotoluene as an internal standard. ^b^Yield estimated by ^19^F NMR analysis of the crude mixture using 2-fluoro-4-nitrotoluene as an internal standard. ^c^Isolated yield.

At this point, we performed some control reactions in order to get more insight into the reaction (Table 3). We first increased the amount of the amine–borane complex added to the reaction mixture to evaluate if this would promote the desired reaction. Hexane and EtOAc were tested at 50 °C with 20 mol % instead of 10 mol % of DICAB (Table 3, entries 1 and 2). This showed to be slightly beneficial in the case of EtOAc (Table 3, entry 1), while it led to a low yield in the case of hexane (Table 3, entry 2). Moreover, increasing the reaction time from 3 to 6 hours led to higher yields of the desired SF_5_-containing adduct, with yields of 80% and 89% with EtOAc and hexane, respectively (Table 3, entries 3 and 4). Finally, we performed the reaction in hexane with no amine–borane complex added, and only trace amounts of the final compound **2a** were detected (Table 3, entry 5).

**Table 3:** Effect of time and amount of the amine–borane complex on the SF_5_Cl addition on allyl benzyl ether (**1**).

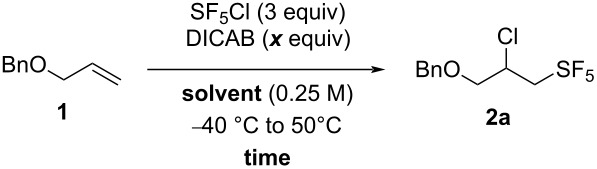					

entry	solvent	DICAB (equiv)	time (h)	conversion (%)^a^	yield (%)^b^

1	EtOAc	0.2	3	100	80
2	hexane	0.2	3	61	37
3	EtOAc	0.1	6	100	86
4	hexane	0.1	6	94	89
5	hexane	0	6	20	traces

^a^Disappearance of the starting material, estimated by ^1^H NMR analysis of the crude mixture using 2-fluoro-4-nitrotoluene as an internal standard. ^b^Yield estimated by ^19^F NMR analysis of the crude mixture using 2-fluoro-4-nitrotoluene as an internal standard.

We next wondered if it could be possible to start the reaction at a higher temperature than −40 °C without significantly affecting the reaction yield. We performed the reaction with 10 mol % of DICAB, using our standard conditions, but with the addition of all reagents at 0 °C or at room temperature (20–21 °C) before sealing the reaction vessel and heating the reaction mixture to 50 °C (Table 4). EtOAc, hexane, and MTBE were tested in these conditions, and with the exception of hexane when starting the reaction at 0 °C (Table 4, entry 3), all reactions led to the desired final compound in good to excellent yields (Table 4, entries 1, 2, and 4–6). Indeed, we obtained a 93% yield of the addition product **2a** when performing the reaction in EtOAc from 0 °C to 50 °C (Table 4, entry 1). The low yield of 2% that was obtained with hexane (Table 4, entry 3) is, however, rather surprising, since the reaction in that solvent from room temperature to 50 °C led to 81% of the desired compound (Table 4, entry 4). When repeating the reaction, we rapidly realized that these higher initial temperatures conditions were not reproducible, which we believe came from the fact that at 0 °C and room temperature, SF_5_Cl is gaseous. Therefore, its addition to the reaction mixture might have been inefficient in some case, while getting added properly in some other cases, which would explain the reproducibility problems. Moreover, this effect was not observed when repeating some of the reactions where the initial temperature was −40 °C, which is in accordance with this hypothesis.

**Table 4:** Effect of the initial temperature on the SF_5_Cl addition on allyl benzyl ether (**1**).

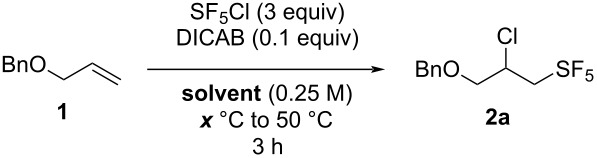				

entry	solvent	*x* (°C)	conversion (%)^a^	yield (%)^b^

1	EtOAc	0	100	93
2	EtOAc	20	100	71
3	hexane	0	40	2
4	hexane	20	100	81
5	MTBE	0	100	75
6	MTBE	20	100	79

^a^Disappearance of the starting material, estimated by ^1^H NMR analysis of the crude mixture using 2-fluoro-4-nitrotoluene as an internal standard. ^b^Yield estimated by ^19^F NMR analysis of the crude mixture using 2-fluoro-4-nitrotoluene as an internal standard.

Finally, we hypothesized that reducing the amount of the amine–borane complex in the reaction could increase the yield by avoiding a surge of the radical species early in the reaction. We performed the reactions with 3.3 mol % of the amine–borane complex in hexane, EtOAc, and MTBE and at 50 °C and 60 °C (Table 5). Unfortunately, the reaction yields did not increase, and these reaction conditions proved to be less efficient for the SF_5_Cl addition on allyl benzyl ether than the ones previously discussed. The only case where the yield was promoted by a decreased amount of the amine–borane complex was when the reaction was performed in hexane at 50 °C and with the use of DICAB as the radical initiator (Table 5, entry 2). Indeed, this led to a product yield of 86%, which is equal to the best yield obtained so far, in MTBE with 10 mol % of DICAB at 60 °C (Table 2, entry 12). However, the latter led to a full conversion, which is not the case with the reaction in hexane, with a conversion of 93%. Considering the similar polarity of the starting material **1** and the final product **2a**, we chose the reaction conditions in MTBE as the optimal conditions, in order to facilitate the purification process.

**Table 5:** Effect of the decreased amount of the amine-borane complex on the SF_5_Cl addition on allyl benzyl ether (**1**).

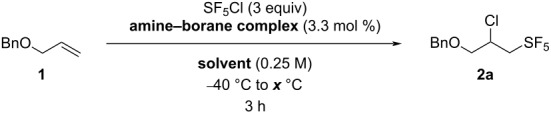					

entry	solvent	amine–borane complex	*x* (°C)	conversion (%)^a^	yield (%)^b^

1	hexane	DIPAB	50	25	2
2	hexane	DICAB	50	93	86
3	hexane	DIPAB	60	65	41
4	hexane	DICAB	60	30	22
5	EtOAc	DIPAB	50	54	9
6	EtOAc	DICAB	50	66	2
7	EtOAc	DIPAB	60	100	6
8	EtOAc	DICAB	60	100	37
9	MTBE	DIPAB	50	67	20
10	MTBE	DICAB	50	100	73
11	MTBE	DIPAB	60	67	24
12	MTBE	DICAB	60	100	75

^a^Disappearance of the starting material, estimated by ^1^H NMR analysis of the crude mixture using 2-fluoro-4-nitrotoluene as an internal standard. ^b^Yield estimated by ^19^F NMR analysis of the crude mixture using 2-fluoro-4-nitrotoluene as an internal standard.

**Scheme 3:**
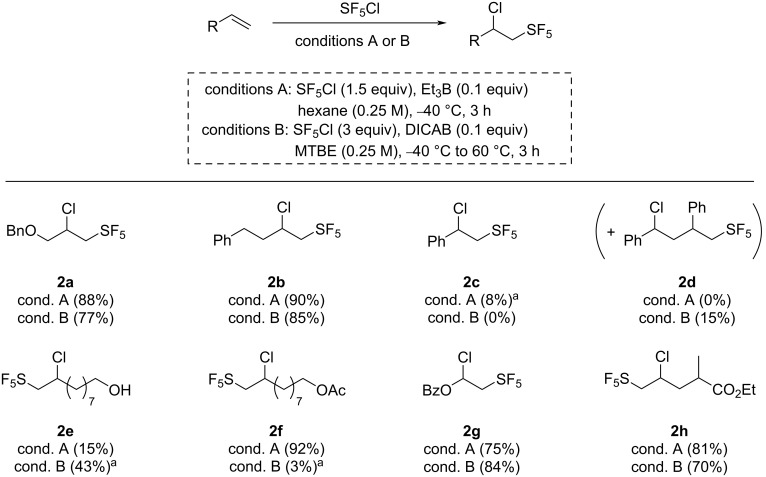
Scope of the Et_3_B and the DICAB-initiated SF_5_Cl additions on alkenes. Unless noted otherwise, isolated yields are reported. ^a^Yield estimated by ^19^F NMR analysis of the crude mixture using 2-fluoro-4-nitrotoluene as an internal standard.

With the optimal reaction conditions in hand, we evaluated the scope of the reaction. Both our DICAB-promoted and the Dolbier’s protocol using Et_3_B for the SF_5_Cl radical addition on unsaturated compounds were performed on every substrate, in order to compare the two methods. First, a series of alkenes was assessed for the SF_5_Cl radical addition using both protocols (Scheme 3). In most cases, the desired pentafluorosulfanylated compounds were obtained in comparable yields with both methods, with a slightly better yield for the Dolbier protocol. Indeed, compound **2a** was obtained with an 88% yield when the SF_5_Cl addition was performed with Et_3_B, while 77% of the desired final product was obtained with the DICAB-mediated protocol. Moreover, yields of 90% and 85% of compound **2b** were respectively obtained when using 4-phenyl-1-butene as the starting material. When performing the reactions on styrene, the desired addition product **2c** was only observed with a low NMR yield of 8% with the Et_3_B-mediated reaction, while the DICAB protocol led to a 15% isolated yield of the 2:1 addition product **2d**, with no sign of the desired compound **2c**. Furthermore, as expected, a low yield of 15% of the corresponding addition product **2e** was obtained with Dolbier’s protocol when performing the reaction on dec-9-en-1-ol, while protecting the alcohol group with an acetate significantly increased the yield, leading to the corresponding pentafluorosulfanylated derivative **2f** with a 92% yield. However, this effect was not observed when the DICAB protocol was performed on these two substrates. In both cases, while the final compounds could not be isolated from the reaction mixture, comparison of the NMR yields of both crude mixtures showed that the alcohol was more tolerated in the reaction with DICAB, albeit the final compound **2e** was obtained with the moderate NMR yield of 43%. The SF_5_Cl addition on the acetate derivative led to only 3% NMR yield of the final compound **2f** and the reaction mixture showed the presence of various degradation compounds. Finally, the SF_5_Cl addition was performed on two ester derivatives. When using vinyl benzoate as the starting material, the desired compound **2g** was obtained with a 75% yield with Dolbier’s protocol and an 84% yield with the DICAB protocol, while the addition product **2h** was obtained with an 81% yield using Et_3_B as the radical initiator and 70% when the reaction was performed with DICAB.

**Scheme 4:**
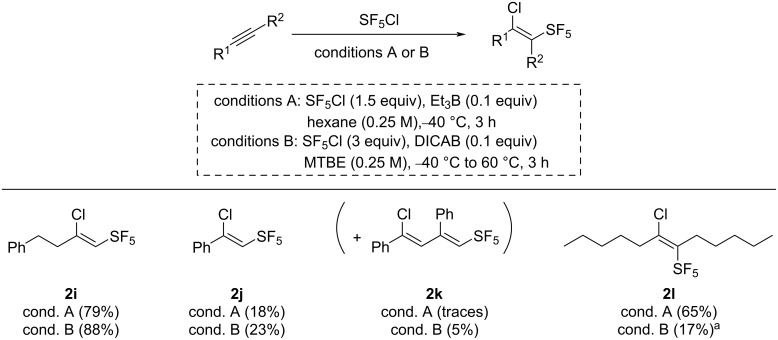
Scope of the Et_3_B and the DICAB-initiated SF_5_Cl additions on alkynes. Unless noted otherwise, isolated yields are reported. ^a^Yield estimated by ^19^F NMR analysis of the crude mixture using 2-fluoro-4-nitrotoluene as an internal standard.

Next, the SF_5_Cl radical additions on alkynes using both protocols were performed (Scheme 4). First, 4-phenyl-1-butyne was evaluated, and the desired pentafluorosulfanylated product **2i** was obtained with a 79% yield with the Et_3_B-mediated reaction, and a higher yield of 88% when using DICAB as the radical initiator. In the case of the SF_5_Cl addition on phenylacetylene, it has been reported in the initial report from Dolbier that the formation of the side-product **2k**, resulting from the 2:1 addition of the starting material on the intermediate radical, occurred with the Et_3_B-mediated reaction [29]. In our hands, Dolbier’s protocol led to only trace amounts of the compound **2k** and 18% of the desired addition product **2j**, while the reaction with the DICAB conditions led to 23% of the desired compound **2j**, and 5% of the 2:1 addition side product **2k**. Finally, the reaction was performed on the internal alkyne 6-dodecyne, and the Dolbier’s protocol led to the moderate yield of 65% of the desired compound **2l**, while only a 17% NMR yield was obtained in the reaction using DICAB as the radical initiator.

## Conclusion

In conclusion, we have shown that amine–borane complexes can be used as radical initiators under thermal conditions to perform the SF_5_Cl radical addition on unsaturated compounds. These air-stable complexes can therefore be used as alternatives to the more unstable and pyrophoric Et_3_B, in order to incorporate the SF_5_ substituent on aliphatic derivatives. A total of 7 examples of alkene derivatives and 3 examples of alkyne derivatives were evaluated in the reaction, with yields ranging from 3% to 85%. Overall, this reaction represents a complementary method to the Et_3_B-mediated SF_5_Cl addition on unsaturated compounds.

## Supporting Information

File 1 General information, synthetic procedures, additional optimization results, NMR spectra for known compounds (^1^H, ^19^F) and full characterization of all new compounds.
