# Correction: Amine–borane complex-initiated SF_5_Cl radical addition on alkenes and alkynes

**DOI:** 10.3762/bjoc.17.120

**Published:** 2021-07-23

**Authors:** Audrey Gilbert, Pauline Langowski, Marine Delgado, Laurent Chabaud, Mathieu Pucheault, Jean-François Paquin

**Affiliations:** 1 Départment de chimie, Université Laval, Québec, QC, G1V 0A6, Canada; 2 Institut des Sciences Moléculaires - Groupe ORGA - UMR 5255, Université de Bordeaux, 351 Cours de la libération, 33405 Talence, France

**Keywords:** amine–borane complex, pentafluorosulfanyl chloride, pentafluorosulfanyl substituent, radical addition, radical initiation

The stereochemistry of some alkene products (**2i–k**) in Scheme 4 of the original publication was misattributed. The corrected structures are shown in Scheme 1.

**Scheme 1:**
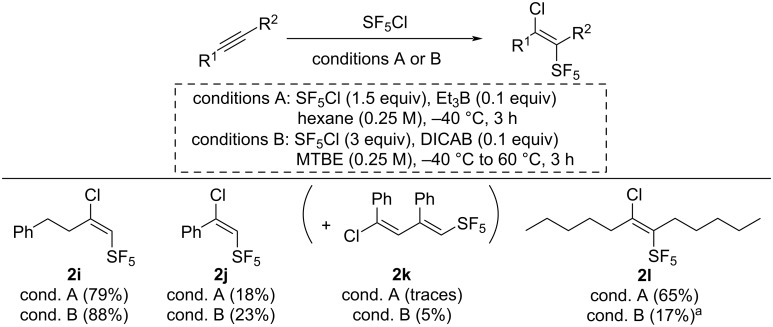
Corrected Scheme 4 of the original article. Scope of the Et_3_B and the DICAB-initiated SF_5_Cl additions on alkynes. Unless noted otherwise, isolated yields are reported. ^a^Yield estimated by ^19^F NMR analysis of the crude mixture using 2-fluoro-4-nitrotoluene as an internal standard.

A corrected version of Supporting Information File 1 is also part of this Correction. The new Supporting Information File 1 is the complete file with the corrections marked in yellow color.

Finally, the Table of Content graphic was also corrected. The corrected version of the original graphical abstract is shown in Scheme 2.

**Scheme 2:**
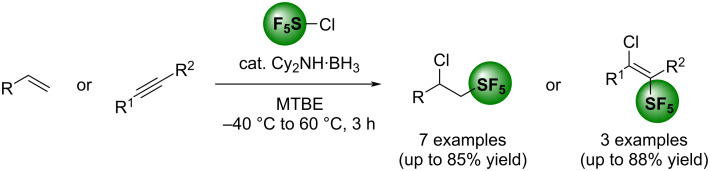
Corrected graphical abstract of the original publication.

We apologize for any inconvenience caused.

## Supporting Information

File 1 General information, synthetic procedures, additional optimization results, NMR spectra for known compounds (^1^H, ^19^F) and full characterization of all new compounds.

