# Supplementary material for: Amine–borane complex-initiated SF5Cl radical addition on alkenes and alkynes
Source: Beilstein J Org Chem. 2020 Dec 16;16:3069–77. doi: 10.3762/bjoc.16.256 (PMC7753108; doi:10.3762/bjoc.16.256)
Supplement: File 1 — General information, synthetic procedures, additional optimization results, NMR spectra for known compounds (1H, 19F) and full characterization of all new compounds. [file Beilstein_J_Org_Chem-16-3069-s001.pdf]

BEILSTEIN JOURNAL OF ORGANIC CHEMISTRY

# Supporting Information

for

# Amine–borane complex-initiated $SF_5Cl$ radical addition on alkenes and alkynes

Audrey Gilbert, Pauline Langowski, Marine Delgado, Laurent Chabaud, Mathieu Pucheault and Jean-François Paquin

*Beilstein J. Org. Chem.* **2020,** *16,* 3069–3077. doi:10.3762/bjoc.16.256

# General information, synthetic procedures, additional optimization results, NMR spectra for known compounds ($^1H$, $^{19}F$) and full characterization of all new compounds

License and Terms: This is a supporting information file under the terms of the Creative Commons Attribution License (https://creativecommons.org/licenses/by/4.0). Please note that the reuse, redistribution and reproduction in particular requires that the author(s) and source are credited and that individual graphics may be subject to special legal provisions.
The license is subject to the *Beilstein Journal of Organic Chemistry* terms and conditions: (https://www.beilstein-journals.org/bjoc/terms)

## Table of contents

General information ............................................................ S2

Synthesis of dec-9-en-1-yl acetate ............................................................ S3

General procedures for the $SF_5Cl$ additions ............................................................ S3

$SF_5Cl$ additions reactions ............................................................ S4

Full optimization results ............................................................ S13

NMR spectra ............................................................ S17

## General information

All reactions were carried out under an argon atmosphere. $Et_2O$, dichloromethane, THF, and toluene were purified using a Vacuum Atmospheres Inc. Solvent Purification System. All other commercially available compounds were used as received. Thin-layer chromatography (TLC) analyses of reaction mixtures were performed using Silicycle silica gel 60 Å F254 TLC plates and visualized under UV light or by staining with potassium permanganate. Flash column chromatography was carried out on Silicycle silica gel 60 Å, 230–400 mesh. $^1$H, $^{13}$C and $^{19}$F spectra were respectively recorded at 500, 126, and 470 MHz using $CDCl_3$ as the solvent at ambient temperature on an Agilent Technologies 500/54 Premium Shielded spectrometer. The internal standard used was: for $^1$H NMR tetramethylsilane ($\delta$ = 0 ppm), for $^{13}$C NMR tetramethylsilane ($\delta$ = 0 ppm). For $^{19}$F spectra, calibration was performed using a unified scale [1]. Coupling constants (*J*) are measured in hertz (Hz). Multiplicities are reported using the following abbreviations: s = singlet, d = doublet, t = triplet, q = quartet, p = quintet, h = sextet, M = multiplet, br = broad resonance. Low-resolution mass spectra were obtained on a GC–MS using chemical ionization (CI). Infrared spectra were recorded using an ABB MB300 FT-IR spectrometer.

[1] Harris, R. K.; Becker, E. D.; Cabral de Menezes, S. M.; Goodfellow, R.; Granger, P. *Pure Appl. Chem.* **2001**, *73*, 1795–1818.

**Synthesis of dec-9-en-1-yl acetate**

**Dec-9-en-1-yl acetate:** Following a procedure described by Schmalz et al. [2], dec-9-en-1-ol (1 equiv, 200 mg, 1.28 mmol) and pyridine (10 mL, 0.13 M) were charged in a round-bottomed flask at 0 °C. The solution was degassed with argon before a solution of acetic anhydride in pyridine (0.25 M, 1.2 equiv, 1.54 mmol) was added dropwise. The mixture was allowed to warm to room temperature and stirred overnight. The reaction was quenched by the addition of water, before pyridine was evaporated under reduced pressure. The crude mixture was then dissolved in $Et_2O$ and successively washed with a 10% HCl solution in water and brine. The organic phase was dried over $MgSO_4$, filtered, and evaporated under reduce pressure. The crude product was purified by flash chromatography on silica gel using hexane/EtOAc 90:10 as the eluent to yield the title compound as colorless oil (71%, 179.3 mg). $^1H$ NMR (500 MHz, $CDCl_3$): δ (ppm) = 5.82 (ddt, *J* = 16.9, 10.2, 6.7 Hz, 1H), 5.00 (dq, *J* = 17.1, 1.7 Hz, 1H), 4.94 (ddt, *J* = 10.2, 2.3, 1.2 Hz, 1H), 4.06 (t, *J* = 6.8 Hz, 2H), 2.08 – 2.01 (m, 5H), 1.67 – 1.56 (m, 2H), 1.45 – 1.20 (m, 10H). Analytical data were identical to those previously reported [3].

**General procedures for the $SF_5Cl$ additions**

**Method A:** A microwave vial under inert atmosphere was successively charged with the unsaturated compound (1.0 equiv) and degassed hexanes (0.25 M) at −40 °C. A solution of $SF_5Cl$ in hexanes (1.5 equiv) was added, followed by a

[2] Hirschhäuser, C.; Velcicky, J.; Schlawe, D.; Hessler, E.; Majdalani, A.; Neudörfl, J.-M.; Prokop, A.; Wieder, T.; Schmalz, H.-G. *Chem. Eur. J.* **2013**, *19*, 13017-13029.
[3] Winter, R.; Nixon, P. G.; Gard, G. L.; Radford, D. H.; Holcomb, N. R.; Grainger, D. W. *J. Fluorine Chem.* **2001**, *107*, 23-30.

solution of $Et_3B$ (1 M in THF, 0.1 equiv). The mixture was stirred for 3 h at −40 °C. The reaction mixture was then allowed to warm to room temperature, and a saturated solution of $NaHCO_3$ was added to quench the reaction. The phases were separated and the organic phase was dried over $MgSO_4$. The crude product was concentrated under reduced pressure and purified by flash chromatography on silica gel.

**Method B:** A microwave vial under inert atmosphere was successively charged with the unsaturated compound (1.0 equiv) and degassed MTBE (0.33 M) at −40 °C. The vial was hermetically sealed, before a solution of $SF_5Cl$ in hexanes (3 equiv) was added. A solution of DICAB (0.10 M in MTBE, 0.1 equiv) was then added and the mixture was stirred for 3 h at 60 °C. The reaction mixture was then allowed to cool to room temperature and a saturated solution of $NaHCO_3$ was added to quench the reaction. The phases were separated and the organic phase was dried over $MgSO_4$. The crude product was concentrated under reduced pressure and purified by flash chromatography on silica gel.

**$SF_5Cl$ additions reactions**

**(2-Chloro-2-phenethoxyethyl)pentafluoro-$\lambda^6$-sulfane (2a):**

**Method A:** ((Allyloxy)methyl)benzene (**1**, 100 mg, 0.67 mmol), $SF_5Cl$ (1.37 M in hexanes, 0.74 mL, 1.01 mmol), and $Et_3B$ (1 M in THF, 67 µL, 0.067 mmol) were engaged in general procedure, Method A, to afford the title compound **2a** as colorless oil (185.5 mg, 0.60 mmol, 88%) after purification by flash chromatography using hexanes/EtOAc 95:5 as the eluent. **Method B:**

((Allyloxy)methyl)benzene (**1**, 100 mg, 0.67 mmol), $SF_5Cl$ (1.32 M in hexanes, 1.53 mL, 2.02 mmol), and DICAB (13.17 mg, 0.067 mmol) were engaged in general procedure, Method B, to afford the title compound **2a** as colorless oil (161.0 mg, 0.52 mmol, 77%) after purification by flash chromatography using hexanes/EtOAc 95:5 as the eluent. $^1$H NMR (500 MHz, $CDCl_3$): δ (ppm) = 7.41 – 7.34 (m, 2H), 7.36 – 7.30 (m, 3H), 4.59 (s, 2H), 4.51 – 4.43 (m, 1H), 4.34 – 4.20 (m, 1H), 3.95 – 3.80 (m, 1H), 3.76 (dd, *J* = 10.4, 4.6 Hz, 1H), 3.60 (dd, *J* = 10.4, 6.7 Hz, 1H) ; $^{19}$F NMR (470 MHz, $CDCl_3$): δ (ppm) = 83.9 – 82.4 (m, 1F), 66.6 (dt, *J* = 146.7, 8.0 Hz, 4F). Analytical data were identical to those previously reported [4].

**(2-Chloro-4-phenylbutyl)pentafluoro-λ$^6$-sulfane (2b): Method A:** 4-Phenyl-1-butene (100 mg, 0.76 mmol), $SF_5Cl$ (1.32 M in hexanes, 0.86 mL, 1.13 mmol), and $Et_3B$ (1 M in THF, 76 µL, 0.076 mmol) were engaged in general procedure, Method A, to afford the title compound **2b** as colorless oil (201.4 mg, 0.68 mmol, 90%) after purification by flash chromatography using 100% hexanes as the eluent. **Method B:** 4-Phenyl-1-butene (100 mg, 0.67 mmol), $SF_5Cl$ (1.37 M in hexanes, 1.66 mL, 2.27 mmol), and DICAB (14.75 mg, 0.076 mmol) were engaged in general procedure, Method B, to afford the title compound **2b** as colorless oil (191.2 mg, 0.65 mmol, 85%) after purification by flash chromatography using 100% hexanes as the eluent. $^1$H NMR (500 MHz, $CDCl_3$): δ (ppm) = 7.32 (t, *J* = 7.3 Hz, 2H), 7.27 – 7.17 (m, 3H), 4.34 – 4.26 (m, 1H), 4.03 (td, *J* = 14.4, 8.4 Hz, 1H), 3.91 (td, *J* =

[4] Gilbert, A.; Paquin, J.-F. *J. Fluorine Chem.* **2019**, *221*, 70-74.

14.3, 7.9 Hz, 1H), 2.98 – 2.89 (m, 1H), 2.83 – 2.74 (m, 1H), 2.32 – 2.21 (m, 1H), 2.04 (td, $J$ = 14.2, 9.3 Hz, 1H); $^{19}$F NMR (470 MHz, $CDCl_3$): δ (ppm) = 83.9 – 82.4 (m, 1F), 66.5 (dt, $J$ = 146.6, 8.2 Hz, 4F). Analytical data were identical to those previously reported [5].

**(2-Chloro-2-phenylethyl)pentafluoro-λ$^6$-sulfane (2c): Method A:** Styrene (100 mg, 0.38 mmol), $SF_5Cl$ (1.32 M in hexanes, 0.43 mL, 0.56 mmol), and $Et_3B$ (1 M in THF, 38 µL, 0.038 mmol) were engaged in general procedure, Method A, to afford the title compound **2c** which could not be isolated. The yield was estimated using NMR analysis and 2-fluoro-4-nitrotoluene as the reference; yield: 8%.

**(4-Chloro-2,4-diphenylbutyl)pentafluoro-λ$^6$-sulfane (2d): Method B:** Styrene (200 mg, 1.92 mmol), $SF_5Cl$ (1.37 M in hexanes, 4.21 mL, 5.76 mmol), and DICAB (37.5 mg, 0.19 mmol) were engaged in general procedure, Method B, to afford the title compound **2d** as colorless oil (113.0 mg, 0.30 mmol, 15%) after purification by flash chromatography using 100% hexane as the eluent. $^1$H NMR (500 MHz, $CDCl_3$): δ (ppm) = 7.44 – 7.39 (m, 10H), 5.39 (t, $J$ = 6.8 Hz, 1H), 5.03 (dd, $J$ = 7.9, 6.6 Hz, 1H), 4.37 – 4.27 (m, 2H), 4.02 (dd, $J$ = 11.3, 6.6 Hz, 1H), 3.95 (dd, $J$ = 11.4, 7.9 Hz, 1H); $^{13}$C NMR (126 MHz, $CDCl_3$): δ (ppm) = 138.5, 138.0, 129.4, 129.2, 129.1, 128.8, 127.4, 126.9, 76.9 (p, $J$ = 13.3 Hz), 61.8, 56.4 (p, $J$ = 4.7 Hz), 48.4; $^{19}$F NMR (470 MHz, $CDCl_3$): δ (ppm) = 83.2 – 80.8 (m, 1F), 66.4 (dt, $J$ = 147.1,

[5] Ponomarenko, M. V.; Serguchev, Y. A.; Röschenthaler, G.-V. *Synthesis* **2010**, *22*, 3906-3912.

7.8 Hz, 4F); GC-MS (CI): m/z calcd for $C_{16}H_{15}ClF_5S$ $[M-H]^+$ 369.05 found 369.07 (under all conditions tested for high-resolution mass spectra [ESI(+), ESI(−), APPI], no significant ion was detected); IR (ATR, Diamond): ν ($cm^{-1}$) = 3032, 1495, 1456, 1310, 1198, 878, 824, 696.

**9-Chloro-10-(pentafluoro-$\lambda^6$-sulfanyl)decan-1-ol (2e): Method A:** Dec-9-en-1-ol (50 mg, 0.32 mmol), $SF_5Cl$ (0.96 M in hexanes, 0.50 mL, 0.48 mmol), and $Et_3B$ (1 M in THF, 32 µL, 0.032 mmol) were engaged in general procedure, Method A, to afford the title compound **2e** as colorless oil (15.1 mg, 0.047 mmol, 15%) after purification by flash chromatography using hexanes/EtOAc 95:5 as the eluent. **Method B:** Dec-9-en-1-ol (50 mg, 0.32 mmol), $SF_5Cl$ (0.96 M in hexanes, 0.99 mL, 0.96 mmol), and DICAB (6.2 mg, 0.032 mmol) were engaged in general procedure, Method B, to afford the title compound **2e** which could not be isolated. The yield was estimated using NMR analysis and 2-fluoro-4-nitrotoluene as the reference; yield: 43%. $^1$H NMR (500 MHz, $CDCl_3$): δ (ppm) = 4.40 – 4.30 (m, 1H), 4.06 – 3.96 (m, 1H), 3.95 – 3.85 (m, 1H), 3.40 (t, *J* = 6.7 Hz, 2H), 1.97 – 1.87 (m, 1H), 1.82 – 1.67 (m, 1H), 1.67 – 1.25 (m, 13H); $^{19}$F NMR (470 MHz, $CDCl_3$): δ (ppm) = 88.17 – 78.75 (m), 66.19 (dt, *J* = 146.5, 8.3 Hz). Analytical data were identical to those previously reported [3, 6].

[6] Dolbier Jr., W. R.; Aït-Mohand, S.; Schertz, T. D.; Sergeeva, T. A.; Cradlebaugh, J. A.; Mitani, A.; Gard, G. L.; Winter, R. W.; Thrasher, J. S. *J. Fluorine Chem.* **2006**, *127*, 1302-1310.

**9-Chloro-10-(pentafluoro- λ$^6$-sulfanyl)decyl acetate (2f): Method A:** Dec-9-en-1-yl acetate (50 mg, 0.25 mmol), $SF_5Cl$ (0.96 M in hexanes, 0.39 mL, 0.38 mmol), and $Et_3B$ (1 M in THF, 25 μL, 0.032 mmol) were engaged in general procedure, Method A, to afford the title compound **2f** as colorless oil (84.0 mg, 0.23 mmol, 92%) after purification by flash chromatography using hexanes/EtOAc 95:5 as the eluent. **Method B:** Dec-9-en-1-yl acetate (33.7 mg, 0.17 mmol), $SF_5Cl$ (0.96 M in hexanes, 0.53 mL, 0.51 mmol), and DICAB (3.3 mg, 0.017 mmol) were engaged in general procedure, Method B, to afford the title compound **2f** which could not be isolated. The yield was estimated using NMR analysis and 2-fluoro-4-nitrotoluene as the reference; yield: 3%. $^1$H NMR (500 MHz, $CDCl_3$): δ (ppm) = 4.39 – 4.27 (m, 1H), 4.05 (t, *J* = 6.7 Hz, 2H), 4.03 – 3.96 (m, 1H), 3.95 – 3.85 (m, 1H), 2.05 (s, 3H), 1.98 – 1.84 (m, 1H), 1.80 – 1.68 (m, 1H), 1.65 – 1.53 (m, 3H), 1.50 – 1.42 (m, 1H), 1.38 – 1.28 (m, 8H); $^{19}$F NMR (470 MHz, $CDCl_3$): δ (ppm) = 84.29 – 81.36 (m, 1F), 66.16 (dt, *J* = 146.3, 8.2 Hz, 4F). Analytical data were identical to those previously reported [3, 6].

**1-Chloro-2-(pentafluoro-λ$^6$-sulfanyl)ethyl benzoate (2g): Method A:** Vinyl benzoate (100 mg, 0.68 mmol), $SF_5Cl$ (1.32 M in hexanes, 0.77 mL, 1.01 mmol), and $Et_3B$ (1 M in THF, 68 μL, 0.068 mmol) were engaged in general procedure, Method A, to afford the title compound **2g** as colorless oil (156.7 mg, 0.50 mmol, 75%) after purification by flash chromatography using hexanes/$Et_2O$ 98:2 as the eluent. **Method B:** Vinyl benzoate (100 mg, 0.68 mmol), $SF_5Cl$ (1.32 M in hexanes, 1.53 mL, 2.03 mmol), and DICAB (13.17 mg, 0.068 mmol) were

engaged in general procedure, Method B, to afford the title compound **2g** as colorless oil (175.5 mg, 0.57 mmol, 84%) after purification by flash chromatography using hexanes/$Et_2O$ 98:2 as the eluent. $^1$H NMR (500 MHz, $CDCl_3$): δ (ppm) = 8.10 – 8.05 (m, 2H), 7.69 – 7.62 (m, 1H), 7.53 – 7.46 (m, 2H), 7.14 (dd, *J* = 9.8, 2.1 Hz, 1H), 4.49 – 4.37 (m, 1H), 4.26 – 4.16 (m, 1H); $^{13}$C NMR (126 MHz, $CDCl_3$): δ (ppm) = 163.4, 134.5, 130.2, 128.8, 127.9, 77.6 (p, *J* = 5.4 Hz), 73.4 (p, *J* = 14.7 Hz); $^{19}$F NMR (470 MHz, $CDCl_3$): δ (ppm) = 81.5 – 80.1 (m, 1F), 66.8 (dt, *J* = 147.2, 8.1 Hz, 4F); GC-MS (CI): m/z calcd for $C_9H_9ClF_5O_2S$ $[M+H]^+$ 310.99 found 311.00 (under all conditions tested for high-resolution mass spectra [ESI (+), ESI (–), APPI], no significant ion was detected); IR (ATR, Diamond): ν ($cm^{-1}$) = 3040, 1744, 1603, 1452, 1244, 1103, 1061, 837.

**Ethyl 4-chloro-2-methyl-5-(pentafluoro-λ$^6$-sulfanyl)pentanoate (2h): Method A:** Ethyl 2-methylpent-4-enoate (50 mg, 0.35 mmol), $SF_5Cl$ (0.96 M in hexanes, 0.55 mL, 0.52 mmol), and $Et_3B$ (1 M in THF, 35 µL, 0.035 mmol) were engaged in general procedure, Method A, to afford the title compound **2h** as colorless oil (86.8 mg, 0.28 mmol, 81%) in a 63:37 mixture of diastereoisomers after purification by flash chromatography using hexanes/EtOAc 90:10 as the eluent. **Method B:** Ethyl 2-methylpent-4-enoate (50 mg, 0.35 mmol), $SF_5Cl$ (0.96 M in hexanes, 1.10 mL, 1.05 mmol), and DICAB (6.9 mg, 0.035 mmol) were engaged in general procedure, Method B, to afford the title compound **2h** as colorless oil (74.7 mg, 0.25 mmol, 70%) in a 57:43 mixture of diastereoisomers after purification by flash chromatography using hexanes/EtOAc 95:5 as the eluent. $^1$H NMR (500 MHz,

$CDCl_3$): δ (ppm) = 4.50 – 4.44 (m, 1H), 4.42 – 4.35 (m, 0.6H), 4.17 (app. p, *J* = 7.1 Hz, 3.2H), 4.07 – 3.97 (m, 1.6H), 3.96 – 3.82 (m, 1.6H), 2.90 – 2.81 (m, 1H), 2.81 – 2.71 (m, 0.6H), 2.35 (ddd, *J* = 13.8, 10.7, 2.7 Hz, 1H), 2.18 (ddd, *J* = 15.4, 10.5, 5.1 Hz, 0.6H), 2.01 (ddd, *J* = 14.4, 8.9, 3.5 Hz, 0.6H), 1.68 (ddd, *J* = 14.3, 11.0, 3.2 Hz, 1H), 1.31 – 1.24 (m, 4.8H), 1.24 (d, *J* = 7.2 Hz, 3H), 1.22 (d, *J* = 7.0 Hz, 1.8H); $^{19}$F NMR (470 MHz, $CDCl_3$): δ (ppm) = 83.6 – 82.0 (m, 1.6F), 66.6 (dt, *J* = 146.6, 8.1 Hz, 4F), 66.4 (dt, *J* = 146.5, 8.3 Hz, 2.4F). Analytical data were identical to those previously reported [7].

**(*Z*)-(2-Chloro-3-phenylprop-1-en-1-yl)pentafluoro-λ$^6$-sulfane (2i): Method A:** 4-Phenyl-1-butyne (50 mg, 0.38 mmol), $SF_5Cl$ (0.96 M in hexanes, 0.60 mL, 0.58 mmol), and $Et_3B$ (1 M in THF, 38 µL, 0.038 mmol) were engaged in general procedure, Method A, to afford the title compound **2i** as colorless oil (89 mg, 0.30 mmol, 79%) after purification by flash chromatography using 100% hexanes as the eluent. **Method B:** 4-Phenyl-1-butyne (100 mg, 0.77 mmol), $SF_5Cl$ (1.37 M in hexanes, 1.68 mL, 2.30 mmol), and DICAB (14.99 mg, 0.077 mmol) were engaged in general procedure, Method B, to afford the title compound **2i** as colorless oil (196.8 mg, 0.67 mmol, 88%) after purification by flash chromatography using 100% hexanes as the eluent. $^1$H NMR (500 MHz, $CDCl_3$): δ (ppm) = 7.35 – 7.28 (m, 2H), 7.26 – 7.17 (m, 3H), 6.64 (p, *J* = 8.2 Hz, 1H), 3.00 – 2.89 (m, 4H); $^{13}$C NMR (126 MHz, $CDCl_3$): δ (ppm) = 146.4 (p, *J* = 6.4 Hz), 139.5, 137.3 (p, *J* = 21.8 Hz), 128.7, 128.5, 126.7, 38.3, 33.5; $^{19}$F NMR (470 MHz, $CDCl_3$):

[7] Lim, D. S.; Ngo, S. C.; Lal, S. G.; Minnich, K. E.; Welch, J. T. *Tetrahedron Lett.* **2008**, *49*, 5662-5663.

δ (ppm) = 83.4 – 82.0 (m, 1F), 67.3 (dd, *J* = 151.4, 8.3 Hz, 4F); GC-MS (CI): m/z calcd for $C_{10}H_9ClF_5S$ $[M-H]^+$ 291.00 found 291.00 (under all conditions tested for high-resolution mass spectra [ESI (+), ESI (–), APPI], no significant ion was detected); IR (ATR, Diamond): ν ($cm^{-1}$) = 3090, 3030, 1639, 1456, 1178, 1040, 989, 833.

**(*Z*)-(2-Chloro-2-phenylvinyl)pentafluoro-λ⁶-sulfane (2j):**

**Method A:** Ethynylbenzene (50 mg, 0.49 mmol), $SF_5Cl$ (0.96 M in hexanes, 0.76 mL, 0.73 mmol), and $Et_3B$ (1 M in THF, 49 μL, 0.049 mmol) were engaged in general procedure, Method A, to afford the title compound **2j** as colorless oil (23.6 mg, 0.089 mmol, 18%) after purification by flash chromatography using 100% pentane as the eluent. **Method B:** Ethynylbenzene (50 mg, 0.49 mmol), $SF_5Cl$ (0.96 M in hexanes, 1.53 mL, 1.47 mmol), and DICAB (9.6 mg, 0.049 mmol) were engaged in general procedure, Method B, to afford the title compound **2j** as colorless oil (30.2 mg, 0.11 mmol, 23%) after purification by flash chromatography using 100% pentane as the eluent. $^1H$ NMR (500 MHz, $CDCl_3$): δ (ppm) = 7.44 – 7.38 (m, 3H), 7.38 – 7.33 (m, 2H), 6.94 (p, *J* = 7.7 Hz, 1H); $^{19}F$ NMR (470 MHz, $CDCl_3$): δ (ppm) = 82.22 – 80.33 (m, 1F), 69.00 (dd, *J* = 152.8, 7.5 Hz, 4F). Analytical data were identical to those previously reported [6,8].

**((1*Z*,3*Z*)-4-Chloro-2,4-diphenylbuta-1,3-dien-1-yl)pentafluoro-λ⁶-sulfane (2k):**

**Method B:** Ethynylbenzene (50 mg, 0.49 mmol), $SF_5Cl$ (0.96 M in hexanes,

[8] Aït-Mohand, S.; Dolbier Jr, W. R. *Org. Lett.* **2002**, *4*, 3013-3015.

1.53 mL, 1.47 mmol), and DICAB (9.6 mg, 0.049 mmol) were engaged in general procedure, Method B, to afford the title compound **2k** as colorless oil (9.1 mg, 0.025 mmol, 5%) after purification by flash chromatography using 100% pentane as the eluent. $^{1}$H NMR (500 MHz, $CDCl_3$): δ (ppm) = 7.36 – 7.30 (m, 5H), 7.30 – 7.26 (m, 3H), 7.18 – 7.13 (m, 2H), 6.54 – 6.48 (m, 1H), 6.24 (p, *J* = 8.7 Hz, 1H); $^{19}$F NMR (470 MHz, $CDCl_3$): δ (ppm) = 84.44 – 82.31 (m, 1F), 68.45 (dd, *J* = 152.4, 8.6 Hz, 4F). Analytical data were identical to those previously reported. [6,8]

**(*E*)-(7-Chlorododec-6-en-6-yl)pentafluoro-λ$^6$-sulfane (2l): Method A:** Dodec-6-yne (50 mg, 0.30 mmol), $SF_5Cl$ (0.96 M in hexanes, 0.47 mL, 0.46 mmol), and $Et_3B$ (1 M in THF, 30 µL, 0.030 mmol) were engaged in general procedure, Method A, to afford the title compound **2l** as colorless oil (63.3 mg, 0.19 mmol, 65%) after purification by flash chromatography using 100% hexanes as the eluent. **Method B:** Dodec-6-yne (50 mg, 0.30 mmol), $SF_5Cl$ (0.96 M in hexanes, 0.94 mL, 0.90 mmol), and DICAB (5.9 mg, 0.030 mmol) were engaged in general procedure, Method B, to afford the title compound **2l** which could not be isolated. The yield was estimated using NMR analysis and 2-fluoro-4-nitrotoluene as the reference; yield: 17%. $^{1}$H NMR (500 MHz, $CDCl_3$): δ (ppm) = 2.73 – 2.66 (m, 2H), 2.62 – 2.51 (m, 2H), 1.66 – 1.55 (m, 4H), 1.40 – 1.18 (m, 8H), 0.90 (t, *J* = 7.0 Hz, 6H); $^{13}$C NMR (126 MHz, $CDCl_3$): δ (ppm) =152.9 (p, *J* = 12.0 Hz), 146.1 (p, *J* = 3.9 Hz), 38.5 (t, *J* = 3.2 Hz), 33.7 (t, *J* = 3.1 Hz), 31.9, 31.3, 27.8, 27.2, 22.4, 22.2, 14.0, 13.9; $^{19}$F NMR (470 MHz, $CDCl_3$): δ (ppm) = 87.1 (p, *J* = 148.1 Hz, 1F), 64.5 (d, *J* = 148.1 Hz, 4F); GC-MS (CI): m/z calcd for $C_{12}H_{22}ClF_5S$ $[M]^+$ 328.11 found

328.00 (under all conditions tested for high-resolution mass spectra [ESI (+), ESI (–), APPI], no significant ion was detected); IR (ATR, Diamond): ν ($cm^{-1}$) = 2959, 2864, 1618, 1460, 1040, 824, 766, 648.

**Full optimization results**

1. **Evaluation of the amino-borane complexes**

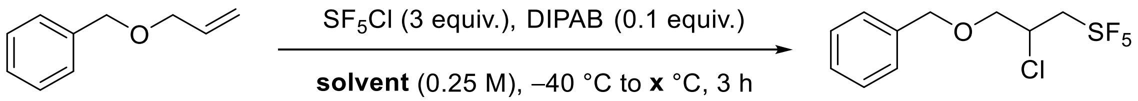

1

2a

| Entry | Solvent | x (°C) | Conversion (%)[a] | Yield (%)[b] |
|---|---|---|---|---|
| 1 | hexane | 30 | 28 | 2 |
| 2 | hexane | 40 | 32 | 6 |
| 3 | hexane | 50 | 33 | 7 |
| 4 | hexane | 60 | 55 | 5 |
| 5 | EtOAc | 30 | 100 | 72 |
| 6 | EtOAc | 40 | 100 | 64 |
| 7 | EtOAc | 50 | 100 | 5 |
| 8 | EtOAc | 60 | 100 | 29 |
| 9 | MTBE | 30 | 77 | 40 |
| 10 | MTBE | 40 | 69 | 6 |
| 11 | MTBE | 50 | 79 | 41 |
| 12 | MTBE | 60 | 92 | 26 |
| 13 | THF | 30 | 100 | 28 |
| 14 | THF | 40 | 100 | 33 |
| 15 | THF | 50 | 100 | 28 |
| 16 | THF | 60 | 100 | 30 |
| 17 | toluene | 30 | 39 | 14 |
| 18 | toluene | 40 | 41 | 5 |
| 19 | toluene | 50 | 46 | 8 |
| 20 | toluene | 60 | 83 | 41 |
| 21 | MeOH | 30 | 100 | 0 |
| 22 | MeOH | 40 | 100 | traces |
| 23 | MeOH | 50 | 100 | traces |
| 24 | MeOH | 60 | 100 | 0 |
| 25 | acetone | 30 | 100 | traces |
| 26 | acetone | 40 | 100 | 0 |
| 27 | acetone | 50 | 100 | 9 |
| 28 | $CH_2Cl_2$ | 30 | 15 | traces |

[a]Disappearance of the starting material, estimated by $^{1}H$ NMR analysis of the crude mixture using 2-fluoro-4-nitrotoluene as an internal standard. [b]Yield estimated by $^{19}F$ NMR analysis of the crude mixture using 2-fluoro-4-nitrotoluene as an internal standard.

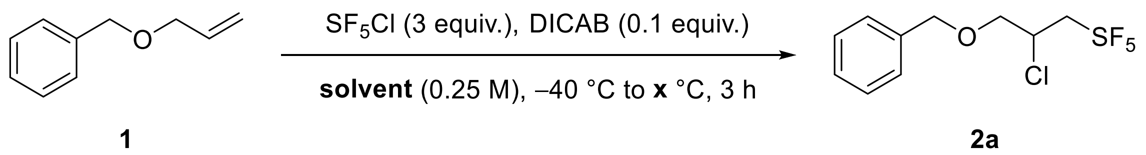

| Entry | Solvent | x (°C) | Conversion (%)[a] | Yield (%)[b] |
|---|---|---|---|---|
| 1 | hexane | 30 | 25 | 1 |
| 2 | hexane | 40 | 17 | 2 |
| 3 | hexane | 50 | 82 | 72 |
| 4 | hexane | 60 | 75 | 32 |
| 5 | EtOAc | 30 | 100 | traces |
| 6 | EtOAc | 40 | 100 | 4 |
| 7 | EtOAc | 50 | 100 | 62 |
| 8 | EtOAc | 60 | 100 | 56 |
| 9 | MTBE | 30 | 49 | 3 |
| 10 | MTBE | 40 | 100 | 65 |
| 11 | MTBE | 50 | 94 | 21 |
| 12 | MTBE | 60 | 100 | 86 |
| 13 | THF | 30 | 100 | 43 |
| 14 | THF | 40 | 100 | 27 |
| 15 | THF | 50 | 100 | 38 |
| 16 | THF | 60 | 100 | 20 |
| 17 | toluene | 30 | 58 | 37 |
| 18 | toluene | 40 | 53 | 39 |
| 19 | toluene | 50 | 50 | 23 |
| 20 | toluene | 60 | 71 | 33 |
| 21 | MeOH | 30 | 100 | traces |
| 22 | MeOH | 40 | 100 | traces |
| 23 | MeOH | 50 | 100 | traces |
| 24 | MeOH | 60 | 100 | traces |
| 25 | acetone | 30 | 100 | 1 |
| 26 | acetone | 40 | 100 | 0 |
| 27 | acetone | 50 | 100 | traces |
| 28 | $CH_2Cl_2$ | 30 | 24 | traces |

[a]Disappearance of the starting material, estimated by $^1H$ NMR analysis of the crude mixture using 2-fluoro-4-nitrotoluene as an internal standard. [b]Yield estimated by $^{19}F$ NMR analysis of the crude mixture using 2-fluoro-4-nitrotoluene as an internal standard.

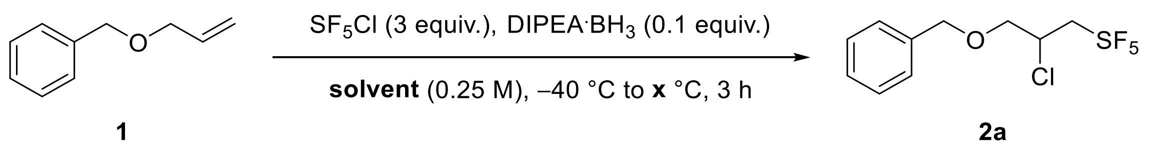

| Entry | solvent | x (°C) | Conversion (%)[a] | Yield (%)[b] |
|---|---|---|---|---|
| 1 | hexane | 30 | 32 | traces |
| 2 | hexane | 40 | 41 | 8 |
| 3 | hexane | 50 | 47 | 10 |
| 4 | hexane | 60 | 58 | 10 |
| 5 | EtOAc | 30 | 100 | 18 |
| 6 | EtOAc | 40 | 100 | 58 |
| 7 | EtOAc | 50 | 100 | 8 |
| 8 | EtOAc | 60 | 100 | 4 |
| 9 | THF | 30 | 100 | 6 |
| 10 | THF | 40 | 100 | 19 |
| 11 | THF | 50 | 100 | 24 |
| 12 | THF | 60 | 100 | 32 |
| 13 | toluene | 30 | 71 | 23 |
| 14 | toluene | 40 | 78 | 44 |
| 15 | toluene | 50 | 53 | 10 |
| 16 | toluene | 60 | 53 | 1 |

[a]Disappearance of the starting material, estimated by $^{1}$H NMR analysis of the crude mixture using 2-fluoro-4-nitrotoluene as an internal standard. [b]Yield estimated by $^{19}$F NMR analysis of the crude mixture using 2-fluoro-4-nitrotoluene as an internal standard.

## 2. Evaluation of the increase in the amount of DICAB and the reaction time

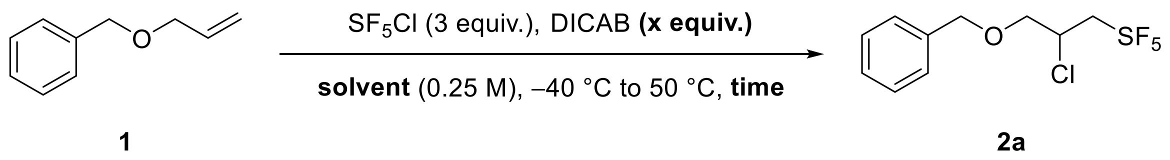

| Solvent | DICAB equiv. | Time (h) | Conversion (%)[a] | Yield (%)[b] |
|---|---|---|---|---|
| EtOAc | 0.2 | 3 | 100 | 80 |
| EtOAc | 0.1 | 6 | 100 | 86 |
| hexane | 0.2 | 3 | 61 | 37 |
| hexane | 0.1 | 6 | 94 | 89 |
| hexane | 0 | 6 | 20 | traces |

[a]Disappearance of the starting material, estimated by $^{1}$H NMR analysis of the crude mixture using 2-fluoro-4-nitrotoluene as an internal standard. [b]Yield estimated by $^{19}$F NMR analysis of the crude mixture using 2-fluoro-4-nitrotoluene as an internal standard.

### 3. Evaluation of the decreased in the amount of amino-borane complex

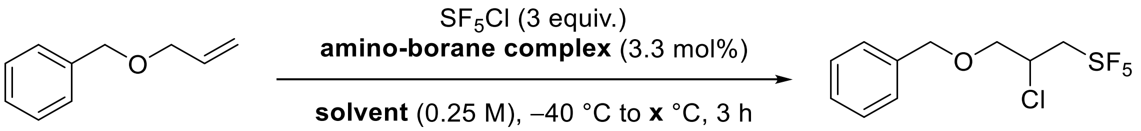


**1** **2a**

| Entry | solvent | amino-borane complex | x (°C) | Conversion (%)[a] | Yield (%)[b] |
|---|---|---|---|---|---|
| 1 | hexane | DIPAB | 30 | 17 | traces |
| 2 | hexane | DIPAB | 40 | 19 | traces |
| 3 | hexane | DIPAB | 50 | 25 | 2 |
| 4 | hexane | DIPAB | 60 | 65 | 41 |
| 5 | hexane | DICAB | 30 | 14 | traces |
| 6 | hexane | DICAB | 40 | 17 | 2 |
| 7 | hexane | DICAB | 50 | 93 | 86 |
| 8 | hexane | DICAB | 60 | 30 | 22 |
| 9 | EtOAc | DIPAB | 30 | 21 | traces |
| 10 | EtOAc | DIPAB | 40 | 70 | traces |
| 11 | EtOAc | DIPAB | 50 | 54 | 9 |
| 12 | EtOAc | DIPAB | 60 | 100 | 6 |
| 13 | EtOAc | DICAB | 30 | 38 | 1 |
| 14 | EtOAc | DICAB | 40 | 100 | 1 |
| 15 | EtOAc | DICAB | 50 | 66 | 2 |
| 16 | EtOAc | DICAB | 60 | 100 | 37 |
| 17 | MTBE | DIPAB | 30 | 30 | 1 |
| 18 | MTBE | DIPAB | 40 | 78 | 33 |
| 19 | MTBE | DIPAB | 50 | 67 | 20 |
| 20 | MTBE | DIPAB | 60 | 67 | 24 |
| 21 | MTBE | DICAB | 30 | 58 | 27 |
| 22 | MTBE | DICAB | 40 | 100 | 77 |
| 23 | MTBE | DICAB | 50 | 100 | 73 |
| 24 | MTBE | DICAB | 60 | 100 | 75 |

[a]Disappearance of the starting material, estimated by $^{1}$H NMR analysis of the crude mixture using 2-fluoro-4-nitrotoluene as an internal standard. [b]Yield estimated by $^{19}$F NMR analysis of the crude mixture using 2-fluoro-4-nitrotoluene as an internal standard.

## 4. Evaluation of the initial temperature

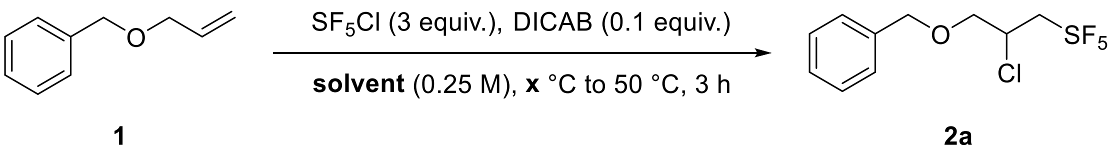


| Solvent | x °C | Conversion (%)[a] | Yield (%)[b] |
|---|---|---|---|
| EtOAc | 0 | 100 | 93 |
| EtOAc | rt | 100 | 71 |
| hexane | 0 | 40 | 2 |
| hexane | rt | 100 | 81 |
| MTBE | 0 | 100 | 75 |
| MTBE | rt | 100 | 79 |

[a]Disappearance of the starting material, estimated by $^{1}$H NMR analysis of the crude mixture using 2-fluoro-4-nitrotoluene as an internal standard. [b]Yield estimated by $^{19}$F NMR analysis of the crude mixture using 2-fluoro-4-nitrotoluene as an internal standard.

## NMR spectra

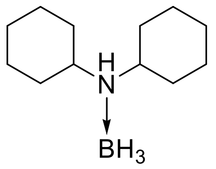

**DICAB**

$^{11}B$ NMR (160 MHz, $CDCl_3$)

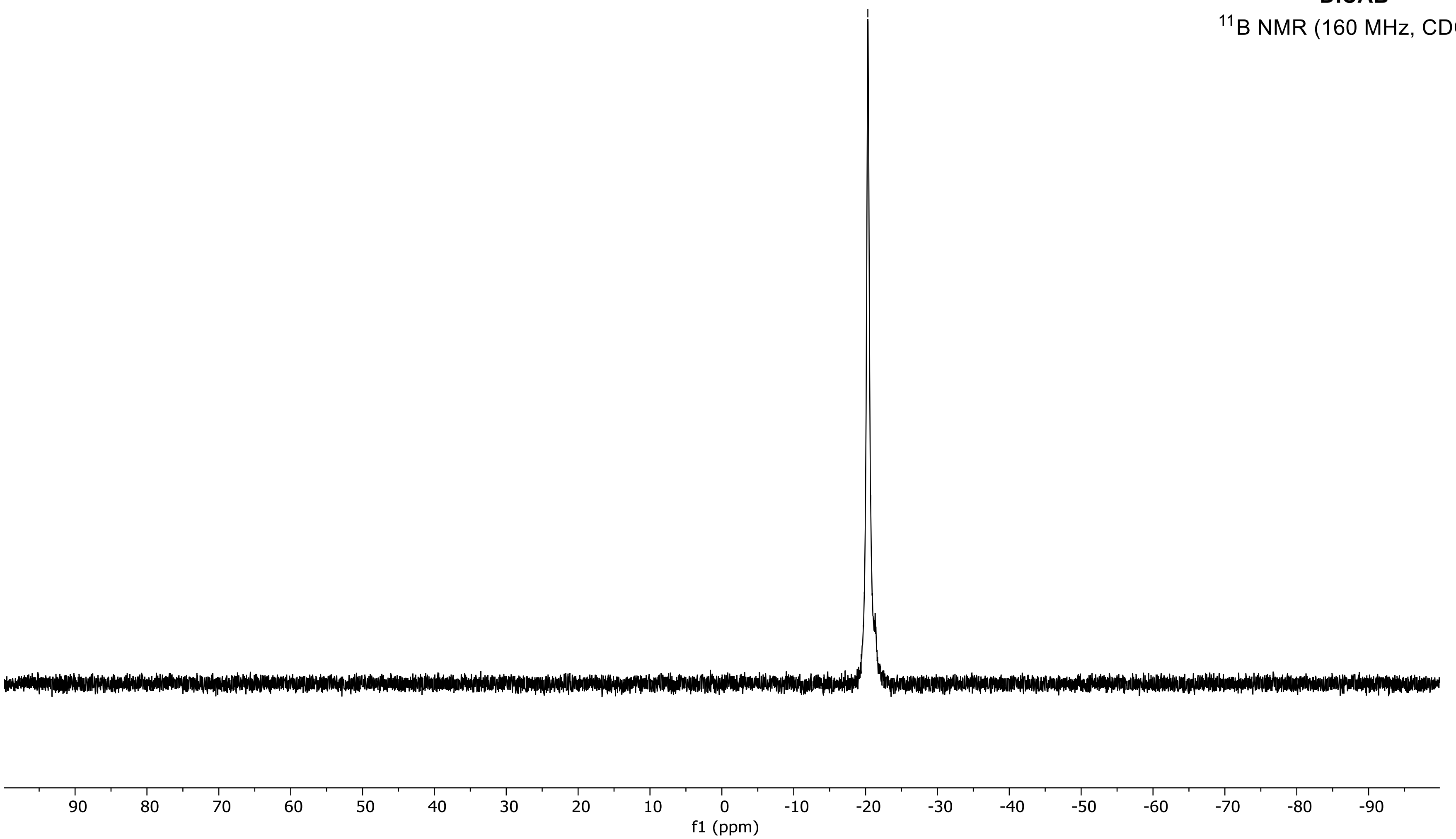

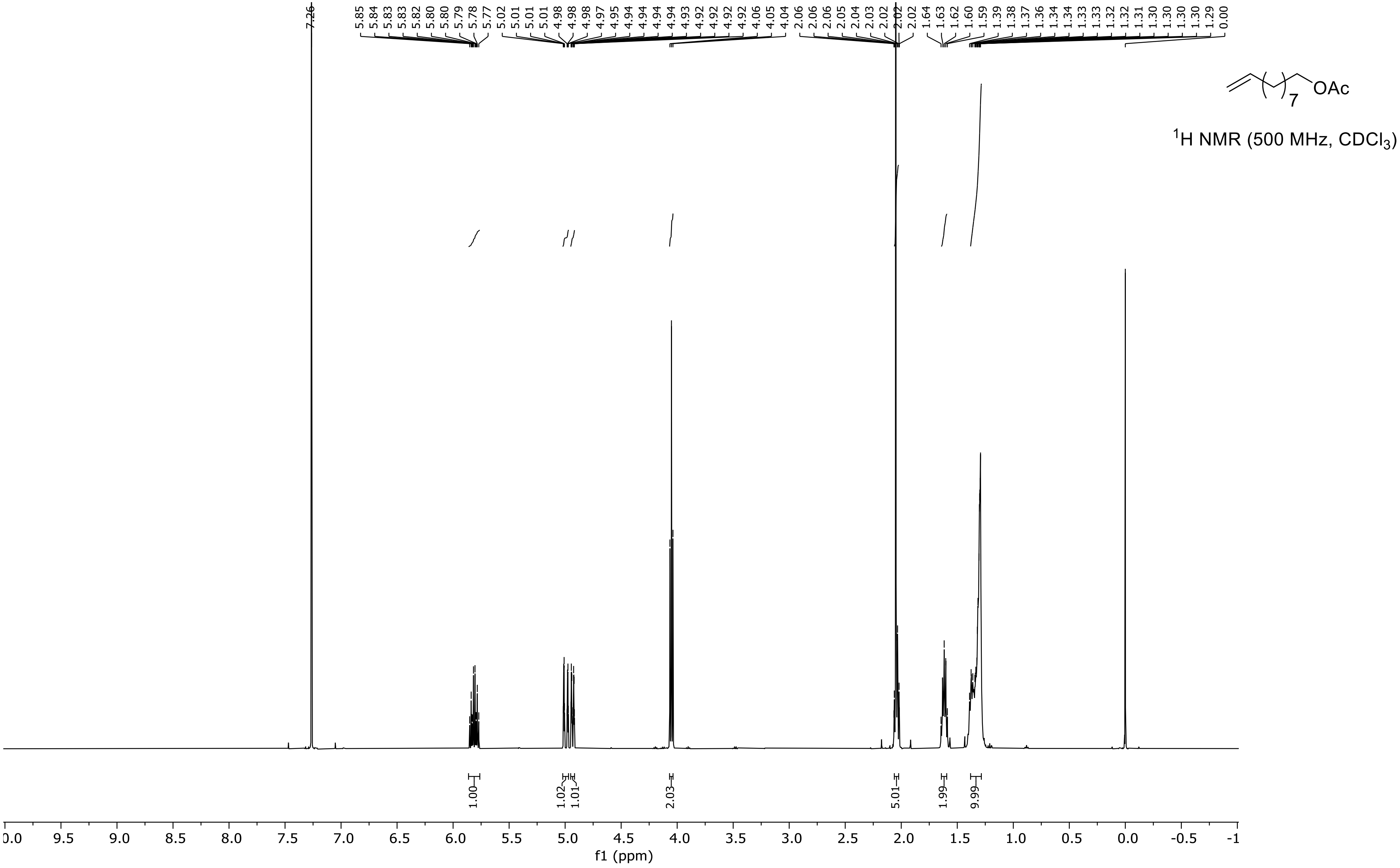
OAc
7
$^1$H NMR (500 MHz, $CDCl_3$)
f1 (ppm)

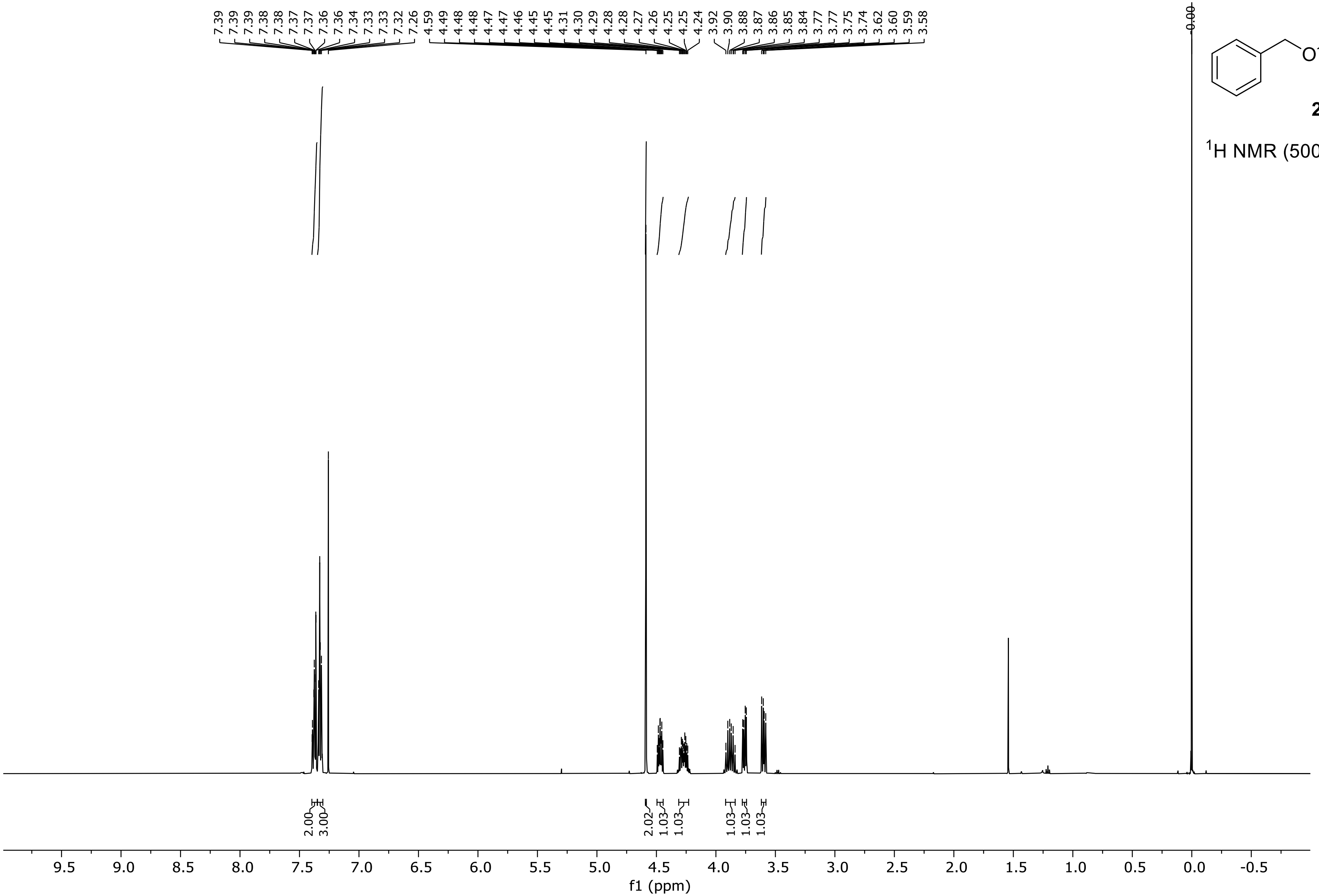


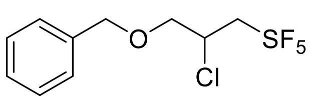


**2a**

$^1$H NMR (500 MHz, $CDCl_3$)

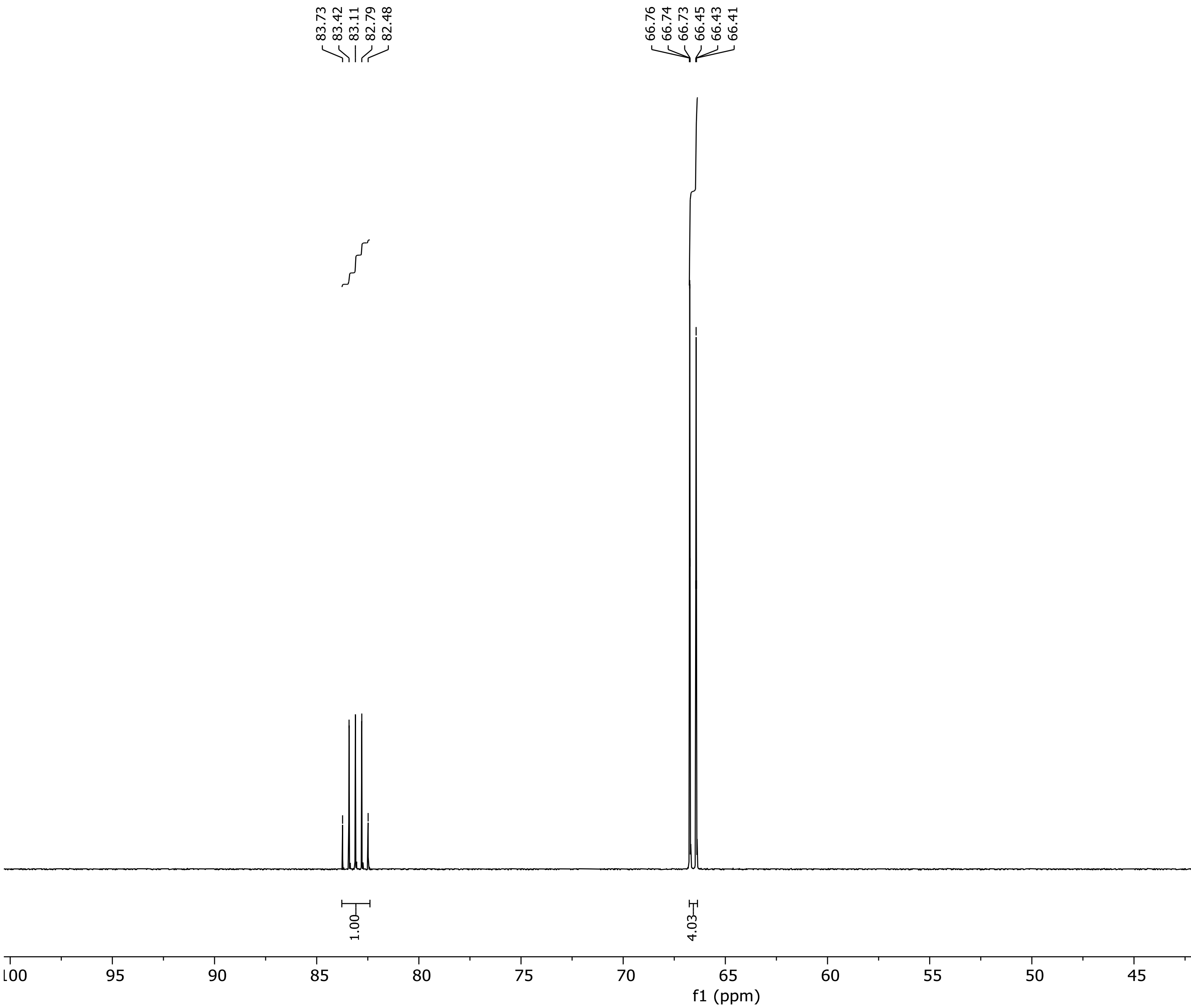


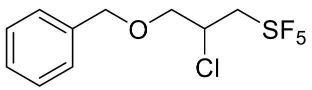


**2a**

$^{19}$F NMR (470 MHz, $CDCl_3$)

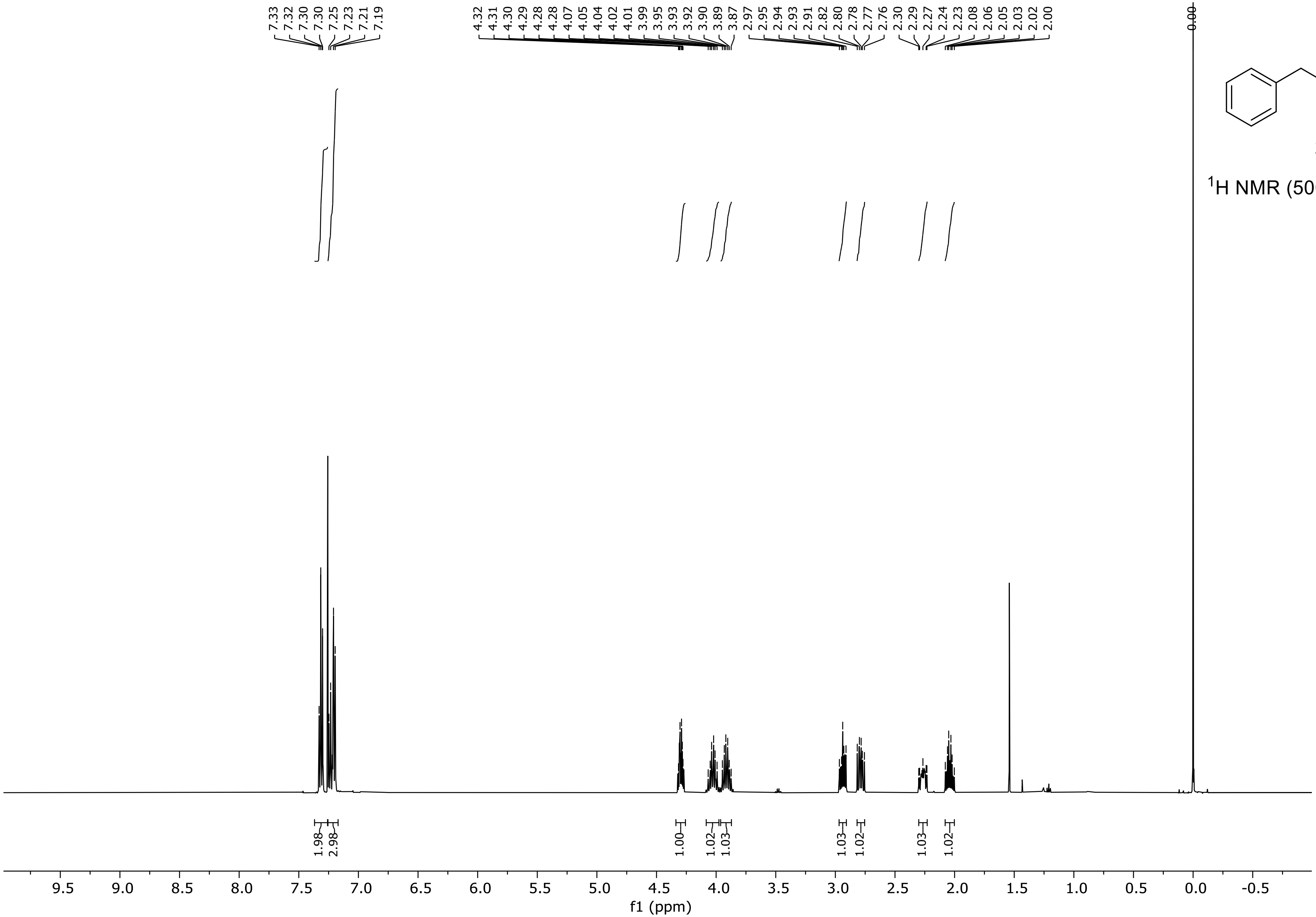

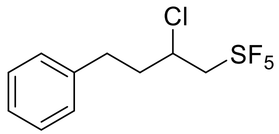

**2b**

$^{1}H$ NMR (500 MHz, $CDCl_3$)

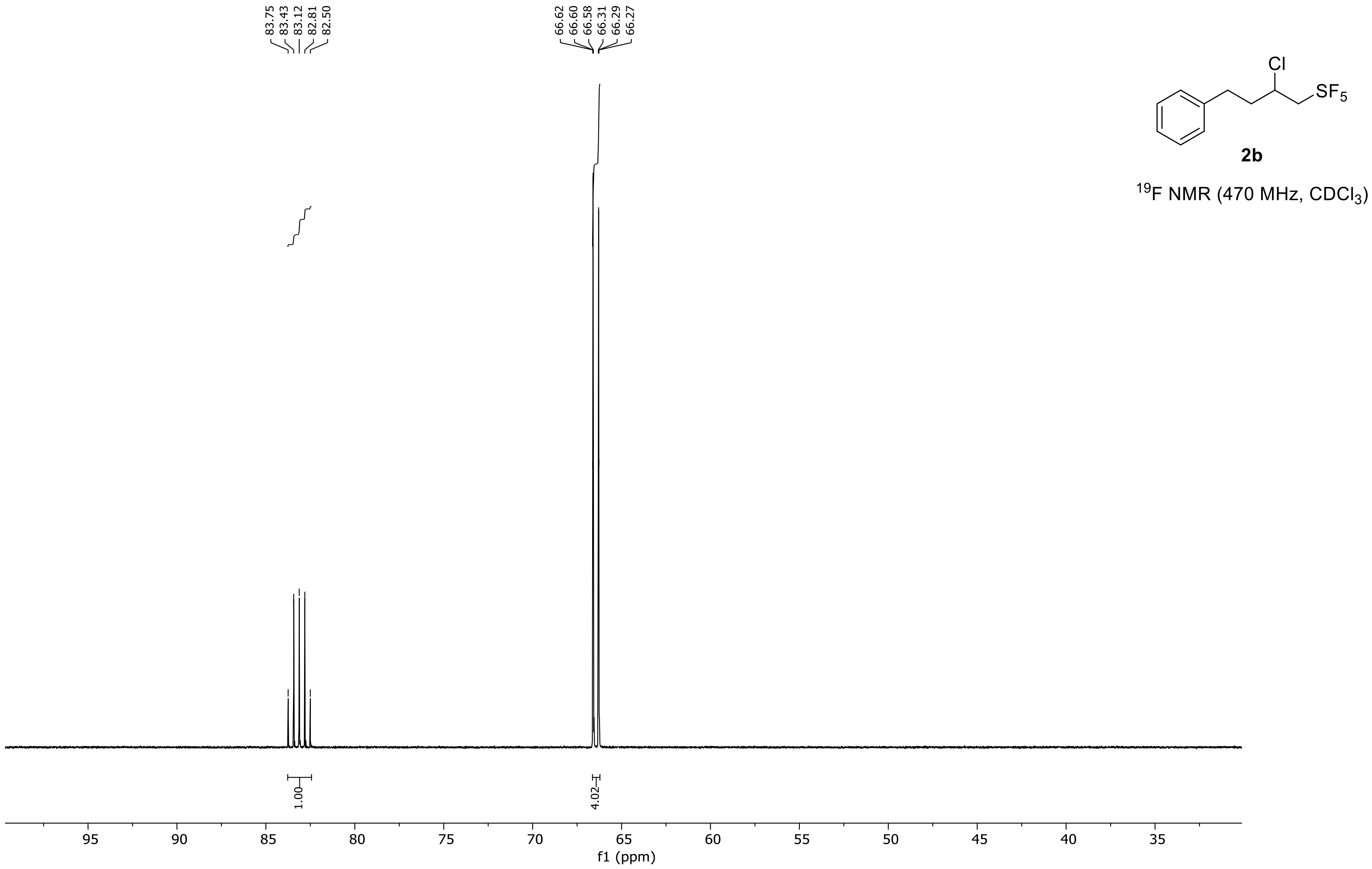
83.75
83.43
83.12
82.81
82.50
66.62
66.60
66.58
66.31
66.29
66.27
Cl
SF5
2b
19F NMR (470 MHz, CDCl3)
1.00
4.02
95 90 85 80 75 70 65 60 55 50 45 40 35
f1 (ppm)

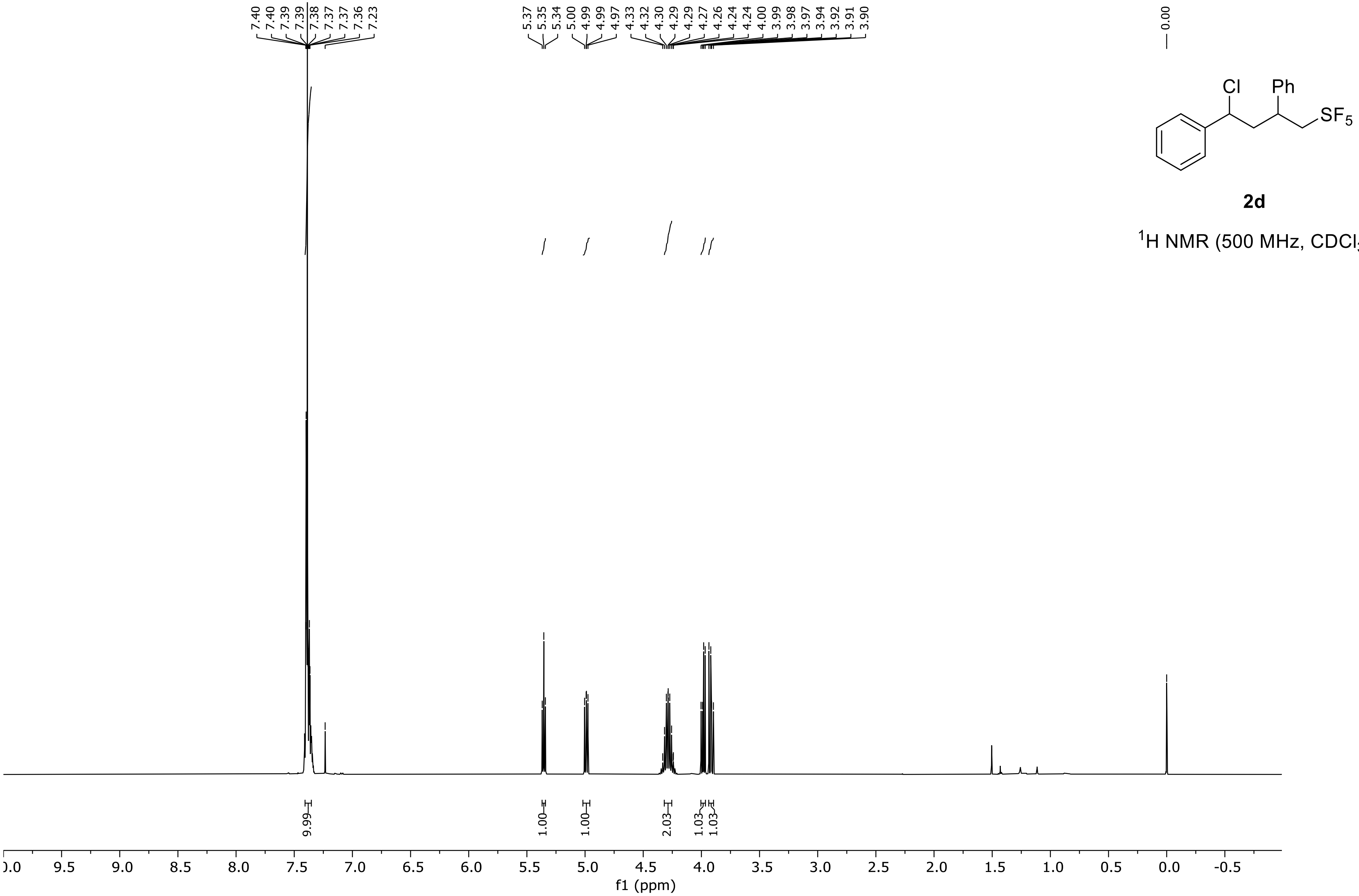


**2d**

$^1$H NMR (500 MHz, $CDCl_3$)

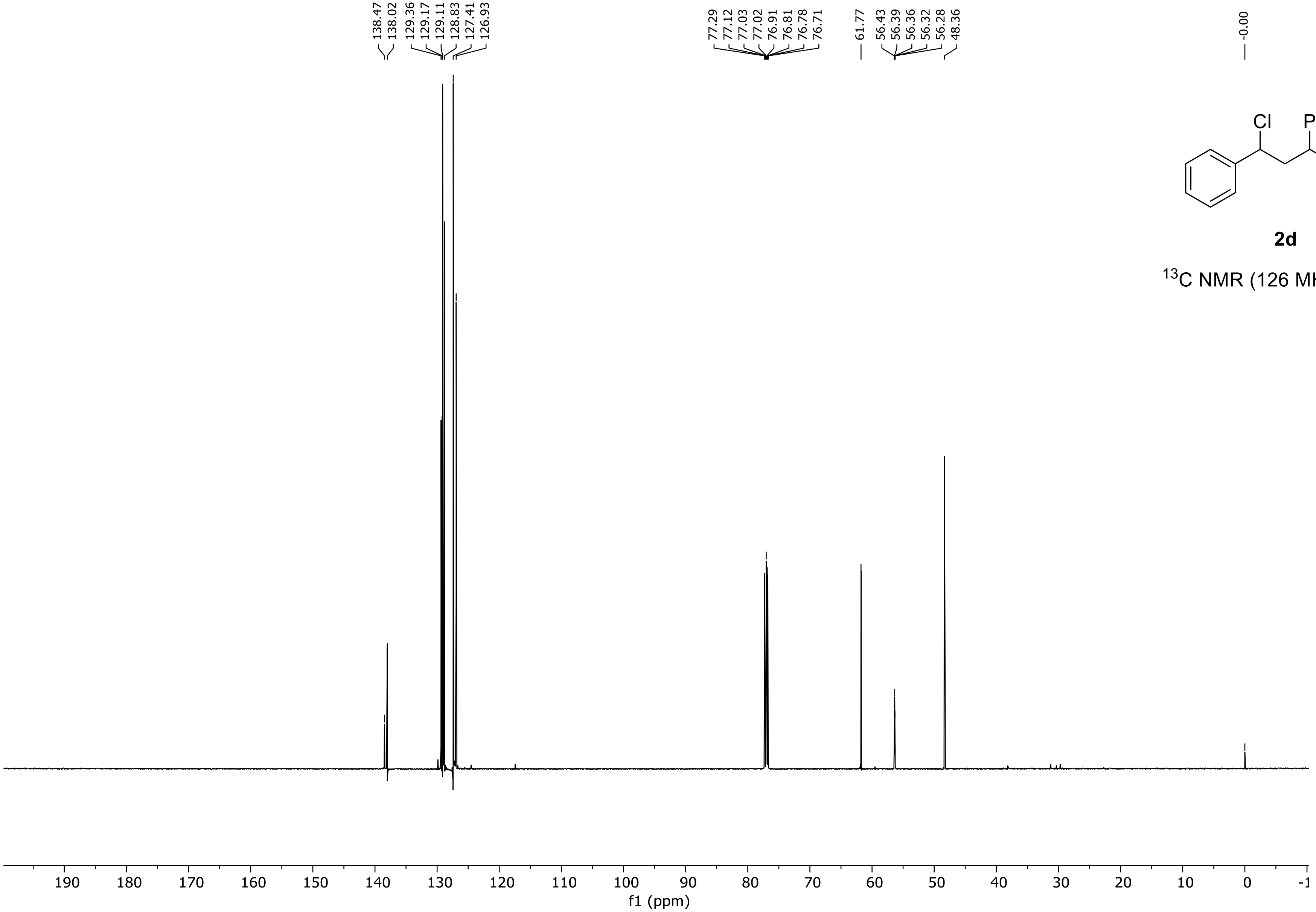


**2d**

$^{13}$C NMR (126 MHz, $CDCl_3$)

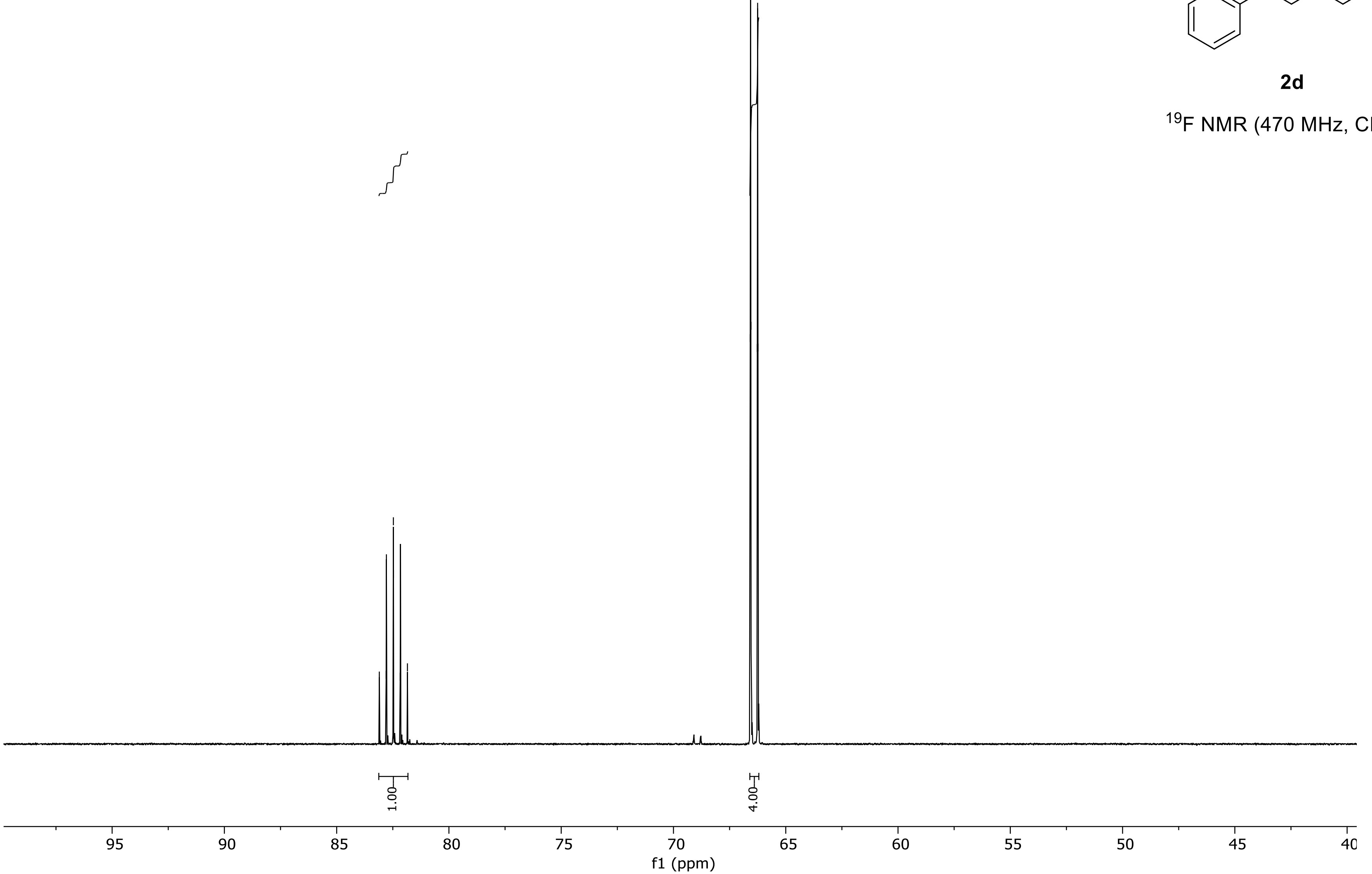


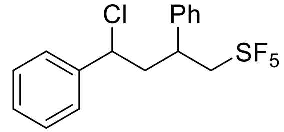


**2d**

$^{19}F$ NMR (470 MHz, $CDCl_3$)

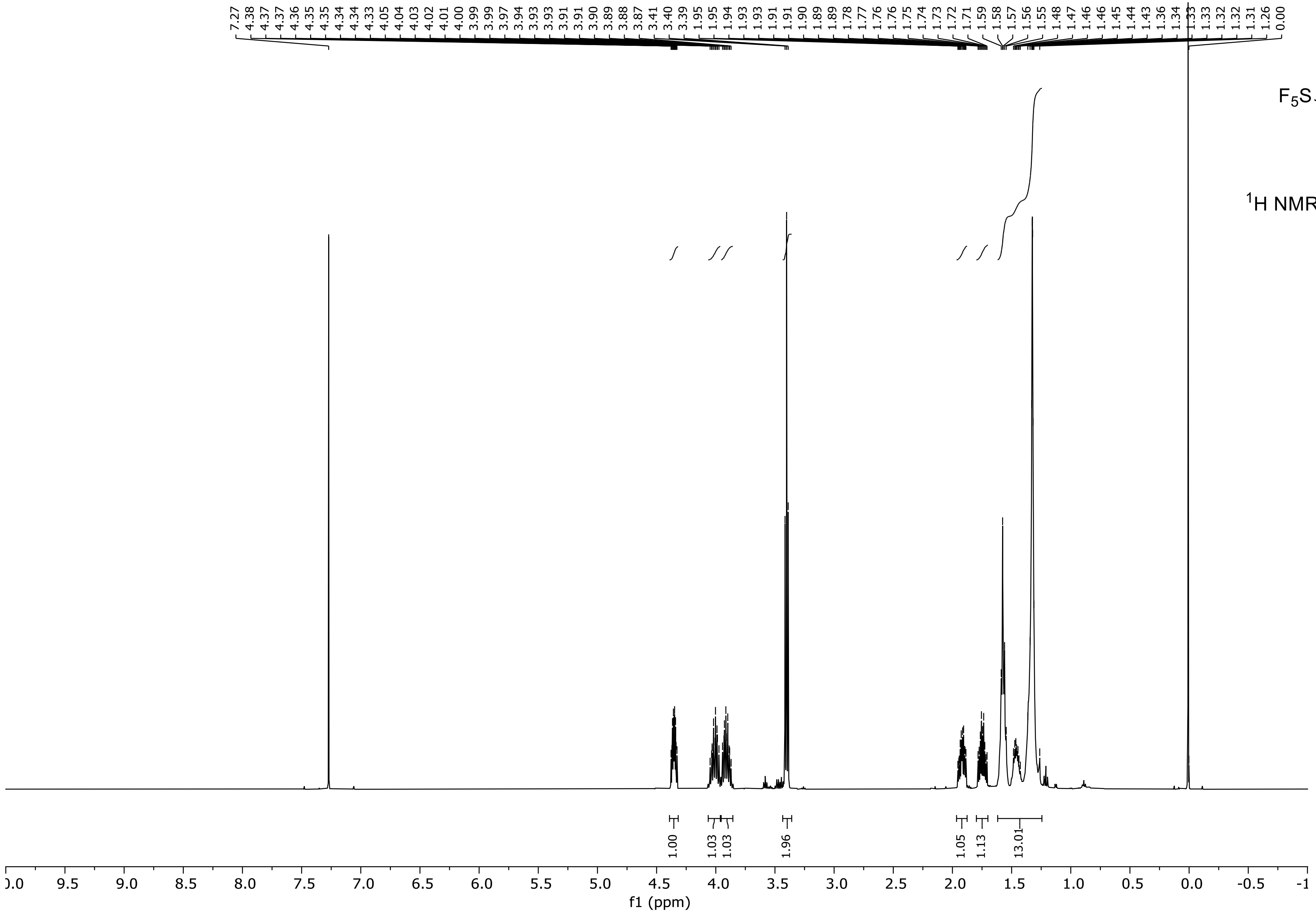


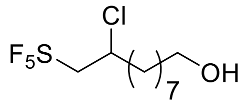

**2e**

$^1$H NMR (500 MHz, $CDCl_3$)

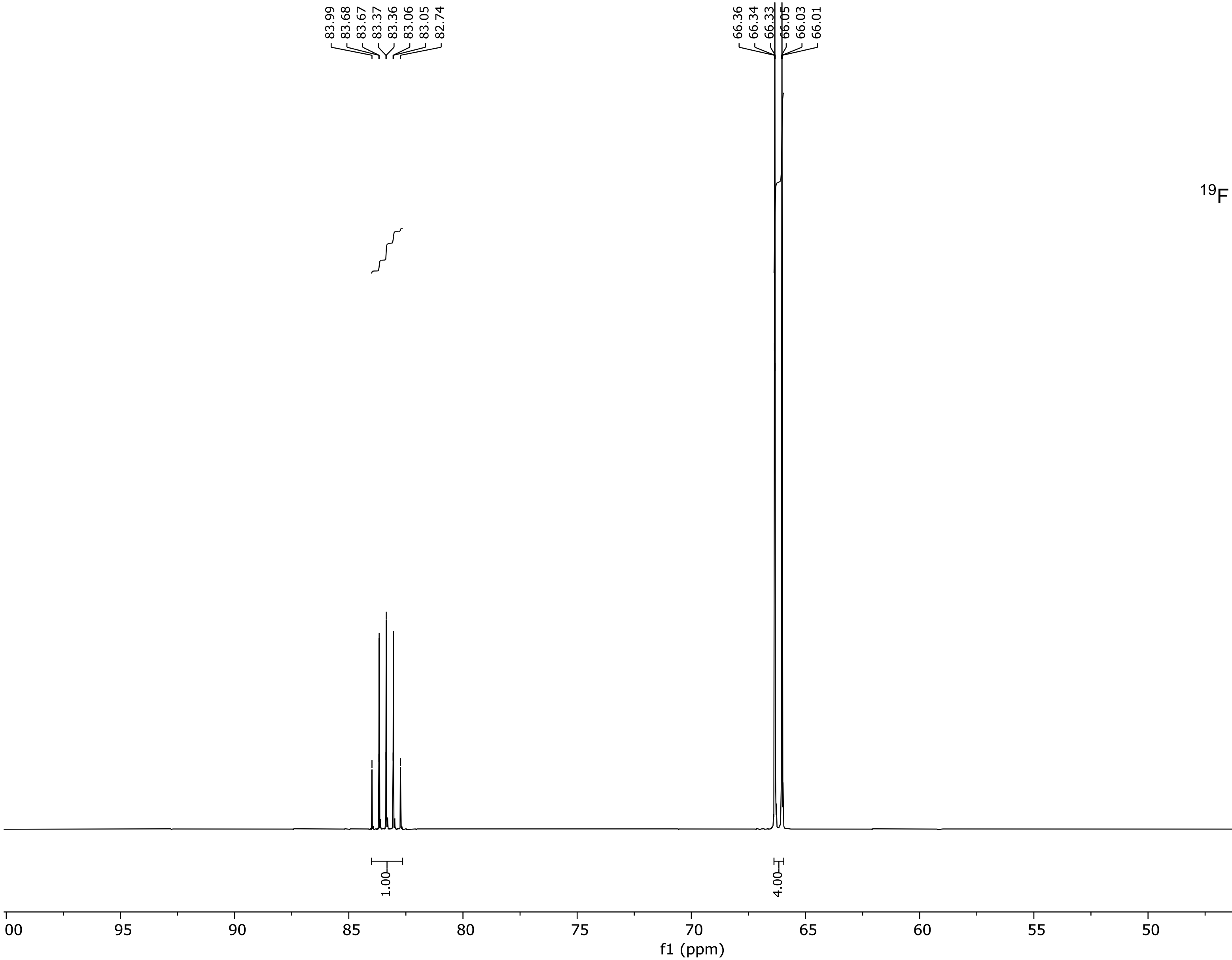


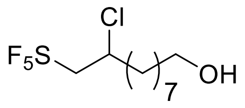


**2e**

$^{19}$F NMR (470 MHz, CDCl$_3$)

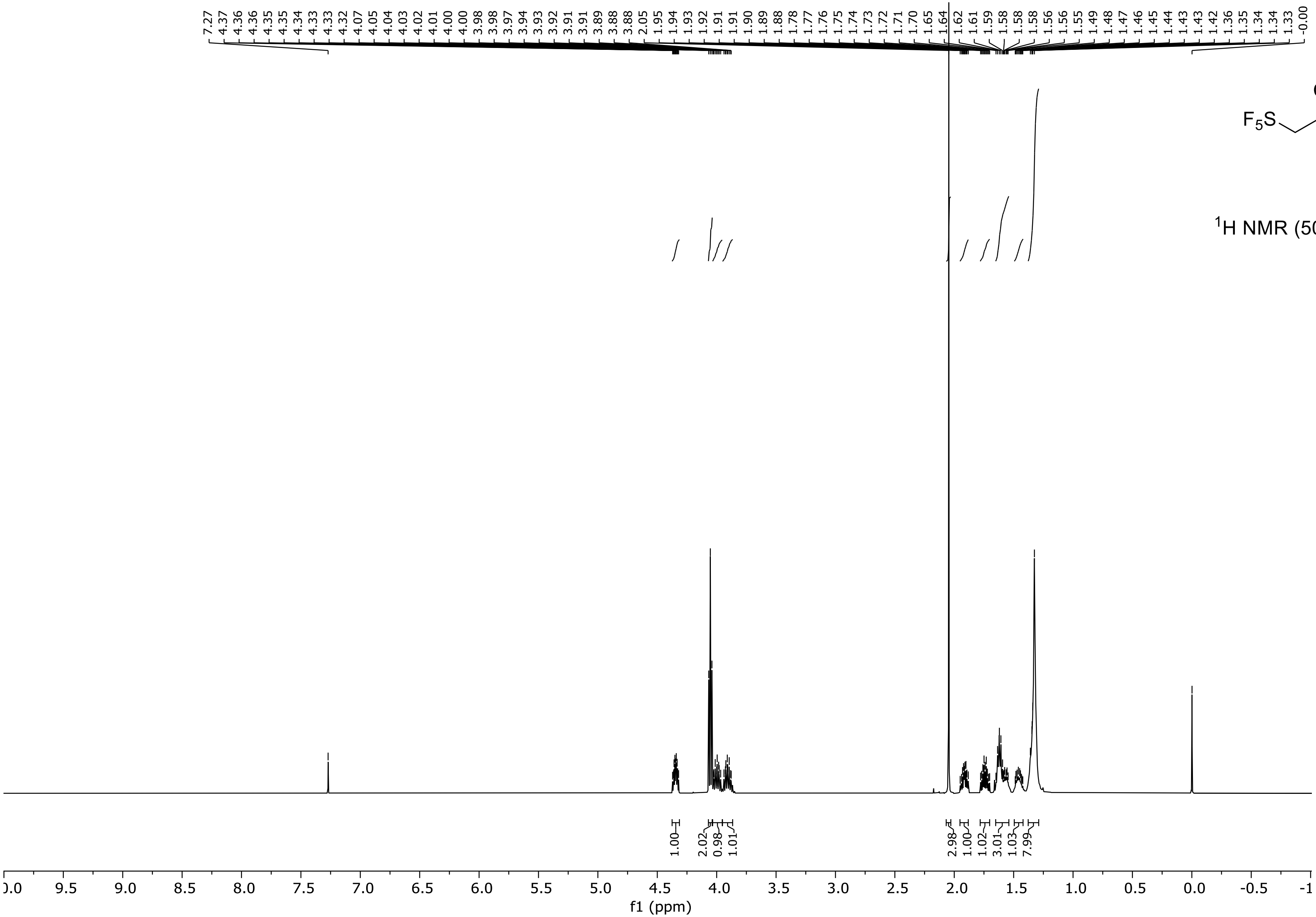


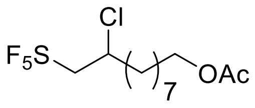


**2f**

$^{1}$H NMR (500 MHz, $CDCl_3$)

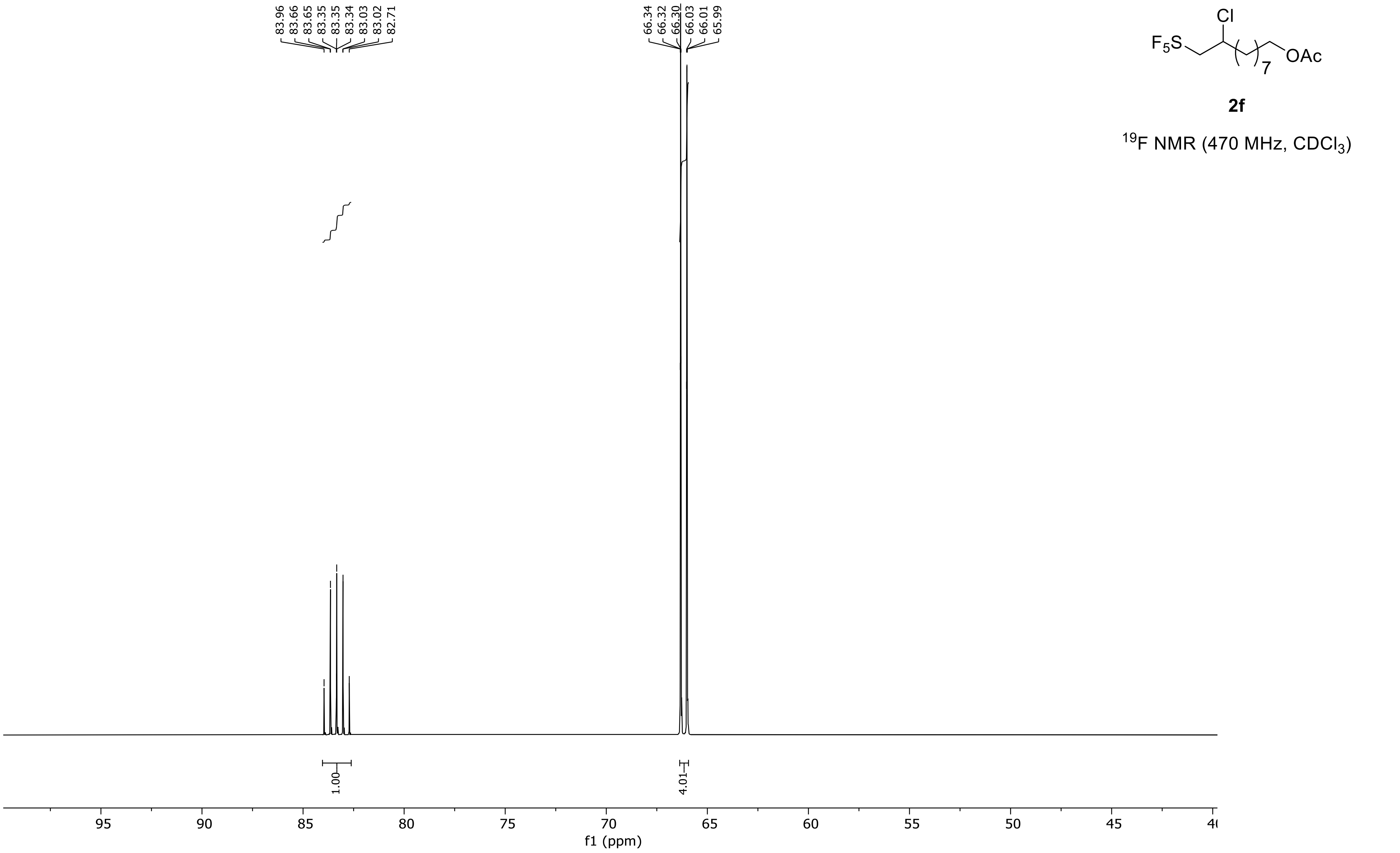


**2f**

$^{19}$F NMR (470 MHz, $CDCl_3$)

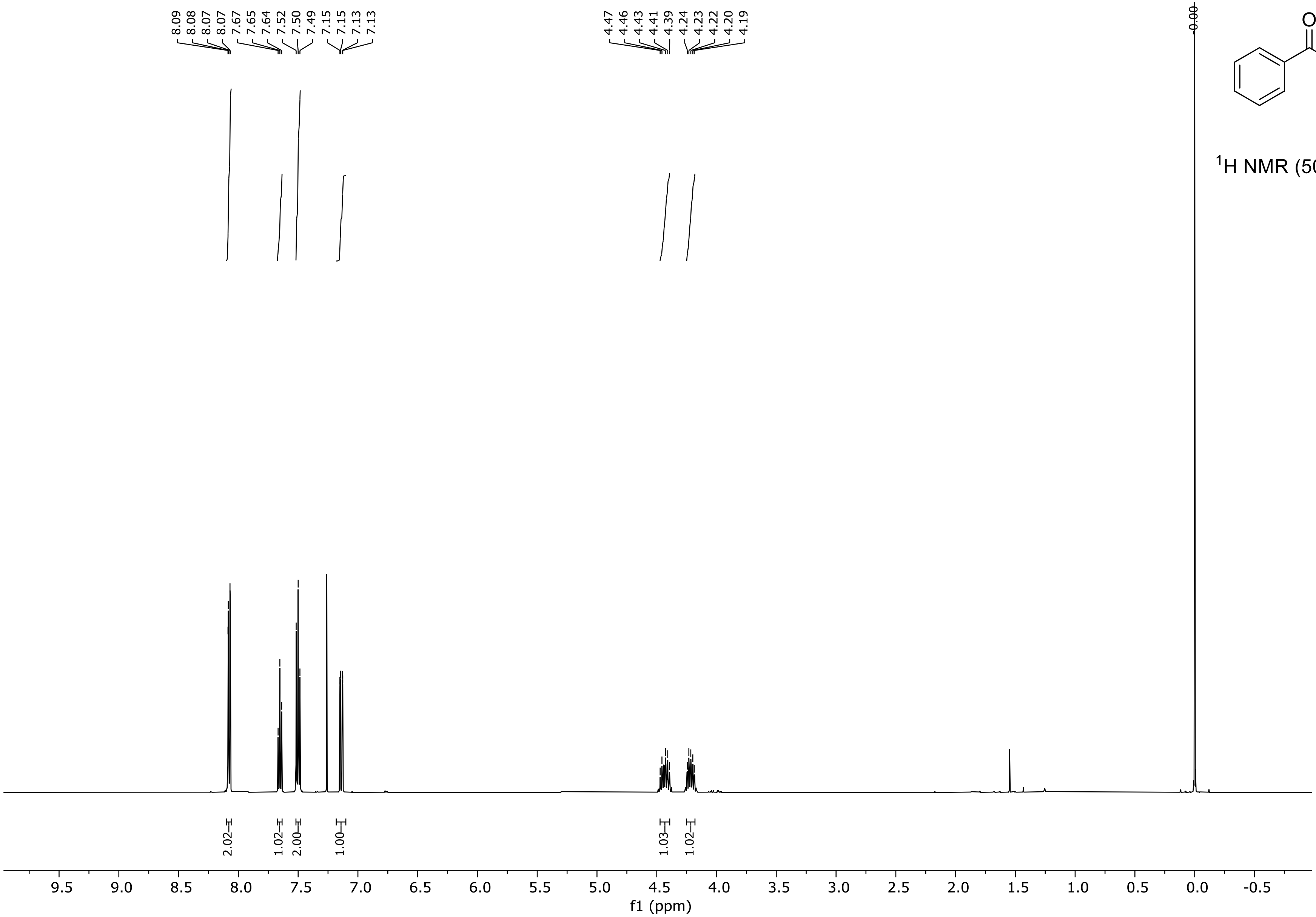


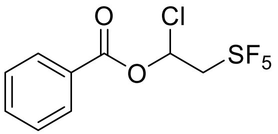


**2g**

$^1H$ NMR (500 MHz, $CDCl_3$)

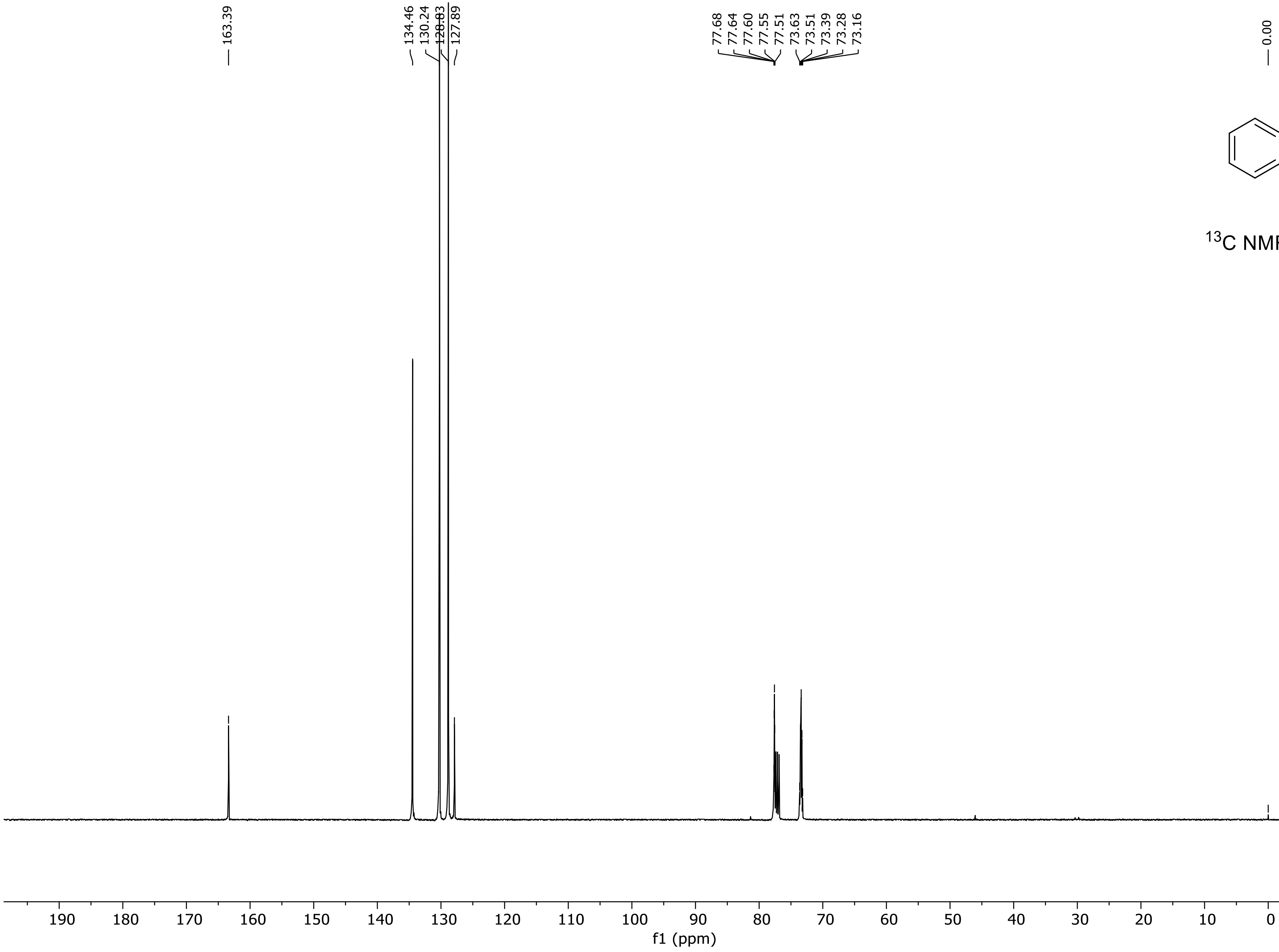


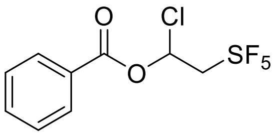


**2g**

$^{13}C$ NMR (126 MHz, $CDCl_3$)

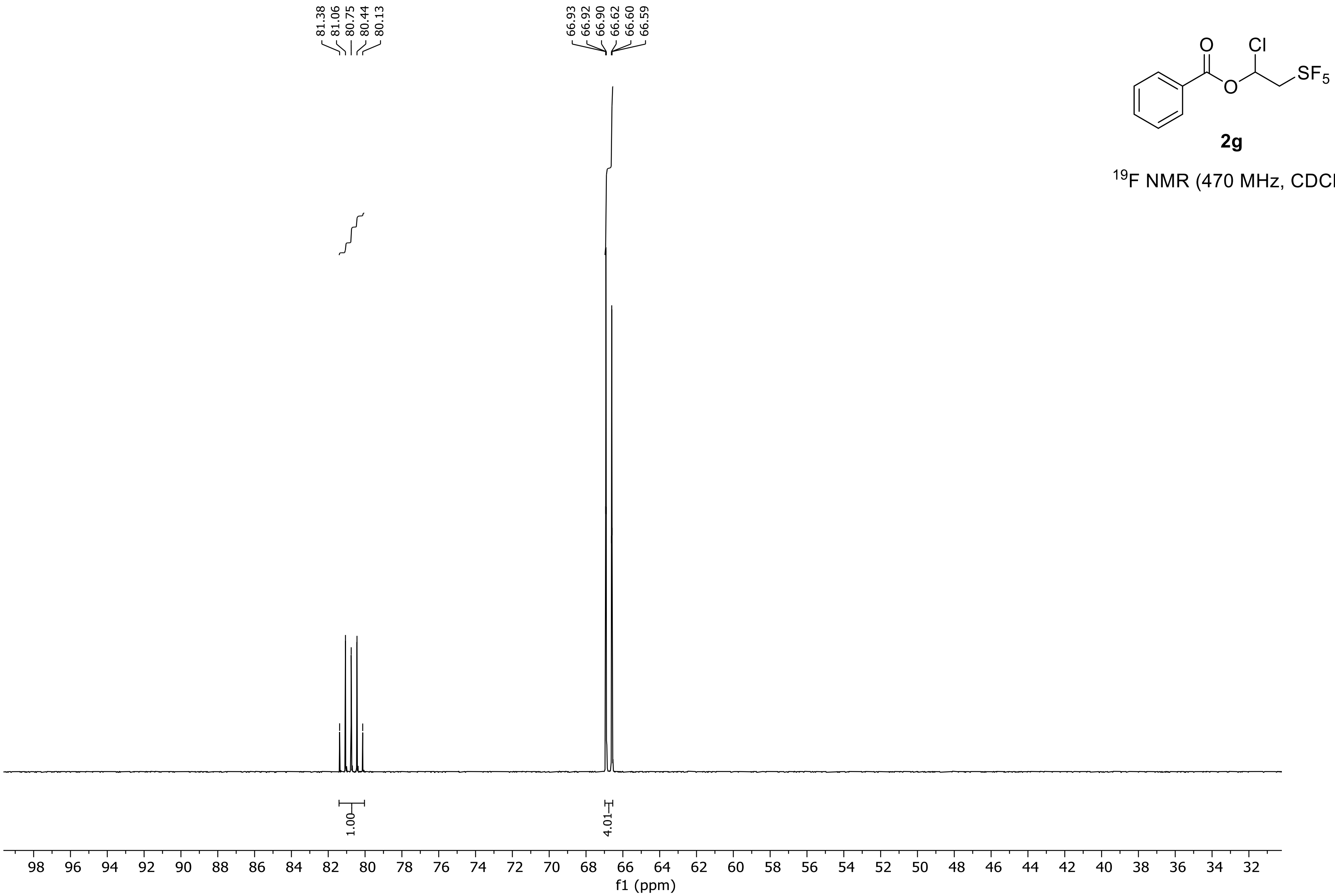


**2g**

$^{19}$F NMR (470 MHz, $CDCl_3$)

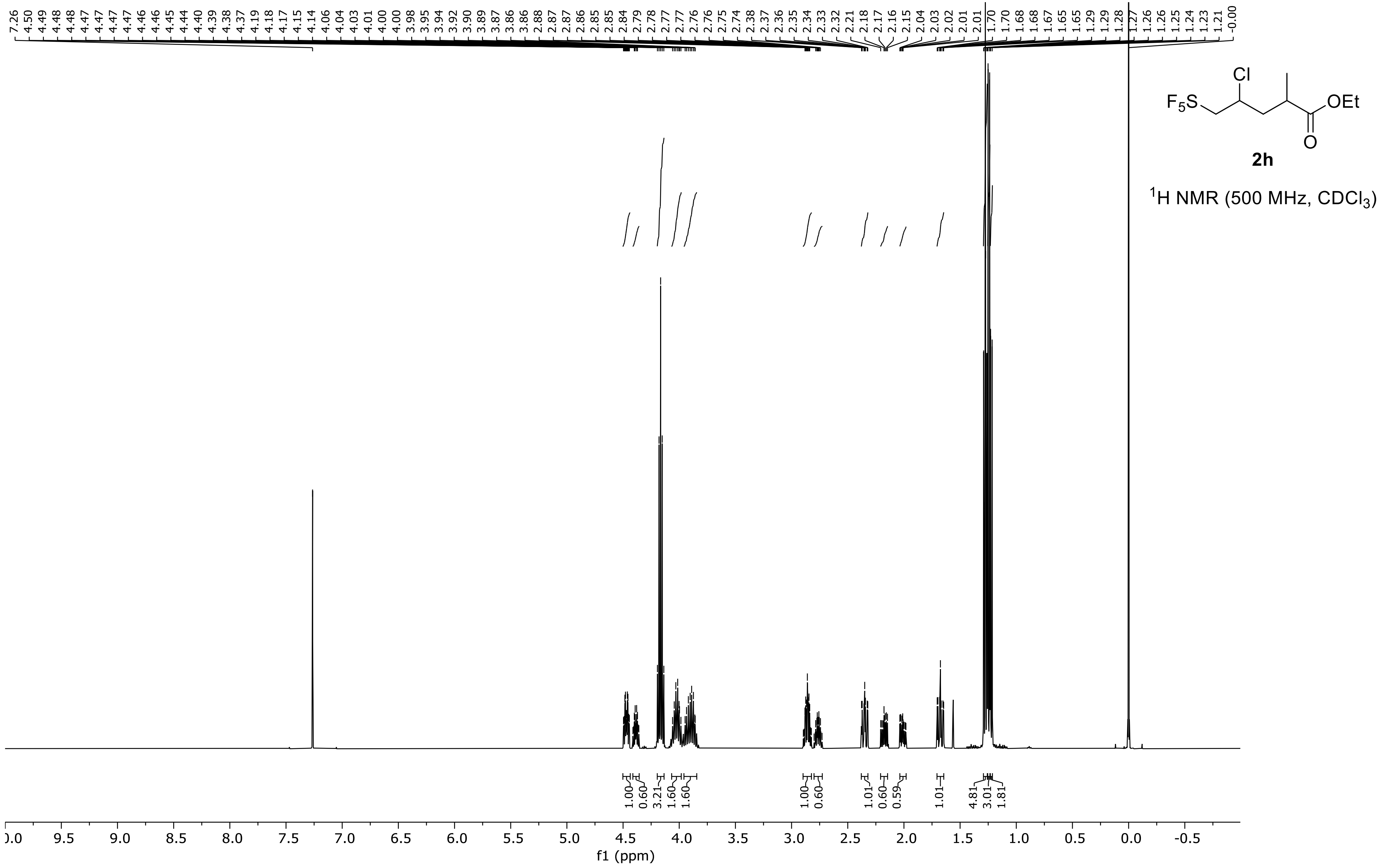

Cl
$F_5S$
OEt
O
2h
$^1H$ NMR (500 MHz, $CDCl_3$)
f1 (ppm)

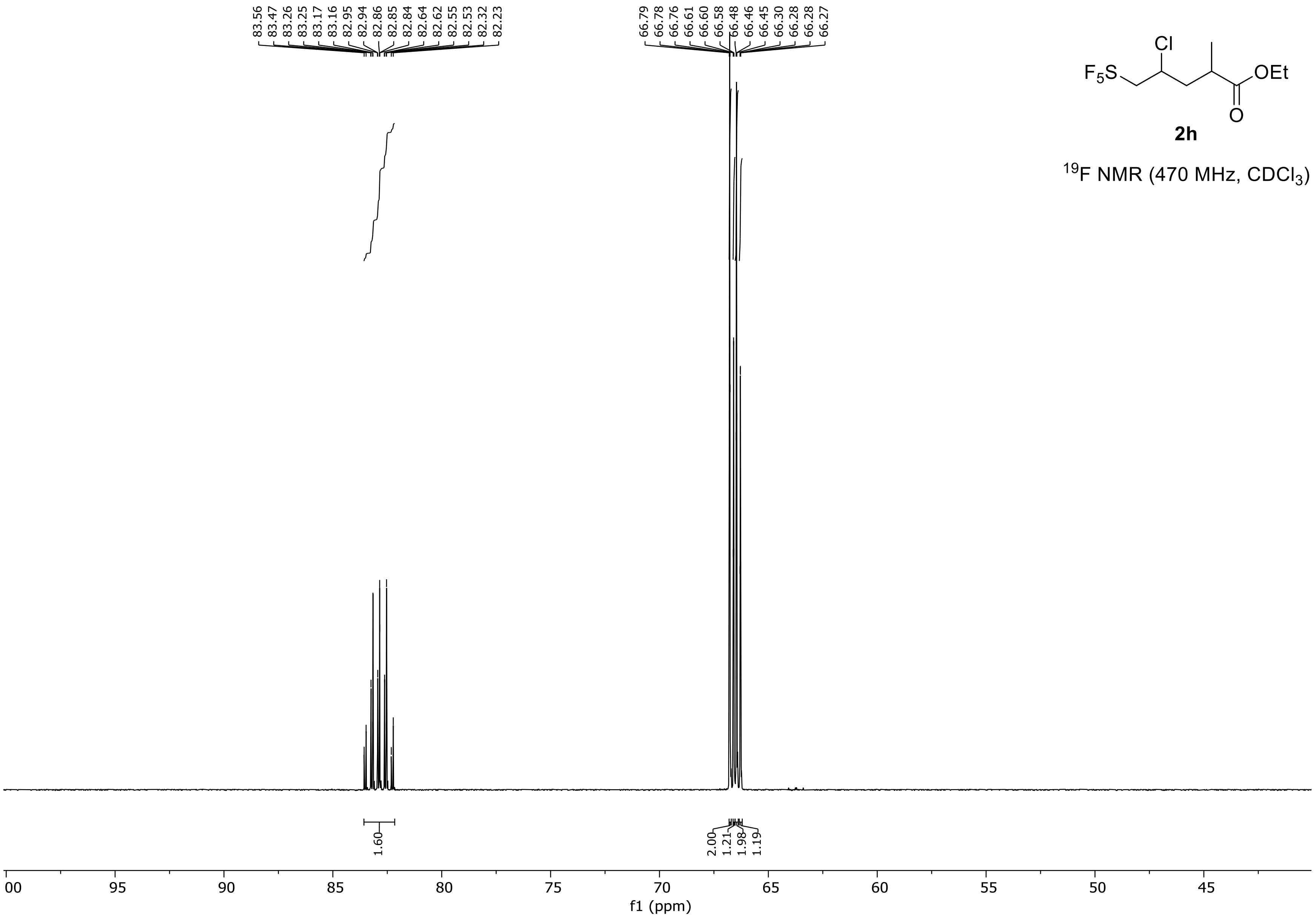

83.56 83.47 83.26 83.25 83.17 83.16 82.95 82.94 82.86 82.85 82.84 82.64 82.62 82.55 82.53 82.32 82.23
66.79 66.78 66.76 66.61 66.60 66.58 66.48 66.46 66.45 66.30 66.28 66.28 66.27
Cl
$F_5S$
OEt
O
2h
$^{19}F$ NMR (470 MHz, $CDCl_3$)
1.60
2.00 1.21 1.98 1.19
00 95 90 85 80 75 70 65 60 55 50 45
f1 (ppm)

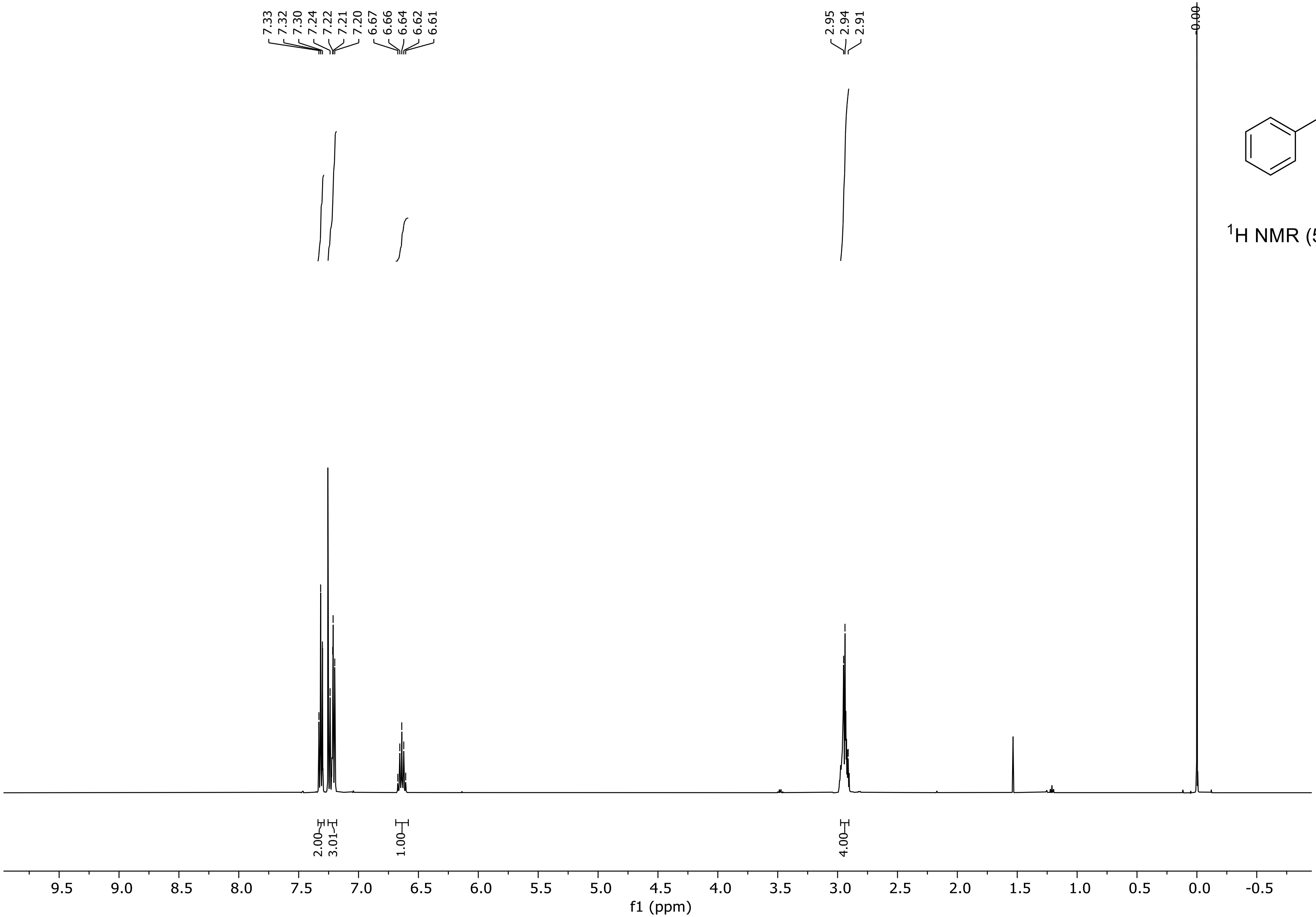


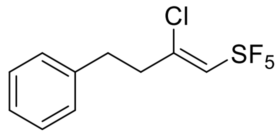


**2i**

$^1$H NMR (500 MHz, $CDCl_3$)

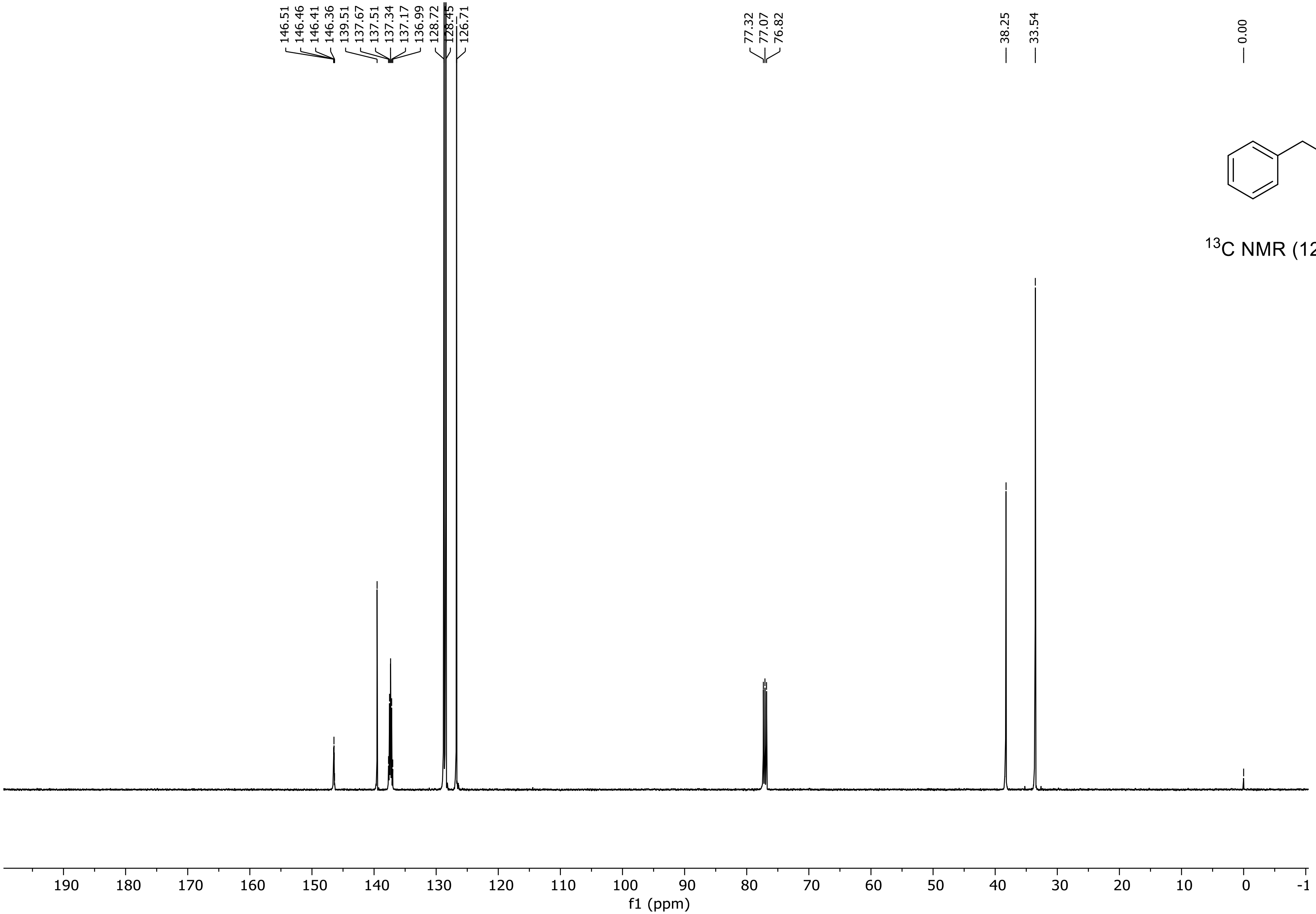


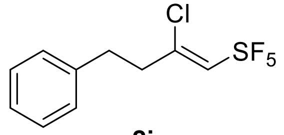


**2i**

$^{13}C$ NMR (126 MHz, $CDCl_3$)

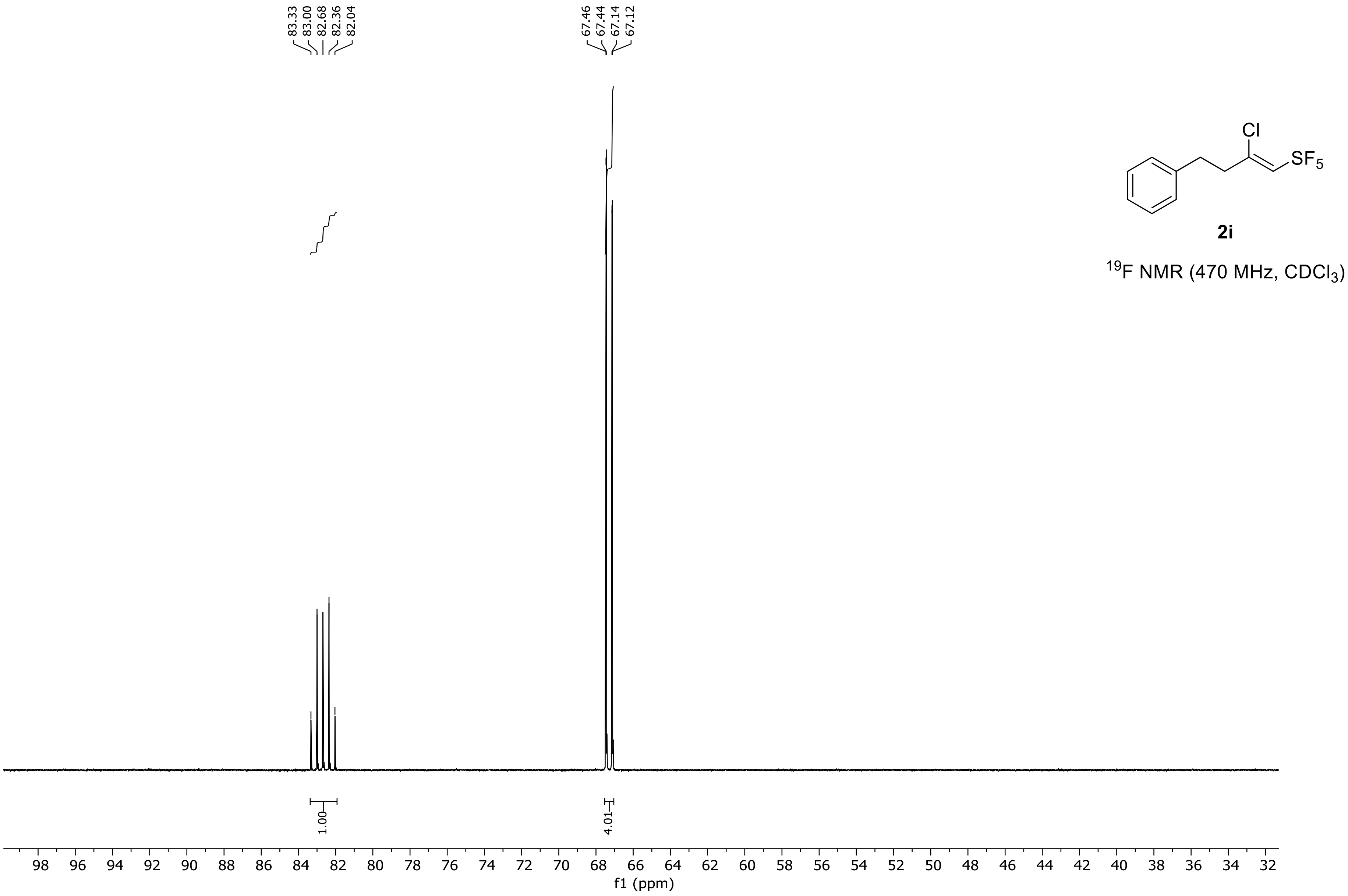
83.33
83.00
82.68
82.36
82.04
67.46
67.44
67.14
67.12
Cl
SF5
2i
19F NMR (470 MHz, CDCl3)
1.00
4.01
98 96 94 92 90 88 86 84 82 80 78 76 74 72 70 68 66 64 62 60 58 56 54 52 50 48 46 44 42 40 38 36 34 32
f1 (ppm)

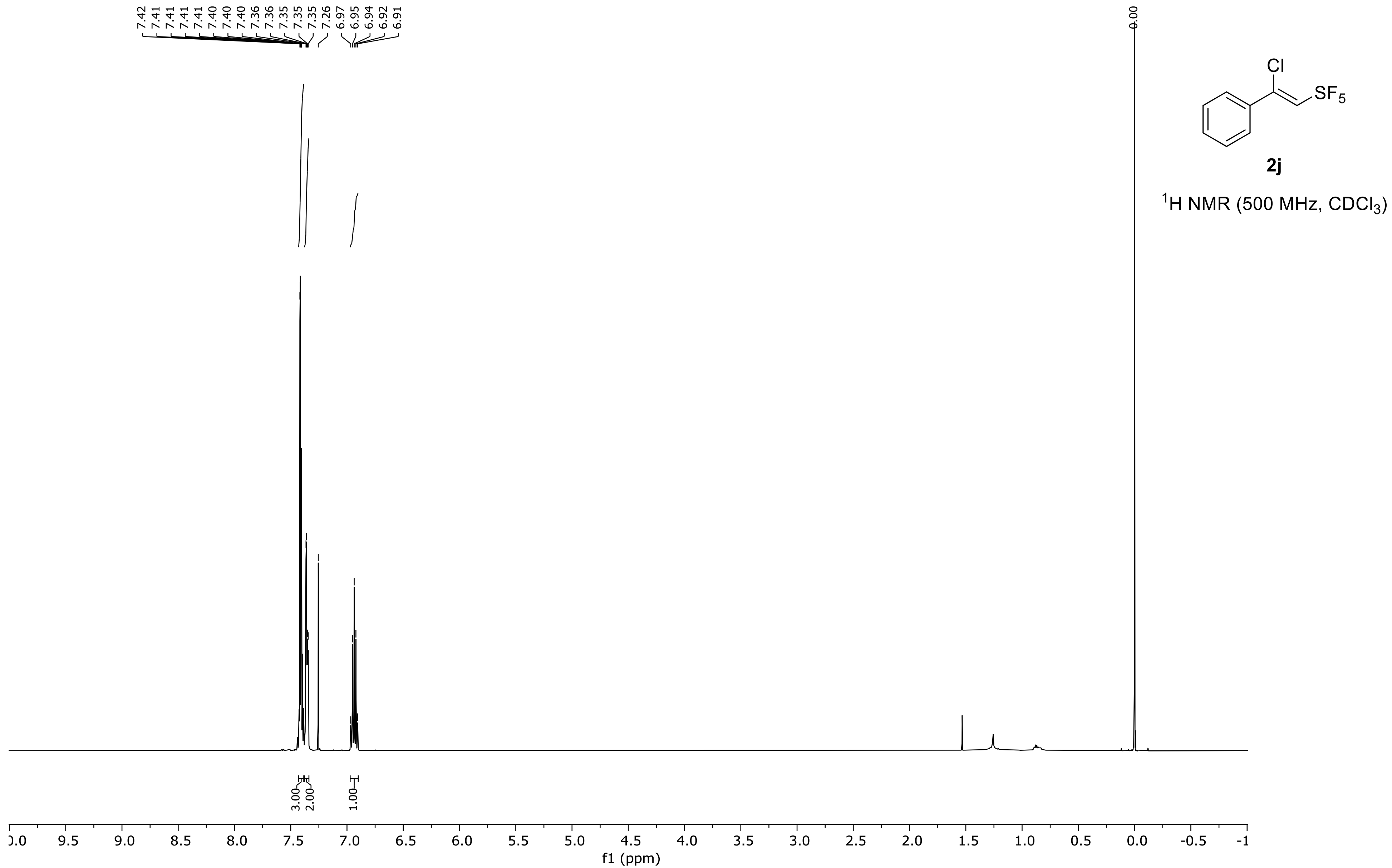
7.42
7.41
7.41
7.41
7.41
7.40
7.40
7.40
7.36
7.36
7.35
7.35
7.35
7.26
6.97
6.95
6.94
6.92
6.91
0.00
3.00
2.00
1.00
f1 (ppm)
Cl
$SF_5$
2j
$^{1}H$ NMR (500 MHz, $CDCl_3$)

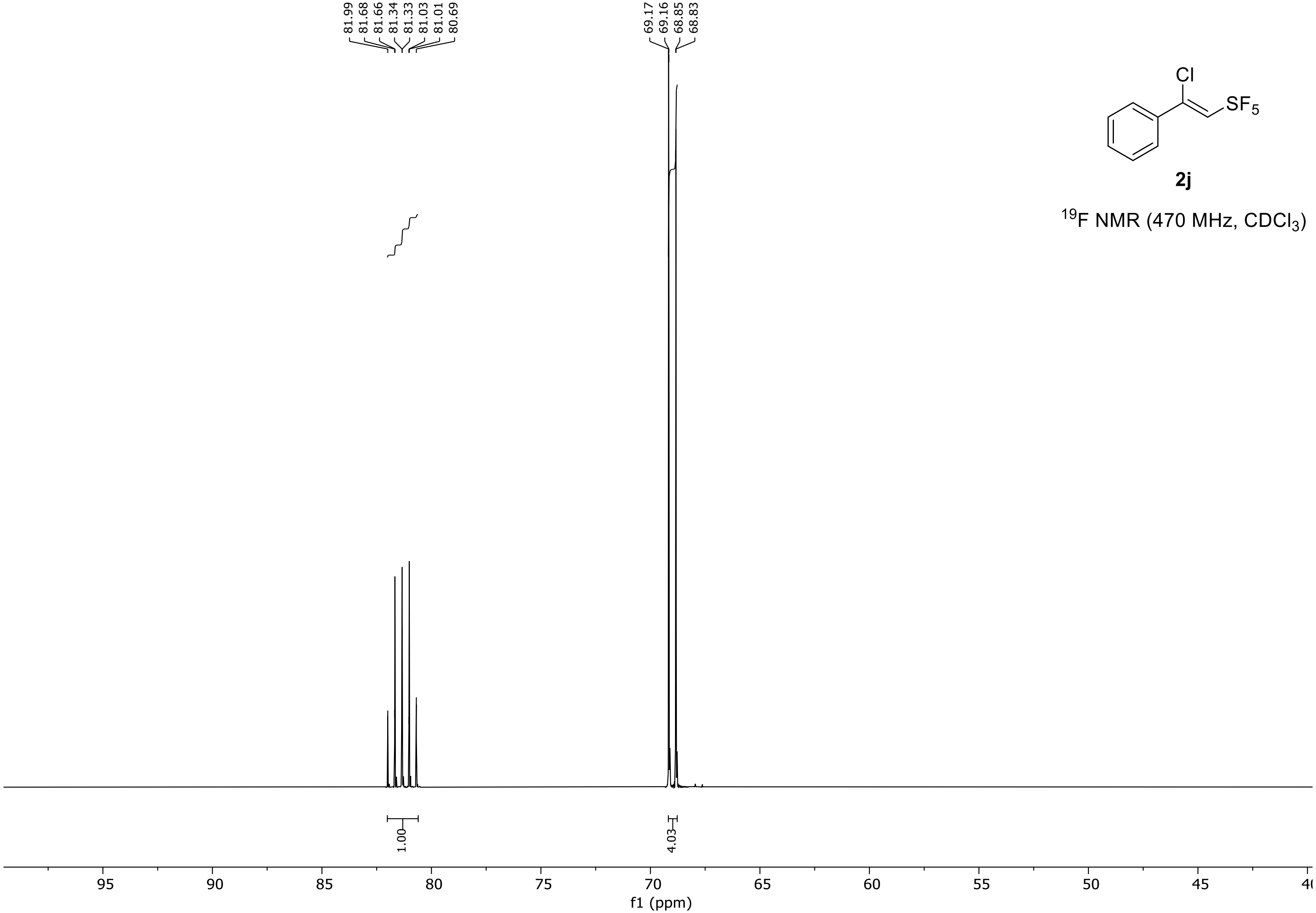


**2j**

$^{19}F$ NMR (470 MHz, $CDCl_3$)

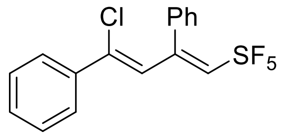


**2k**

$^1$H NMR (500 MHz, $CDCl_3$)

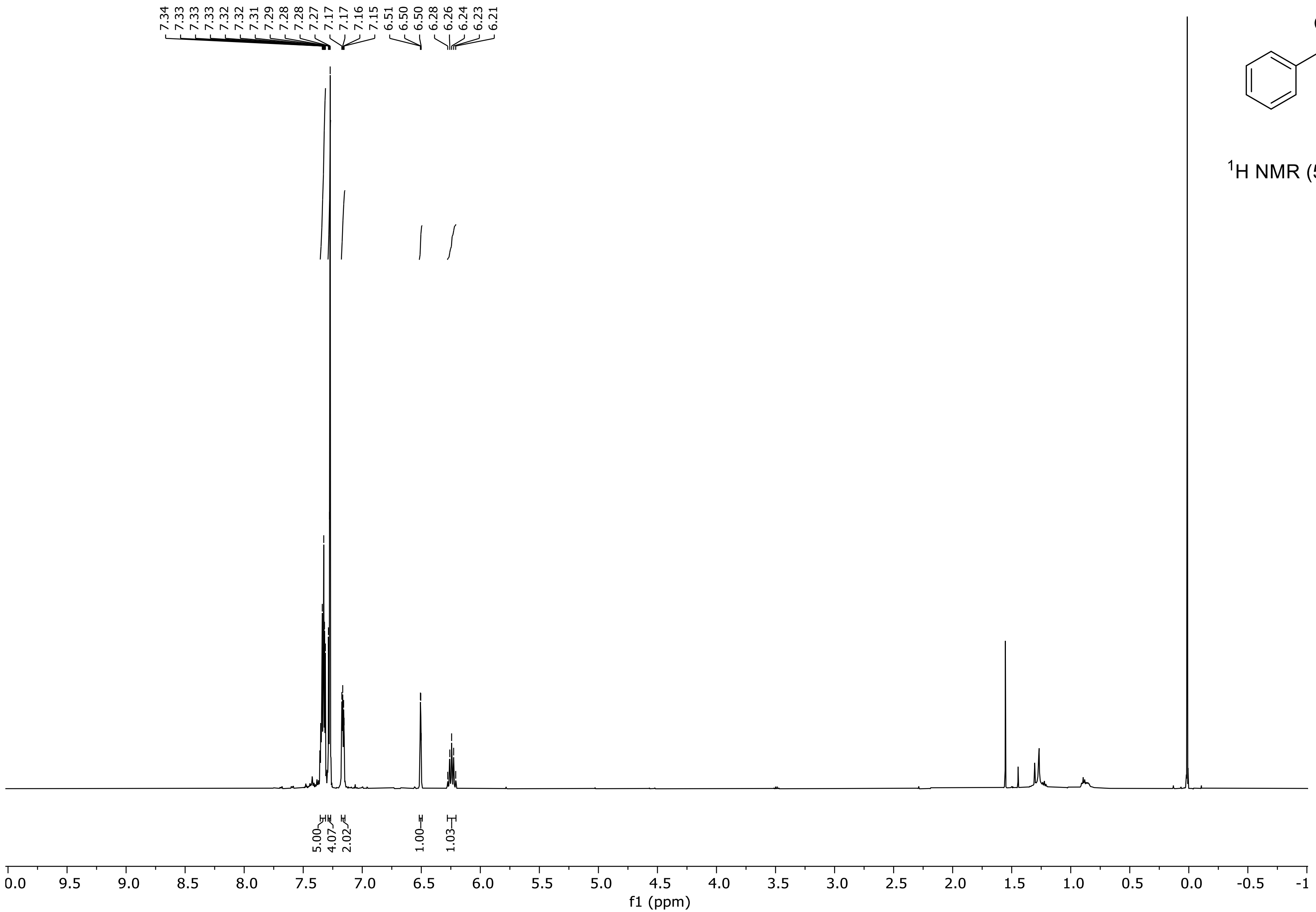

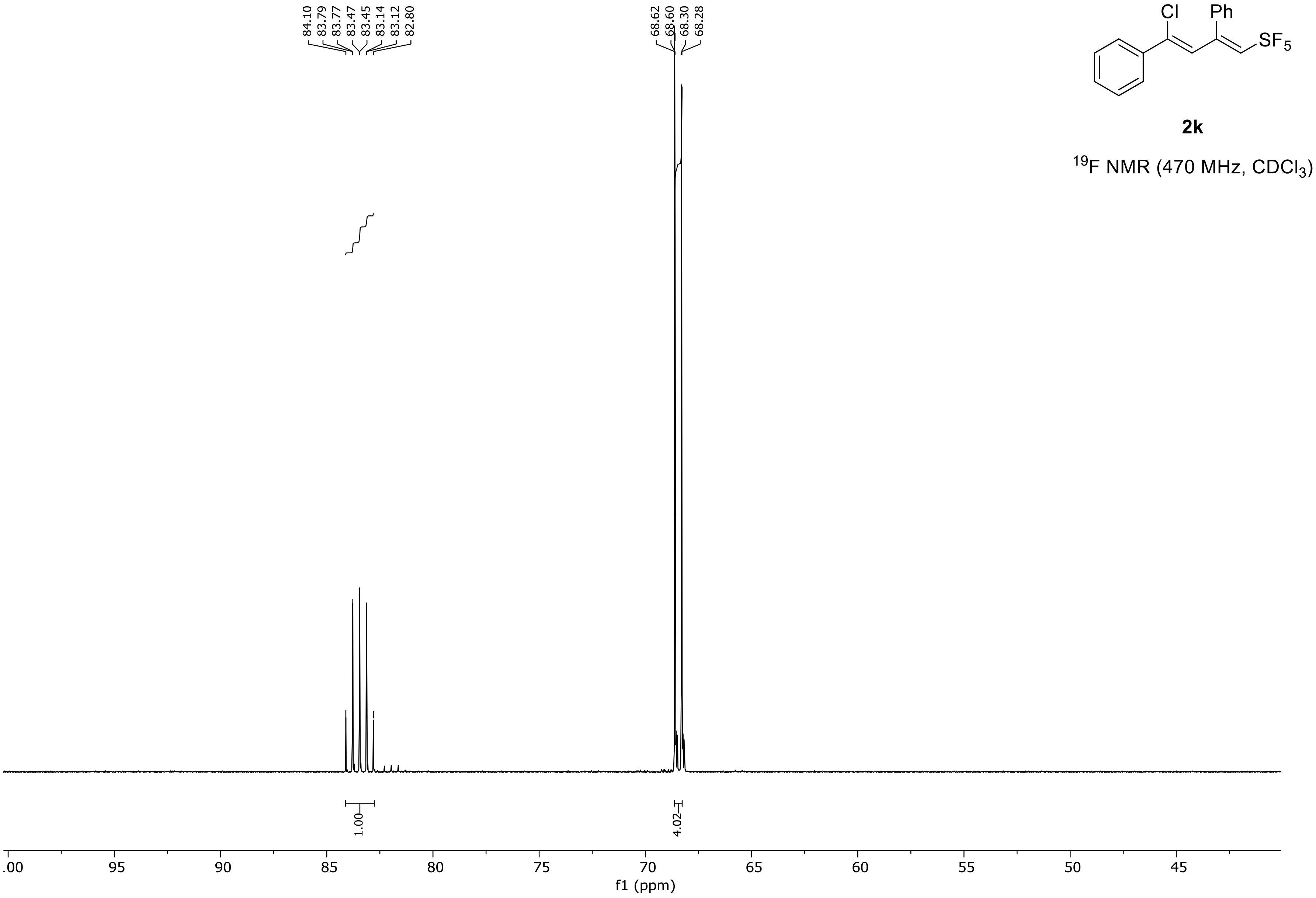


**2k**

$^{19}$F NMR (470 MHz, $CDCl_3$)

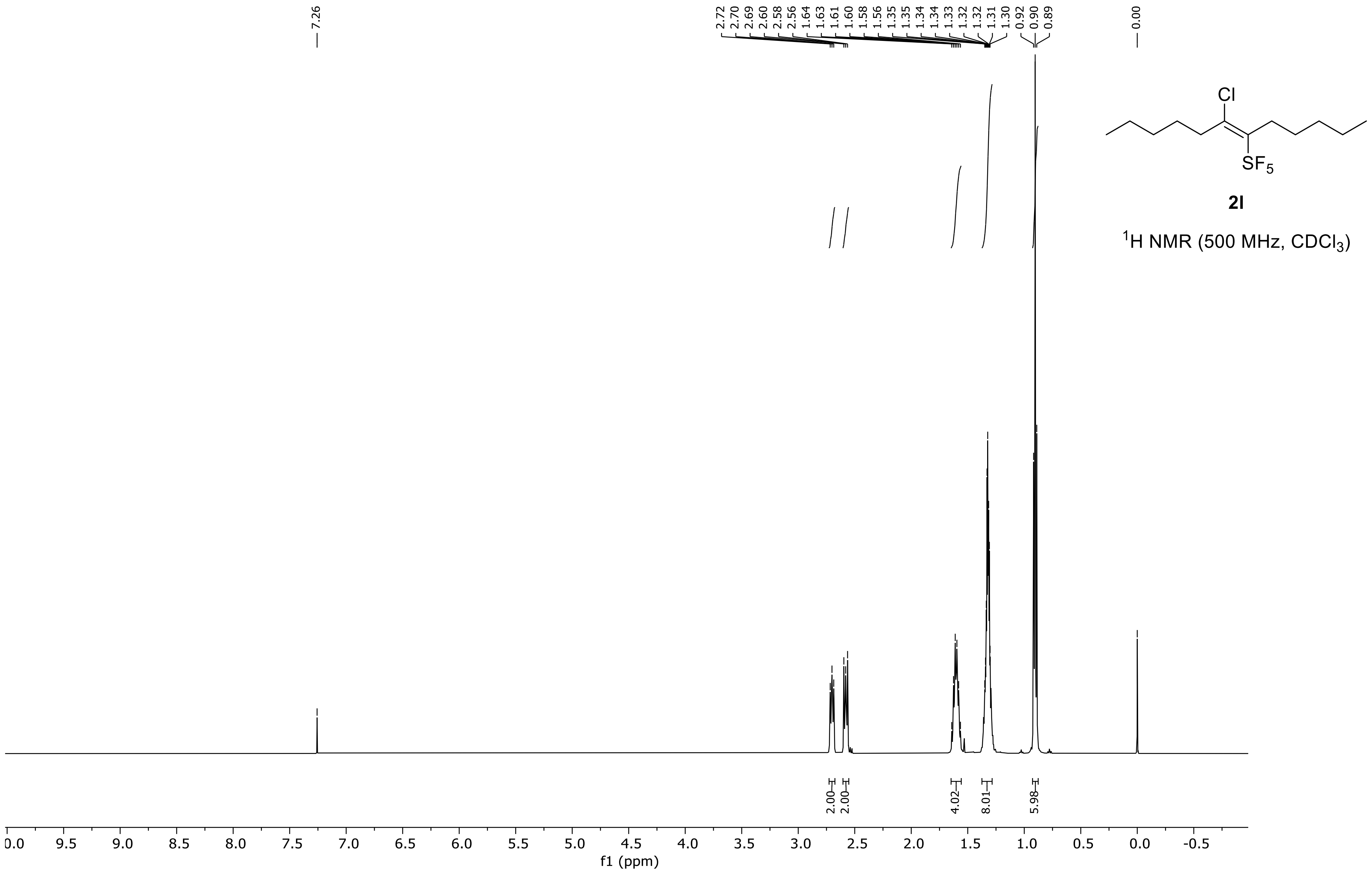


**2l**

$^1H$ NMR (500 MHz, $CDCl_3$)

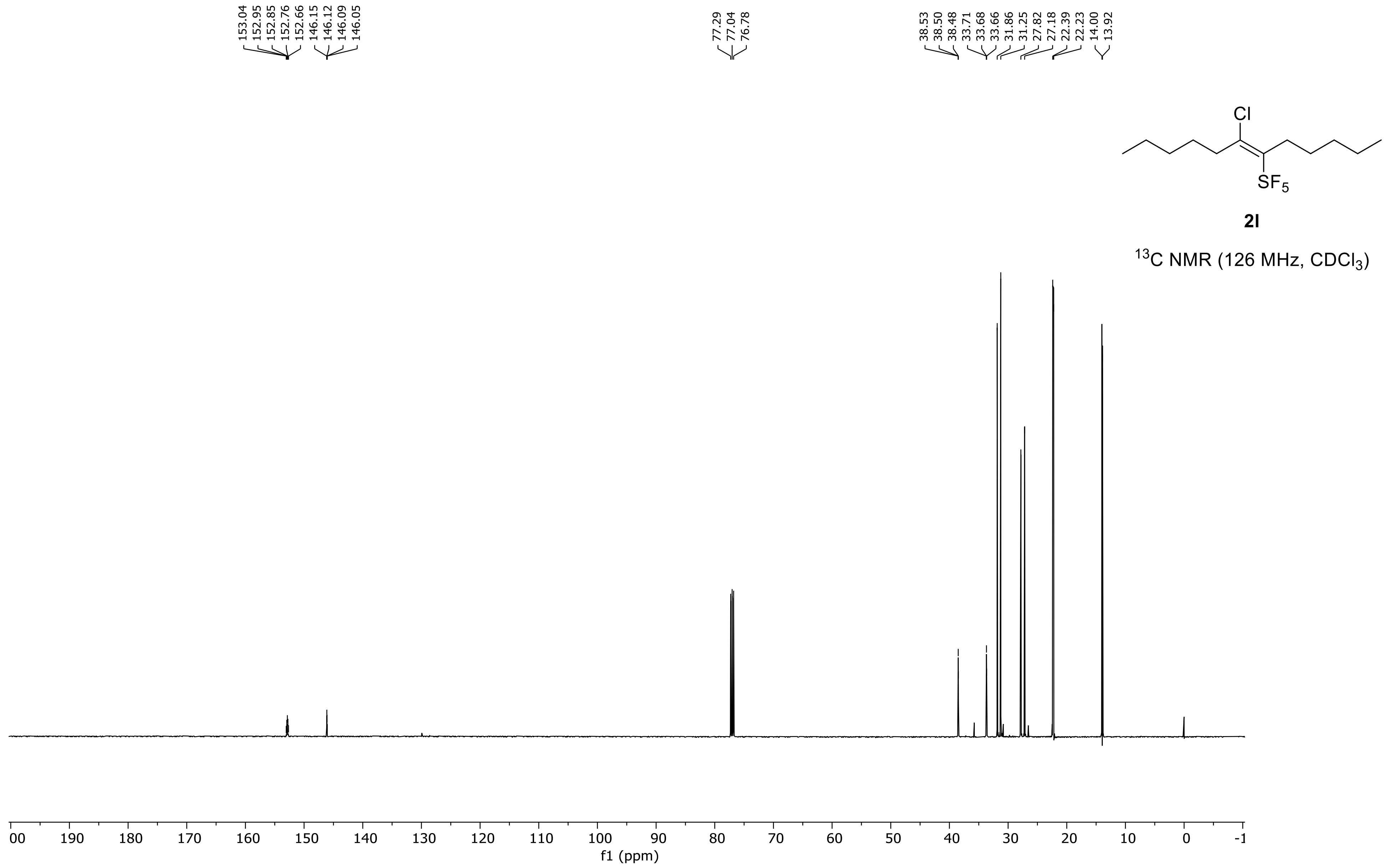


**2l**

$^{13}$C NMR (126 MHz, $CDCl_3$)

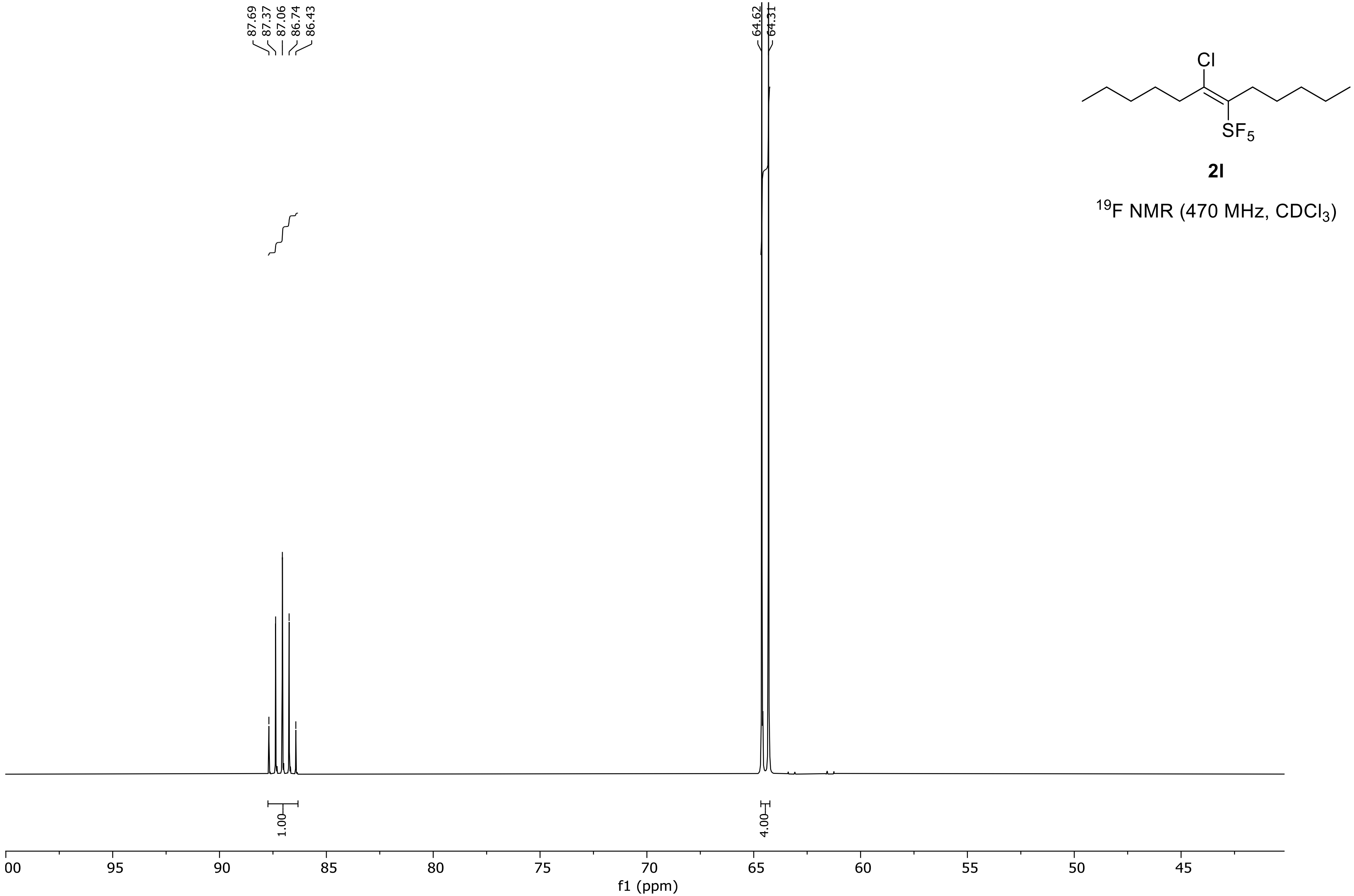


**2l**

$^{19}$F NMR (470 MHz, $CDCl_3$)
