# Supplementary material for: Correction: Amine–borane complex-initiated SF5Cl radical addition on alkenes and alkynes
Source: Beilstein J Org Chem. 2021 Jul 23;17:1725–6. doi: 10.3762/bjoc.17.120 (PMC8313978; doi:10.3762/bjoc.17.120)
Supplement: File 1 — General information, synthetic procedures, additional optimization results, NMR spectra for known compounds (1H, 19F) and full characterization of all new compounds. [file Beilstein_J_Org_Chem-17-1725-s001.pdf]

BEILSTEIN JOURNAL OF ORGANIC CHEMISTRY

Supporting Information

for

Correction: Amine–borane complex-initiated SF_5Cl radical addition on alkenes and alkynes

Audrey Gilbert, Pauline Langowski, Marine Delgado, Laurent Chabaud, Mathieu Pucheault and Jean-François Paquin

Beilstein J. Org. Chem. **2021,** *17,* 1725–1726. doi:10.3762/bjoc.17.120

General information, synthetic procedures, additional optimization results, NMR spectra for known compounds (^{1}H, ^{19}F) and full characterization of all new compounds

License and Terms: This is a supporting information file under the terms of the Creative Commons Attribution License (https://creativecommons.org/licenses/by/4.0). Please note that the reuse, redistribution and reproduction in particular requires that the author(s) and source are credited and that individual graphics may be subject to special legal provisions.
The license is subject to the *Beilstein Journal of Organic Chemistry* terms and conditions: (https://www.beilstein-journals.org/bjoc/terms)

General information S2
Synthesis of dec-9-en-1-yl acetate S3
General procedures for the SF_5Cl additions S3
SF_5Cl additions reactions S4
Full optimization results S13
NMR spectra S17

General information

All reactions were carried out under an argon atmosphere. Et_2O, dichloromethane, THF and toluene were purified using a Vacuum Atmospheres Inc. Solvent Purification System. All other commercially available compounds were used as received. Thin-layer chromatography (TLC) analysis of reaction mixtures was performed using Silicycle silica gel 60 Å F254 TLC plates, and visualized under UV or by staining with potassium permanganate. Flash column chromatography was carried out on Silicycle silica gel 60 Å, 230 – 400 mesh. ^{1}H, ^{13}C and ^{19}F spectra were respectively recorded at 500, 126 and 470 MHz using $CDCl_3$ as the solvent at ambient temperature on an Agilent Technologies 500/54 Premium Shielded spectrometer. The internal standard used was: for ^{1}H NMR tetramethylsilane (δ = 0 ppm); for ^{13}C NMR tetramethylsilane (δ = 0 ppm). For 19F spectra, calibration was performed using a unified scale [1]. Coupling constants (*J*) are measured in hertz (Hz). Multiplicities are reported using the following abbreviations: s = singlet, d = doublet, t = triplet, q = quartet, p = quintet, h = sextet, m = multiplet, br = broad resonance. Low-resolution mass spectra were obtained on a GC/MS using chemical ionization (CI). Infrared spectra were recorded using an ABB MB300 FT-IR spectrometer.

[1] Harris, R. K.; Becker, E. D.; Cabral de Menezes, S. M.; Goodfellow, R.; Granger, P. *Pure Appl. Chem.* **2001**, *73*, 1795–1818.

Synthesis of dec-9-en-1-yl acetate

Dec-9-en-1-yl acetate: Following a procedure described by Schmalz *et al.* [2], dec-9-en-1-ol (1 equiv., 200 mg, 1.28 mmol) and pyridine (10 mL, 0.13 M) were charged in a round-bottom flask at 0 °C. The solution was degassed with argon before a solution of acetic anhydride in pyridine (0.25 M, 1.2 equiv., 1.54 mmol) was added dropwise. The mixture was allowed to warm to room temperature and stirred overnight. The reaction was quenched by the addition of water, before pyridine was evaporated under reduced pressure. The crude mixture was then dissolved in Et_2O, and successively washed with a 10% HCl solution in water and brine. The organic phase was dried over $MgSO_4$, filtered and evaporated under reduce pressure. The crude product was purified by flash chromatography on silica gel using hexane/EtOAc (90:10) as the eluent to yield the title compound as a colorless oil (71%, 179.3 mg). ^{1}H NMR (500 MHz, $CDCl_3$): δ (ppm) = 5.82 (ddt, *J* = 16.9, 10.2, 6.7 Hz, 1H), 5.00 (dq, *J* = 17.1, 1.7 Hz, 1H), 4.94 (ddt, *J* = 10.2, 2.3, 1.2 Hz, 1H), 4.06 (t, *J* = 6.8 Hz, 2H), 2.08 – 2.01 (m, 5H), 1.67 – 1.56 (m, 2H), 1.45 – 1.20 (m, 10H). Analytical data were identical to those previously reported [3].

General procedures for the SF_5Cl additions

Method A: In a microwave vial under inert atmosphere were successively charged the unsaturated compound (1.0 equiv.) and degassed hexanes (0.25 M) at –40 °C. A solution of SF_5Cl in hexanes (1.5 equiv.) was added, followed by a

[2] Hirschhäuser, C.; Velcicky, J.; Schlawe, D.; Hessler, E.; Majdalani, A.; Neudörfl, J.-M.; Prokop, A.; Wieder, T.; Schmalz, H.-G. *Chem. Eur. J.* **2013**, *19*, 13017-13029.

[3] Winter, R.; Nixon, P. G.; Gard, G. L.; Radford, D. H.; Holcomb, N. R.; Grainger, D. W. *J. Fluorine Chem.* **2001**, *107*, 23-30.

solution of Et_3B (1 M in THF, 0.1 equiv.). The mixture was stirred for 3 h at –40 °C. The reaction mixture was then allowed to warm to room temperature, and a saturated solution of $NaHCO_3$ was added to quench the reaction. The phases were separated, and the organic phase was dried over $MgSO_4$. The crude product was concentrated under reduced pressure and purified by flash chromatography on silica gel.

Method B: In a microwave vial under inert atmosphere were successively charged the unsaturated compound (1.0 equiv.) and degassed MTBE (0.33 M) at –40 °C. The vial was hermetically sealed, before a solution of SF_5Cl in hexanes (3 equiv.) was added. A solution of DICAB (0.10 M in MTBE, 0.1 equiv.) was then added and the mixture was stirred for 3 h at 60 °C. The reaction mixture was then allowed to cool down to room temperature, and a saturated solution of $NaHCO_3$ was added to quench the reaction. The phases were separated, and the organic phase was dried over $MgSO_4$. The crude product was concentrated under reduced pressure and purified by flash chromatography on silica gel.

SF_5Cl additions reactions

(2-Chloro-2-phenethoxyethyl)pentafluoro- λ^6-sulfane (2a):

Method A: ((Allyloxy)methyl)benzene (**1**, 100 mg, 0.67 mmol), SF_5Cl (1.37 M in hexanes, 0.74 mL, 1.01 mmol) and Et_3B (1M in THF, 67 µL, 0.067 mmol) were engaged in general procedure, Method A, to afford the title compound **2a** as a colorless oil (185.5 mg, 0.60 mmol, 88%) after purification by flash chromatography using hexanes/EtOAc (95:5) as the eluent. **Method B:** ((Allyloxy)methyl)benzene

(**1**, 100 mg, 0.67 mmol), SF_5Cl (1.32 M in hexanes, 1.53 mL, 2.02 mmol) and DICAB (13.17 mg, 0.067 mmol) were engaged in general procedure, Method B, to afford the title compound **2a** as a colorless oil (161.0 mg, 0.52 mmol, 77%) after purification by flash chromatography using hexanes/EtOAc (95:5) as the eluent. ^{1}H NMR (500 MHz, $CDCl_3$): δ (ppm) = 7.41 – 7.34 (m, 2H), 7.36 – 7.30 (m, 3H), 4.59 (s, 2H), 4.51 – 4.43 (m, 1H), 4.34 – 4.20 (m, 1H), 3.95 – 3.80 (m, 1H), 3.76 (dd, *J* = 10.4, 4.6 Hz, 1H), 3.60 (dd, *J* = 10.4, 6.7 Hz, 1H); ^{19}F NMR (470 MHz, $CDCl_3$): δ (ppm) = 83.9 – 82.4 (m, 1F), 66.6 (dt, *J* = 146.7, 8.0 Hz, 4F). Analytical data were identical to those previously reported [4].

(2-chloro-4-phenylbutyl)pentafluoro-λ^6-sulfane (2b): Method A: 4-Phenyl-1-butene (100 mg, 0.76 mmol), SF_5Cl (1.32 M in hexanes, 0.86 mL, 1.13 mmol) and Et_3B (1M in THF, 76 µL, 0.076 mmol) were engaged in general procedure, Method A, to afford the title compound **2b** as a colorless oil (201.4 mg, 0.68 mmol, 90%) after purification by flash chromatography using 100% hexanes as the eluent. **Method B:** 4-Phenyl-1-butene (100 mg, 0.67 mmol), SF_5Cl (1.37 M in hexanes, 1.66 mL, 2.27 mmol) and DICAB (14.75 mg, 0.076 mmol) were engaged in general procedure, Method B, to afford the title compound **2b** as a colorless oil (191.2 mg, 0.65 mmol, 85%) after purification by flash chromatography using 100% hexanes as the eluent. ^{1}H NMR (500 MHz, $CDCl_3$): δ (ppm) = 7.32 (t, *J* = 7.3 Hz, 2H), 7.27 – 7.17 (m, 3H), 4.34 – 4.26 (m, 1H), 4.03 (td, *J* = 14.4, 8.4 Hz, 1H), 3.91 (td, *J* = 14.3, 7.9 Hz, 1H), 2.98 – 2.89 (m, 1H), 2.83 – 2.74 (m, 1H), 2.32 – 2.21 (m, 1H),

[4] Gilbert, A.; Paquin, J.-F. *J. Fluorine Chem.* **2019**, *221*, 70-74.

2.04 (td, J = 14.2, 9.3 Hz, 1H); ^{19}F NMR (470 MHz, $CDCl_3$): δ (ppm) = 83.9 – 82.4 (m, 1F), 66.5 (dt, J = 146.6, 8.2 Hz, 4F). Analytical data were identical to those previously reported [5].

(2-chloro-2-phenylethyl)pentafluoro-λ^6-sulfane (2c): Method A: Styrene (100 mg, 0.38 mmol), SF_5Cl (1.32 M in hexanes, 0.43 mL, 0.56 mmol) and Et_3B (1M in THF, 38 µL, 0.038 mmol) were engaged in general procedure, Method A, to afford the title compound **2c** which could not be isolated. The yield was estimated using NMR analysis and 2-fluoro-4-nitrotoluene as the reference; yield: 8%.

(4-chloro-2,4-diphenylbutyl)pentafluoro-λ^6-sulfane (2d): Method B: Styrene (200 mg, 1.92 mmol), SF_5Cl (1.37 M in hexanes, 4.21 mL, 5.76 mmol) and DICAB (37.5 mg, 0.19 mmol) were engaged in general procedure, Method B, to afford the title compound **2d** as a colorless oil (113.0 mg, 0.30 mmol, 15%) after purification by flash chromatography using 100% hexane as the eluent. ^{1}H NMR (500 MHz, $CDCl_3$): δ (ppm) = 7.44 – 7.39 (m, 10H), 5.39 (t, J = 6.8 Hz, 1H), 5.03 (dd, J = 7.9, 6.6 Hz, 1H), 4.37 – 4.27 (m, 2H), 4.02 (dd, J = 11.3, 6.6 Hz, 1H), 3.95 (dd, J = 11.4, 7.9 Hz, 1H); ^{13}C NMR (126 MHz, $CDCl_3$): δ (ppm) = 138.5, 138.0, 129.4, 129.2, 129.1, 128.8, 127.4, 126.9, 76.9 (p, J = 13.3 Hz), 61.8, 56.4 (p, J = 4.7 Hz), 48.4; ^{19}F NMR (470 MHz, $CDCl_3$): δ (ppm) = 83.2 – 80.8 (m, 1F), 66.4 (dt, J = 147.1, 7.8 Hz, 4F); GC-MS (CI): m/z calcd for $C_{16}H_{15}ClF_5S$ $[M-H]^+$ 369.05 found 369.07 (under all conditions tested for high-resolution mass spectra [ESI (+), ESI (–),

[5] Ponomarenko, M. V.; Serguchev, Y. A.; Röschenthaler, G.-V. *Synthesis* **2010**, *22*, 3906-3912.

APPI], no significant ion was detected); IR (ATR, Diamond): ν (cm^{-1}) = 3032, 1495, 1456, 1310, 1198, 878, 824, 696.

9-chloro-10-(pentafluoro-λ⁶-sulfanyl)decan-1-ol (2e): Method A: Dec-9-en-1-ol (50 mg, 0.32 mmol), SF_5Cl (0.96 M in hexanes, 0.50 mL, 0.48 mmol) and Et_3B (1 M in THF, 32 µL, 0.032 mmol) were engaged in general procedure, Method A, to afford the title compound **2e** as a colorless oil (15.1 mg, 0.047 mmol, 15%) after purification by flash chromatography using hexanes/EtOAc (95:5) as the eluent. **Method B:** Dec-9-en-1-ol (**x,** 50 mg, 0.32 mmol), SF_5Cl (0.96 M in hexanes, 0.99 mL, 0.96 mmol) and DICAB (6.2 mg, 0.032 mmol) were engaged in general procedure, Method B, to afford the title compound **2e** which could not be isolated. The yield was estimated using NMR analysis and 2-fluoro-4-nitrotoluene as the reference; yield: 43%. ^{1}H NMR (500 MHz, $CDCl_3$): δ (ppm) = 4.40 – 4.30 (m, 1H), 4.06 – 3.96 (m, 1H), 3.95 – 3.85 (m, 1H), 3.40 (t, *J* = 6.7 Hz, 2H), 1.97 – 1.87 (m, 1H), 1.82 – 1.67 (m, 1H), 1.67 – 1.25 (m, 13H); ^{19}F NMR (470 MHz, $CDCl_3$): δ (ppm) = 88.17 – 78.75 (m), 66.19 (dt, *J* = 146.5, 8.3 Hz). Analytical data were identical to those previously reported [3, 6].

9-chloro-10-(pentafluoro- λ⁶-sulfanyl)decyl acetate (2f): Method A: Dec-9-en-1-yl acetate (50 mg, 0.25 mmol), SF_5Cl (0.96 M in hexanes, 0.39 mL, 0.38 mmol) and Et_3B (1 M in THF, 25 µL, 0.032 mmol) were engaged in general procedure,

[6] Dolbier Jr., W. R.; Aït-Mohand, S.; Schertz, T. D.; Sergeeva, T. A.; Cradlebaugh, J. A.; Mitani, A.; Gard, G. L.; Winter, R. W.; Thrasher, J. S. *J. Fluorine Chem.* **2006**, *127*, 1302-1310.

Method A, to afford the title compound **2f** as a colorless oil (84.0 mg, 0.23 mmol, 92%) after purification by flash chromatography using hexanes/EtOAc (95:5) as the eluent. **Method B:** Dec-9-en-1-yl acetate (33.7 mg, 0.17 mmol), SF_5Cl (0.96 M in hexanes, 0.53 mL, 0.51 mmol) and DICAB (3.3 mg, 0.017 mmol) were engaged in general procedure, Method B, to afford the title compound **2f** which could not be isolated. The yield was estimated using NMR analysis and 2-fluoro-4-nitrotoluene as the reference; yield: 3%. ^{1}H NMR (500 MHz, $CDCl_3$): δ (ppm) = 4.39 – 4.27 (m, 1H), 4.05 (t, *J* = 6.7 Hz, 2H), 4.03 – 3.96 (m, 1H), 3.95 – 3.85 (m, 1H), 2.05 (s, 3H), 1.98 – 1.84 (m, 1H), 1.80 – 1.68 (m, 1H), 1.65 – 1.53 (m, 3H), 1.50 – 1.42 (m, 1H), 1.38 – 1.28 (m, 8H); ^{19}F NMR (470 MHz, $CDCl_3$): δ (ppm) = 84.29 – 81.36 (m, 1F), 66.16 (dt, *J* = 146.3, 8.2 Hz, 4F). Analytical data were identical to those previously reported [3, 6].

1-chloro-2-(pentafluoro-λ^6-sulfanyl)ethyl benzoate (2g): Method A: Vinyl benzoate (100 mg, 0.68 mmol), SF_5Cl (1.32 M in hexanes, 0.77 mL, 1.01 mmol) and Et_3B (1M in THF, 68 µL, 0.068 mmol) were engaged in general procedure, Method A, to afford the title compound **2g** as a colorless oil (156.7 mg, 0.50 mmol, 75%) after purification by flash chromatography using hexanes/Et_2O (98:2) as the eluent. **Method B:** Vinyl benzoate (100 mg, 0.68 mmol), SF_5Cl (1.32 M in hexanes, 1.53 mL, 2.03 mmol) and DICAB (13.17 mg, 0.068 mmol) were engaged in general procedure, Method B, to afford the title compound **2g** as a colorless oil (175.5 mg, 0.57 mmol, 84%) after purification by flash chromatography using hexanes/Et_2O (98:2) as the eluent. ^{1}H NMR (500 MHz, $CDCl_3$): δ (ppm) = 8.10 – 8.05 (m, 2H),

7.69 – 7.62 (m, 1H), 7.53 – 7.46 (m, 2H), 7.14 (dd, *J* = 9.8, 2.1 Hz, 1H), 4.49 – 4.37 (m, 1H), 4.26 – 4.16 (m, 1H); ^{13}C NMR (126 MHz, $CDCl_3$): δ (ppm) = 163.4, 134.5, 130.2, 128.8, 127.9, 77.6 (p, *J* = 5.4 Hz), 73.4 (p, *J* = 14.7 Hz); ^{19}F NMR (470 MHz, $CDCl_3$): δ (ppm) = 81.5 – 80.1 (m, 1F), 66.8 (dt, *J* = 147.2, 8.1 Hz, 4F); GC-MS (CI): m/z calcd for $C_9H_9ClF_5O_2S$ $[M+H]^+$ 310.99 found 311.00 (under all conditions tested for high-resolution mass spectra [ESI (+), ESI (–), APPI], no significant ion was detected); IR (ATR, Diamond): ν (cm^{-1}) = 3040, 1744, 1603, 1452, 1244, 1103, 1061, 837.

Ethyl 4-chloro-2-methyl-5-(pentafluoro-λ^6-sulfanyl)pentanoate (2h): Method A: Ethyl 2-methylpent-4-enoate (50 mg, 0.35 mmol), SF_5Cl (0.96 M in hexanes, 0.55 mL, 0.52 mmol) and Et_3B (1 M in THF, 35 µL, 0.035 mmol) were engaged in general procedure, Method A, to afford the title compound **2h** as a colorless oil (86.8 mg, 0.28 mmol, 81%) in a 63:37 mixture of diastereoisomers after purification by flash chromatography using hexanes/EtOAc (90:10) as the eluent. **Method B:** Ethyl 2-methylpent-4-enoate (50 mg, 0.35 mmol), SF_5Cl (0.96 M in hexanes, 1.10 mL, 1.05 mmol) and DICAB (6.9 mg, 0.035 mmol) were engaged in general procedure, Method B, to afford the title compound **2h** as a colorless oil (74.7 mg, 0.25 mmol, 70%) in a 57:43 mixture of diastereoisomers after purification by flash chromatography using hexanes/EtOAc (95:5) as the eluent. ^{1}H NMR (500 MHz, $CDCl_3$): δ (ppm) = 4.50 – 4.44 (m, 1H), 4.42 – 4.35 (m, 0.6H), 4.17 (app. p, *J* = 7.1 Hz, 3.2H), 4.07 – 3.97 (m, 1.6H), 3.96 – 3.82 (m, 1.6H), 2.90 – 2.81 (m, 1H), 2.81 – 2.71 (m, 0.6H), 2.35 (ddd, *J* = 13.8, 10.7, 2.7 Hz, 1H), 2.18 (ddd, *J* = 15.4, 10.5,

5.1 Hz, 0.6H), 2.01 (ddd, *J* = 14.4, 8.9, 3.5 Hz, 0.6H), 1.68 (ddd, *J* = 14.3, 11.0, 3.2 Hz, 1H), 1.31 – 1.24 (m, 4.8H), 1.24 (d, *J* = 7.2 Hz, 3H), 1.22 (d, *J* = 7.0 Hz, 1.8H); ^{19}F NMR (470 MHz, $CDCl_3$): δ (ppm) = 83.6 – 82.0 (m, 1.6F), 66.6 (dt, *J* = 146.6, 8.1 Hz, 4F), 66.4 (dt, *J* = 146.5, 8.3 Hz, 2.4F). Analytical data were identical to those previously reported [7].

(*E*)-(2-chloro-4-phenylbut-1-en-1-yl)pentafluoro-λ^6-sulfane (2i): Method A: 4-Phenyl-1-butyne (50 mg, 0.38 mmol), SF_5Cl (0.96 M in hexanes, 0.60 mL, 0.58 mmol) and Et_3B (1M in THF, 38 µL, 0.038 mmol) were engaged in general procedure, Method A, to afford the title compound **2i** as a colorless oil (89 mg, 0.30 mmol, 79%) after purification by flash chromatography using 100% hexanes as the eluent. **Method B:** 4-Phenyl-1-butyne (100 mg, 0.77 mmol), SF_5Cl (1.37 M in hexanes, 1.68 mL, 2.30 mmol) and DICAB (14.99 mg, 0.077 mmol) were engaged in general procedure, Method B, to afford the title compound **2i** as a colorless oil (196.8 mg, 0.67 mmol, 88%) after purification by flash chromatography using 100% hexanes as the eluent. ^{1}H NMR (500 MHz, $CDCl_3$): δ (ppm) = 7.35 – 7.28 (m, 2H), 7.26 – 7.17 (m, 3H), 6.64 (p, *J* = 8.2 Hz, 1H), 3.00 – 2.89 (m, 4H); ^{13}C NMR (126 MHz, $CDCl_3$): δ (ppm) = 146.4 (p, *J* = 6.4 Hz), 139.5, 137.3 (p, *J* = 21.8 Hz), 128.7, 128.5, 126.7, 38.3, 33.5; ^{19}F NMR (470 MHz, $CDCl_3$): δ (ppm) = 83.4 – 82.0 (m, 1F), 67.3 (dd, *J* = 151.4, 8.3 Hz, 4F); GC-MS (CI): m/z calcd for $C_{10}H_9ClF_5S$ $[M-H]^+$ 291.00 found 291.00 (under all conditions tested for high-resolution mass

[7] Lim, D. S.; Ngo, S. C.; Lal, S. G.; Minnich, K. E.; Welch, J. T. *Tetrahedron Lett.* **2008**, *49*, 5662-5663.

spectra [ESI (+), ESI (–), APPI], no significant ion was detected); IR (ATR, Diamond): ν (cm^{-1}) = 3090, 3030, 1639, 1456, 1178, 1040, 989, 833.

(*E*)-(2-chloro-2-phenylvinyl)pentafluoro-λ^6-sulfane (2j): Method A: Ethynylbenzene (50 mg, 0.49 mmol), SF_5Cl (0.96 M in hexanes, 0.76 mL, 0.73 mmol) and Et_3B (1M in THF, 49 µL, 0.049 mmol) were engaged in general procedure, Method A, to afford the title compound **2j** as a colorless oil (23.6 mg, 0.089 mmol, 18%) after purification by flash chromatography using 100% pentane as the eluent. **Method B:** Ethynylbenzene (50 mg, 0.49 mmol), SF_5Cl (0.96 M in hexanes, 1.53 mL, 1.47 mmol) and DICAB (9.6 mg, 0.049 mmol) were engaged in general procedure, Method B, to afford the title compound **2j** as a colorless oil (30.2 mg, 0.11 mmol, 23%) after purification by flash chromatography using 100% pentane as the eluent. ^{1}H NMR (500 MHz, $CDCl_3$): δ (ppm) = 7.44 – 7.38 (m, 3H), 7.38 – 7.33 (m, 2H), 6.94 (p, *J* = 7.7 Hz, 1H); ^{19}F NMR (470 MHz, $CDCl_3$): δ (ppm) = 82.22 – 80.33 (m, 1F), 69.00 (dd, *J* = 152.8, 7.5 Hz, 4F). Analytical data were identical to those previously reported [6,8].

((1*Z*,3*E*)-4-chloro-2,4-diphenylbuta-1,3-dien-1-yl)pentafluoro-λ^6-sulfane (2k): Method B: Ethynylbenzene (50 mg, 0.49 mmol), SF_5Cl (0.96 M in hexanes, 1.53 mL, 1.47 mmol) and DICAB (9.6 mg, 0.049 mmol) were engaged in general procedure, Method B, to afford the title compound **2k** as a colorless oil (9.1 mg, 0.025 mmol, 5%) after purification by flash chromatography using 100% pentane

[8] Aït-Mohand, S.; Dolbier Jr, W. R. *Org. Lett.* **2002**, *4*, 3013-3015.

as the eluent. ^{1}H NMR (500 MHz, $CDCl_3$): δ (ppm) = 7.36 – 7.30 (m, 5H), 7.30 – 7.26 (m, 3H), 7.18 – 7.13 (m, 2H), 6.54 – 6.48 (m, 1H), 6.24 (p, *J* = 8.7 Hz, 1H); ^{19}F NMR (470 MHz, $CDCl_3$): δ (ppm) = 84.44 – 82.31 (m, 1F), 68.45 (dd, *J* = 152.4, 8.6 Hz, 4F). Analytical data were identical to those previously reported. [6,8]

(*E*)-(7-chlorododec-6-en-6-yl)pentafluoro-λ^6-sulfane (2l): Method A: Dodec-6-yne (50 mg, 0.30 mmol), SF_5Cl (0.96 M in hexanes, 0.47 mL, 0.46 mmol) and Et_3B (1M in THF, 30 µL, 0.030 mmol) were engaged in general procedure, Method A, to afford the title compound **2l** as a colorless oil (63.3 mg, 0.19 mmol, 65%) after purification by flash chromatography using 100% hexanes as the eluent. **Method B:** Dodec-6-yne (50 mg, 0.30 mmol), SF_5Cl (0.96 M in hexanes, 0.94 mL, 0.90 mmol) and DICAB (5.9 mg, 0.030 mmol) were engaged in general procedure, Method B, to afford the title compound **2l** which could not be isolated. The yield was estimated using NMR analysis and 2-fluoro-4-nitrotoluene as the reference; yield: 17%. ^{1}H NMR (500 MHz, $CDCl_3$): δ (ppm) = 2.73 – 2.66 (m, 2H), 2.62 – 2.51 (m, 2H), 1.66 – 1.55 (m, 4H), 1.40 – 1.18 (m, 8H), 0.90 (t, *J* = 7.0 Hz, 6H); ^{13}C NMR (126 MHz, $CDCl_3$): δ (ppm) =152.9 (p, *J* = 12.0 Hz), 146.1 (p, *J* = 3.9 Hz), 38.5 (t, *J* = 3.2 Hz), 33.7 (t, *J* = 3.1 Hz), 31.9, 31.3, 27.8, 27.2, 22.4, 22.2, 14.0, 13.9; ^{19}F NMR (470 MHz, $CDCl_3$): δ (ppm) = 87.1 (p, *J* = 148.1 Hz, 1F), 64.5 (d, *J* = 148.1 Hz, 4F); GC-MS (CI): m/z calcd for $C_{12}H_{22}ClF_5S$ $[M]^+$ 328.11 found 328.00 (under all conditions tested for high-resolution mass spectra [ESI (+), ESI (–), APPI], no significant ion was detected); IR (ATR, Diamond): ν (cm^{-1}) = 2959, 2864, 1618, 1460, 1040, 824, 766, 648.

Full optimization results

1. Evaluation of the amino-borane complexes

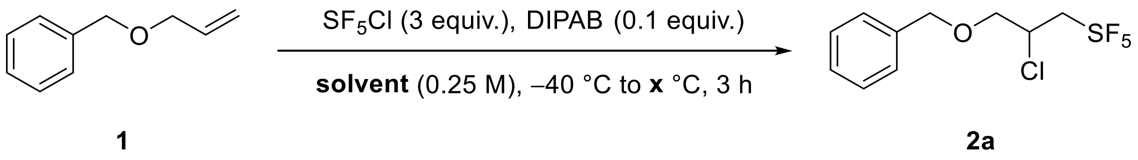

Entry	Solvent	x (°C)	Conversion (%)[a]	Yield (%)[b]
1	hexane	30	28	2
2	hexane	40	32	6
3	hexane	50	33	7
4	hexane	60	55	5
5	EtOAc	30	100	72
6	EtOAc	40	100	64
7	EtOAc	50	100	5
8	EtOAc	60	100	29
9	MTBE	30	77	40
10	MTBE	40	69	6
11	MTBE	50	79	41
12	MTBE	60	92	26
13	THF	30	100	28
14	THF	40	100	33
15	THF	50	100	28
16	THF	60	100	30
17	toluene	30	39	14
18	toluene	40	41	5
19	toluene	50	46	8
20	toluene	60	83	41
21	MeOH	30	100	0
22	MeOH	40	100	traces
23	MeOH	50	100	traces
24	MeOH	60	100	0
25	acetone	30	100	traces
26	acetone	40	100	0
27	acetone	50	100	9
28	CH_2Cl_2	30	15	traces

[a]Disappearance of the starting material, estimated by ^{1}H NMR analysis of the crude mixture using 2-fluoro-4-nitrotoluene as an internal standard. [b]Yield estimated by ^{19}F NMR analysis of the crude mixture using 2-fluoro-4-nitrotoluene as an internal standard.

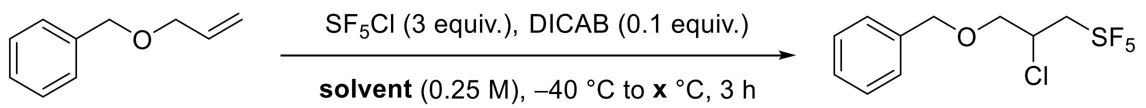

1 **2a**

Entry	Solvent	x (°C)	Conversion (%)[a]	Yield (%)[b]
1	hexane	30	25	1
2	hexane	40	17	2
3	hexane	50	82	72
4	hexane	60	75	32
5	EtOAc	30	100	traces
6	EtOAc	40	100	4
7	EtOAc	50	100	62
8	EtOAc	60	100	56
9	MTBE	30	49	3
10	MTBE	40	100	65
11	MTBE	50	94	21
12	MTBE	60	100	86
13	THF	30	100	43
14	THF	40	100	27
15	THF	50	100	38
16	THF	60	100	20
17	toluene	30	58	37
18	toluene	40	53	39
19	toluene	50	50	23
20	toluene	60	71	33
21	MeOH	30	100	traces
22	MeOH	40	100	traces
23	MeOH	50	100	traces
24	MeOH	60	100	traces
25	acetone	30	100	1
26	acetone	40	100	0
27	acetone	50	100	traces
28	CH_2Cl_2	30	24	traces

[a]Disappearance of the starting material, estimated by ^{1}H NMR analysis of the crude mixture using 2-fluoro-4-nitrotoluene as an internal standard. [b]Yield estimated by ^{19}F NMR analysis of the crude mixture using 2-fluoro-4-nitrotoluene as an internal standard.

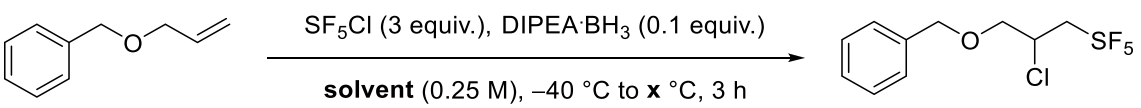

1 **2a**

Entry	solvent	x (°C)	Conversion (%)[a]	Yield (%)[b]
1	hexane	30	32	traces
2	hexane	40	41	8
3	hexane	50	47	10
4	hexane	60	58	10
5	EtOAc	30	100	18
6	EtOAc	40	100	58
7	EtOAc	50	100	8
8	EtOAc	60	100	4
9	THF	30	100	6
10	THF	40	100	19
11	THF	50	100	24
12	THF	60	100	32
13	toluene	30	71	23
14	toluene	40	78	44
15	toluene	50	53	10
16	toluene	60	53	1

[a]Disappearance of the starting material, estimated by ^{1}H NMR analysis of the crude mixture using 2-fluoro-4-nitrotoluene as an internal standard. [b]Yield estimated by ^{19}F NMR analysis of the crude mixture using 2-fluoro-4-nitrotoluene as an internal standard.

2. Evaluation of the increase in the amount of DICAB and the reaction time

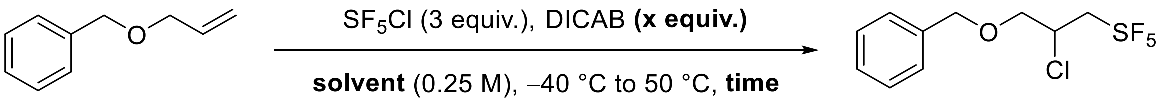

1 **2a**

Solvent	DICAB equiv.	Time (h)	Conversion (%)[a]	Yield (%)[b]
EtOAc	0.2	3	100	80
EtOAc	0.1	6	100	86
hexane	0.2	3	61	37
hexane	0.1	6	94	89
hexane	0	6	20	traces

[a]Disappearance of the starting material, estimated by ^{1}H NMR analysis of the crude mixture using 2-fluoro-4-nitrotoluene as an internal standard. [b]Yield estimated by ^{19}F NMR analysis of the crude mixture using 2-fluoro-4-nitrotoluene as an internal standard.

3. Evaluation of the decrease in the amount of amino-borane complexe

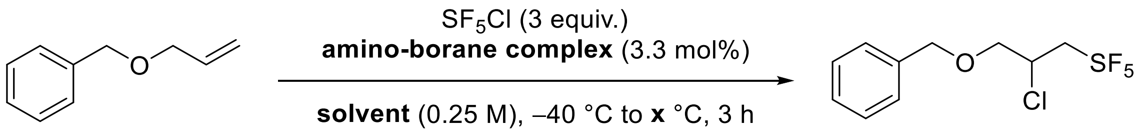

1 **2a**

Entry	solvent	amino-borane complex	x (°C)	Conversion (%)[a]	Yield (%)[b]
1	hexane	DIPAB	30	17	traces
2	hexane	DIPAB	40	19	traces
3	hexane	DIPAB	50	25	2
4	hexane	DIPAB	60	65	41
5	hexane	DICAB	30	14	traces
6	hexane	DICAB	40	17	2
7	hexane	DICAB	50	93	86
8	hexane	DICAB	60	30	22
9	EtOAc	DIPAB	30	21	traces
10	EtOAc	DIPAB	40	70	traces
11	EtOAc	DIPAB	50	54	9
12	EtOAc	DIPAB	60	100	6
13	EtOAc	DICAB	30	38	1
14	EtOAc	DICAB	40	100	1
15	EtOAc	DICAB	50	66	2
16	EtOAc	DICAB	60	100	37
17	MTBE	DIPAB	30	30	1
18	MTBE	DIPAB	40	78	33
19	MTBE	DIPAB	50	67	20
20	MTBE	DIPAB	60	67	24
21	MTBE	DICAB	30	58	27
22	MTBE	DICAB	40	100	77
23	MTBE	DICAB	50	100	73
24	MTBE	DICAB	60	100	75

[a]Disappearance of the starting material, estimated by ^{1}H NMR analysis of the crude mixture using 2-fluoro-4-nitrotoluene as an internal standard. [b]Yield estimated by ^{19}F NMR analysis of the crude mixture using 2-fluoro-4-nitrotoluene as an internal standard.

4. Evaluation of the initial temperature

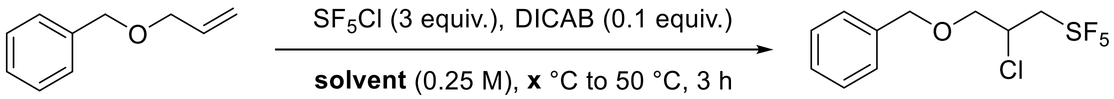

1 **2a**

Solvent	x °C	Conversion (%)[a]	Yield (%)[b]
EtOAc	0	100	93
EtOAc	rt	100	71
hexane	0	40	2
hexane	rt	100	81
MTBE	0	100	75
MTBE	rt	100	79

[a]Disappearance of the starting material, estimated by ^{1}H NMR analysis of the crude mixture using 2-fluoro-4-nitrotoluene as an internal standard. [b]Yield estimated by ^{19}F NMR analysis of the crude mixture using 2-fluoro-4-nitrotoluene as an internal standard.

NMR spectra

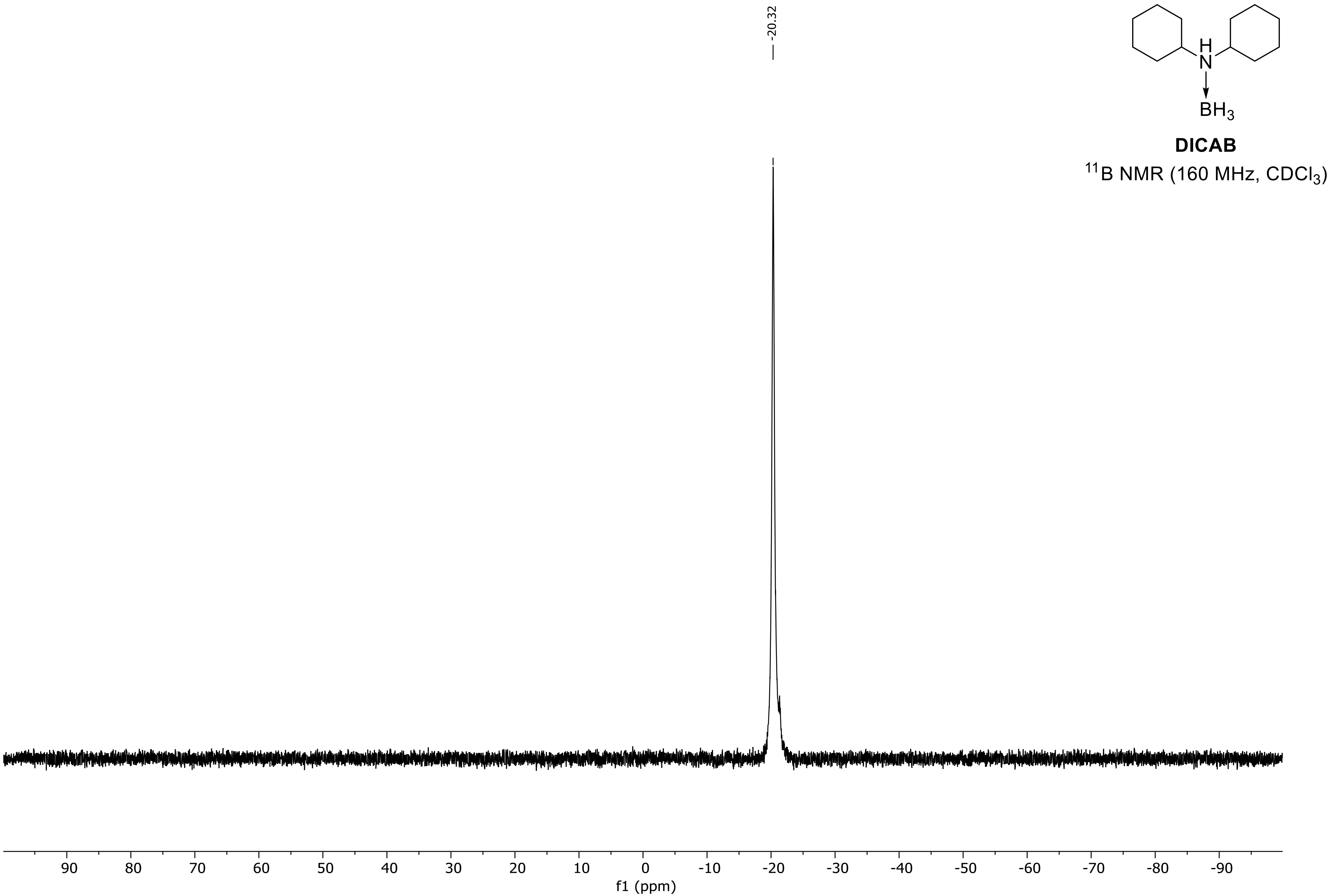

-20.32
H
N
BH_3
DICAB
^{11}B NMR (160 MHz, $CDCl_3$)
90 80 70 60 50 40 30 20 10 0 -10 -20 -30 -40 -50 -60 -70 -80 -90
f1 (ppm)

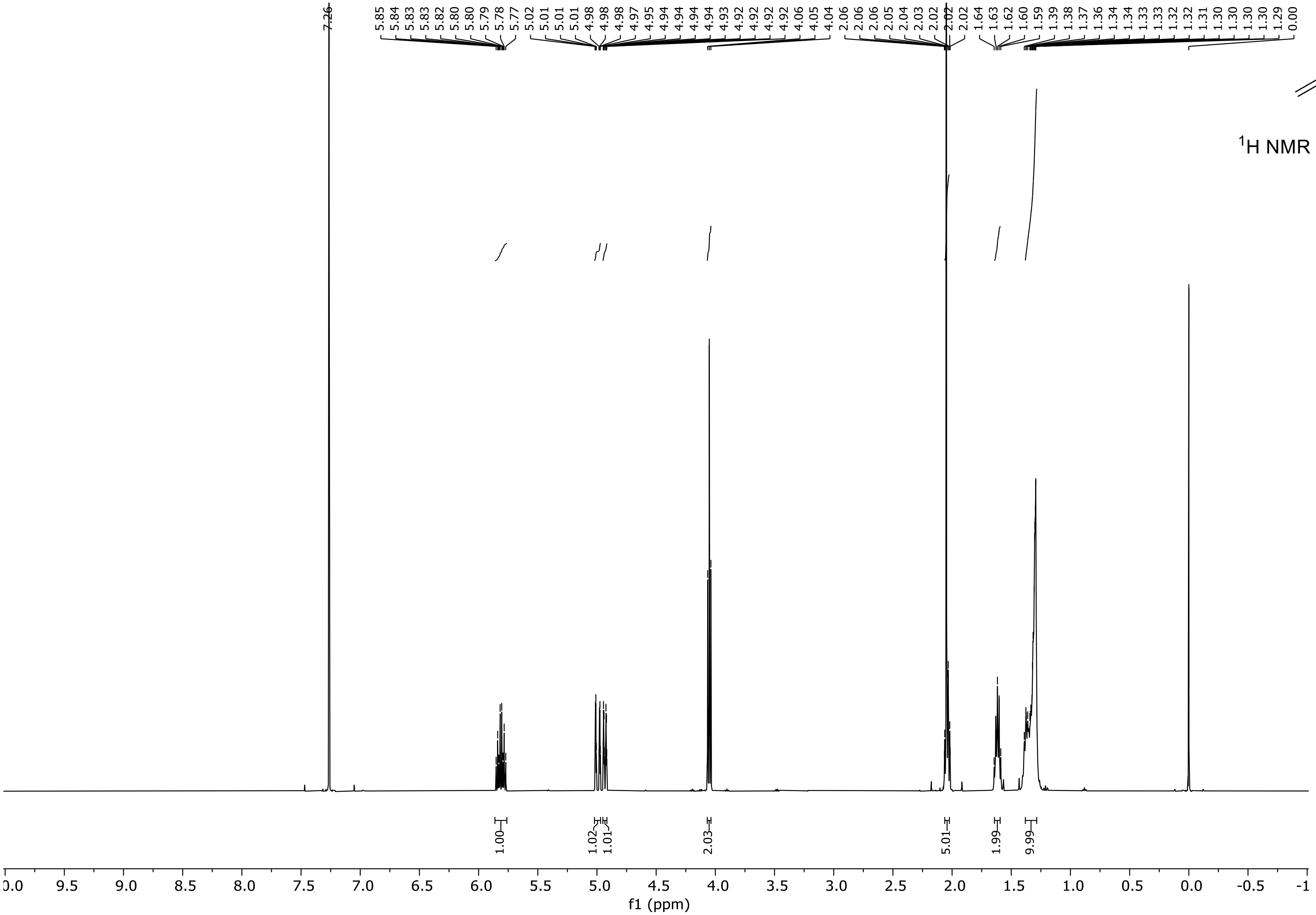

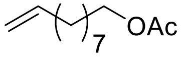

^1H NMR (500 MHz, $CDCl_3$)

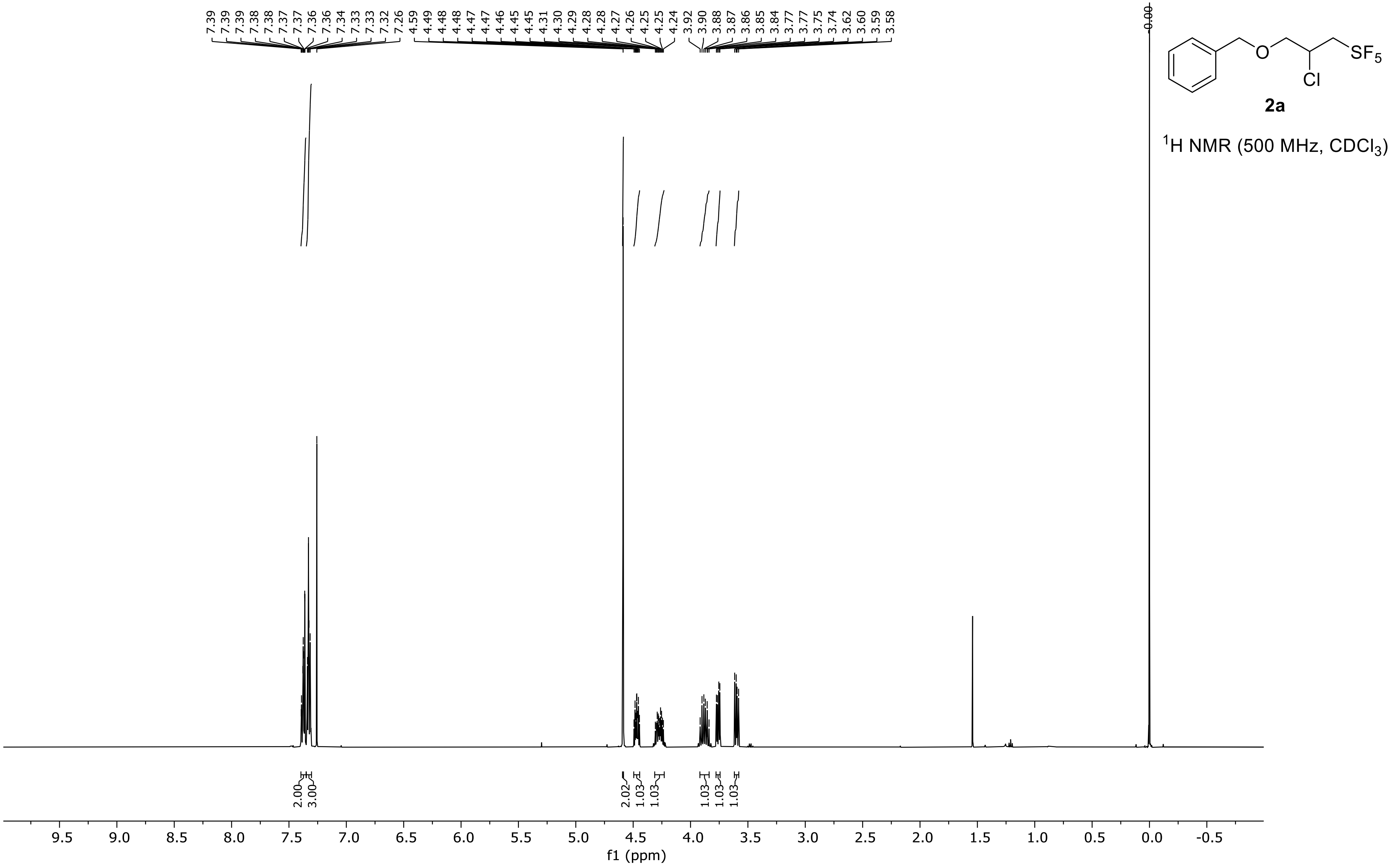
7.39 7.39 7.39 7.38 7.38 7.37 7.37 7.36 7.36 7.34 7.33 7.33 7.32 7.26 4.59 4.49 4.48 4.48 4.47 4.47 4.46 4.45 4.45 4.31 4.30 4.29 4.28 4.28 4.27 4.26 4.25 4.25 4.24 3.92 3.90 3.88 3.87 3.86 3.85 3.84 3.77 3.77 3.75 3.74 3.62 3.60 3.59 3.58
0.00
O
SF5
Cl
2a
1H NMR (500 MHz, CDCl3)
2.00 3.00 2.02 1.03 1.03 1.03 1.03 1.03
9.5 9.0 8.5 8.0 7.5 7.0 6.5 6.0 5.5 5.0 4.5 4.0 3.5 3.0 2.5 2.0 1.5 1.0 0.5 0.0 -0.5
f1 (ppm)

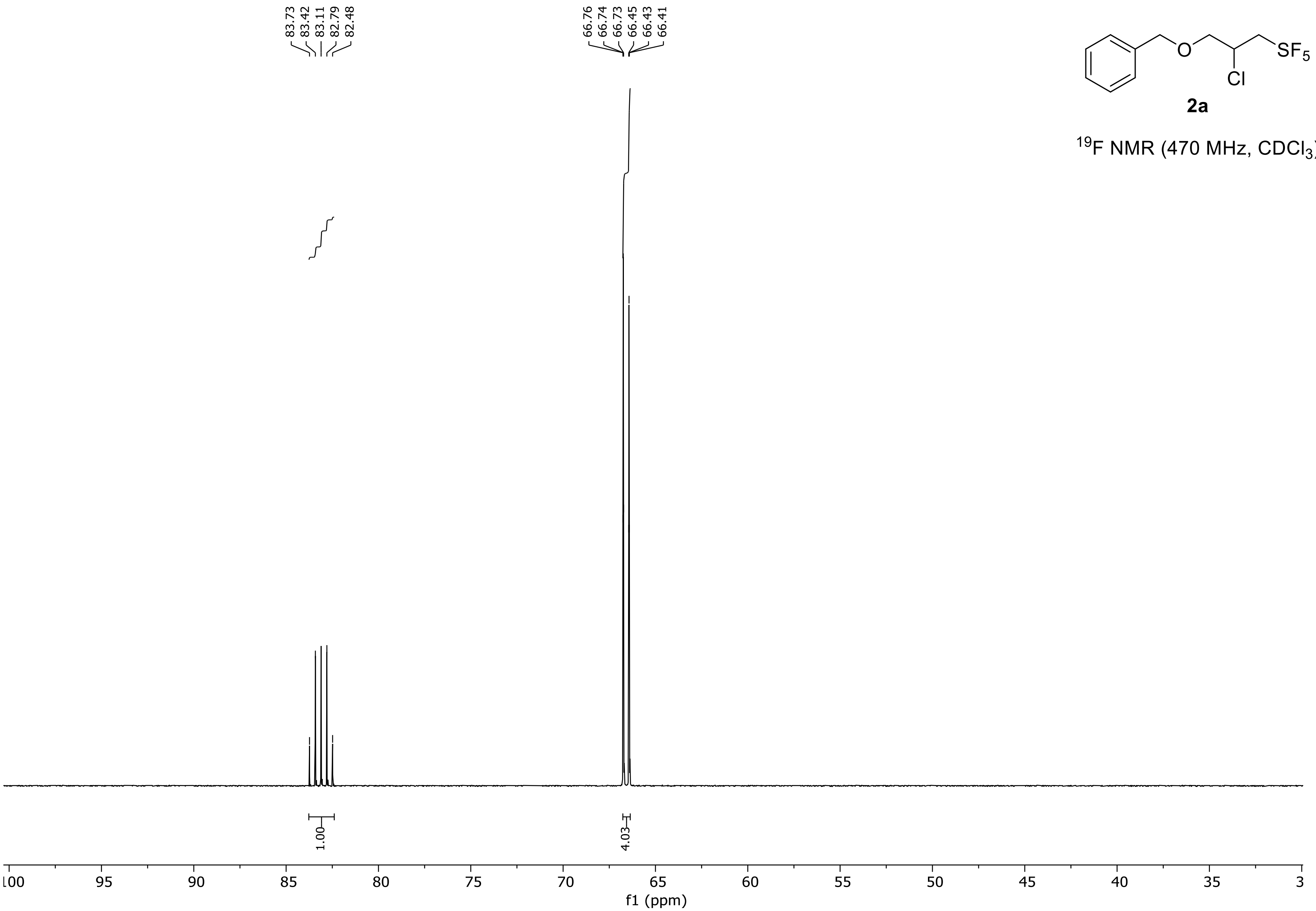

2a

^{19}F NMR (470 MHz, $CDCl_3$)

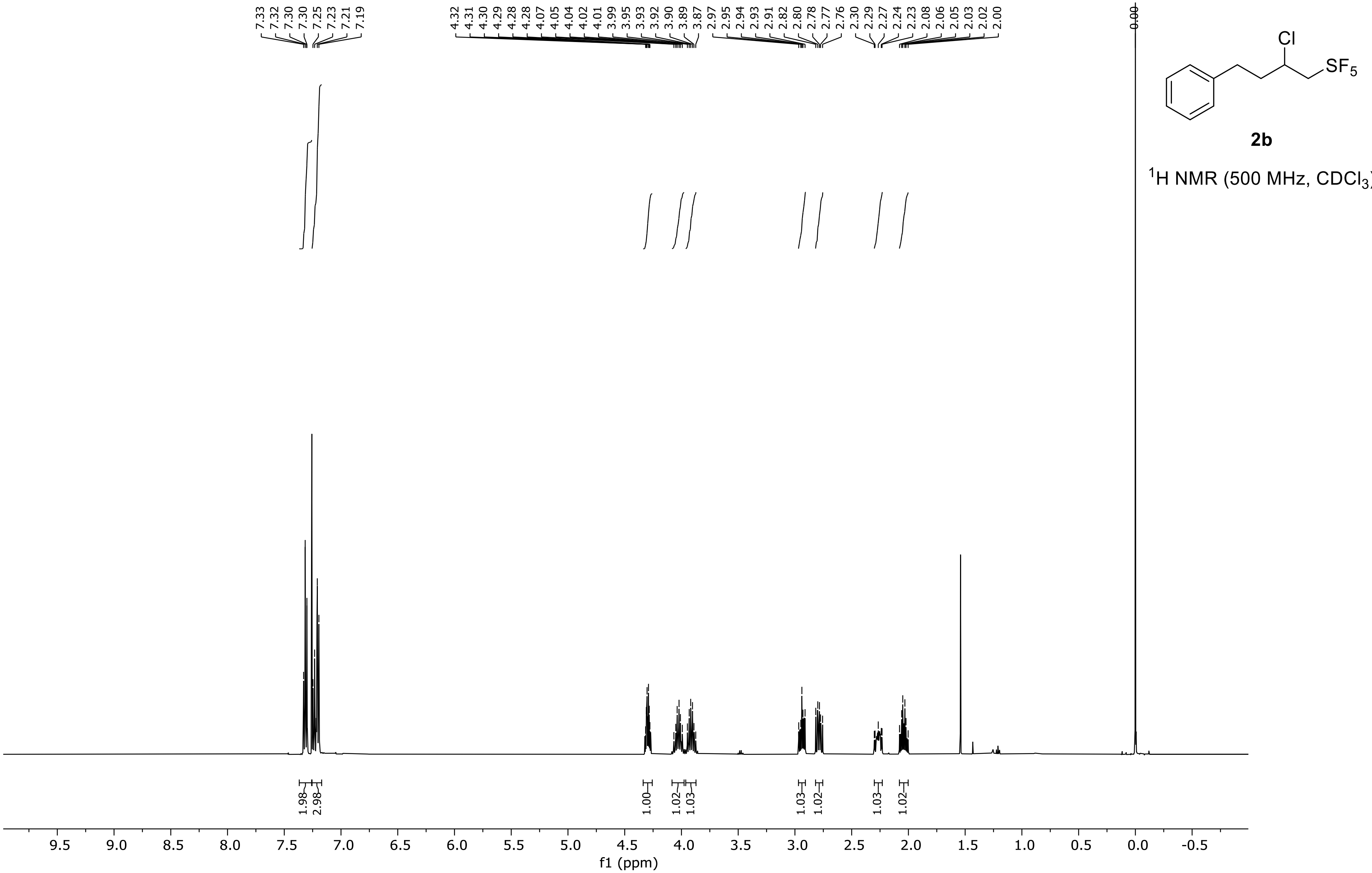

2b

^1H NMR (500 MHz, $CDCl_3$)

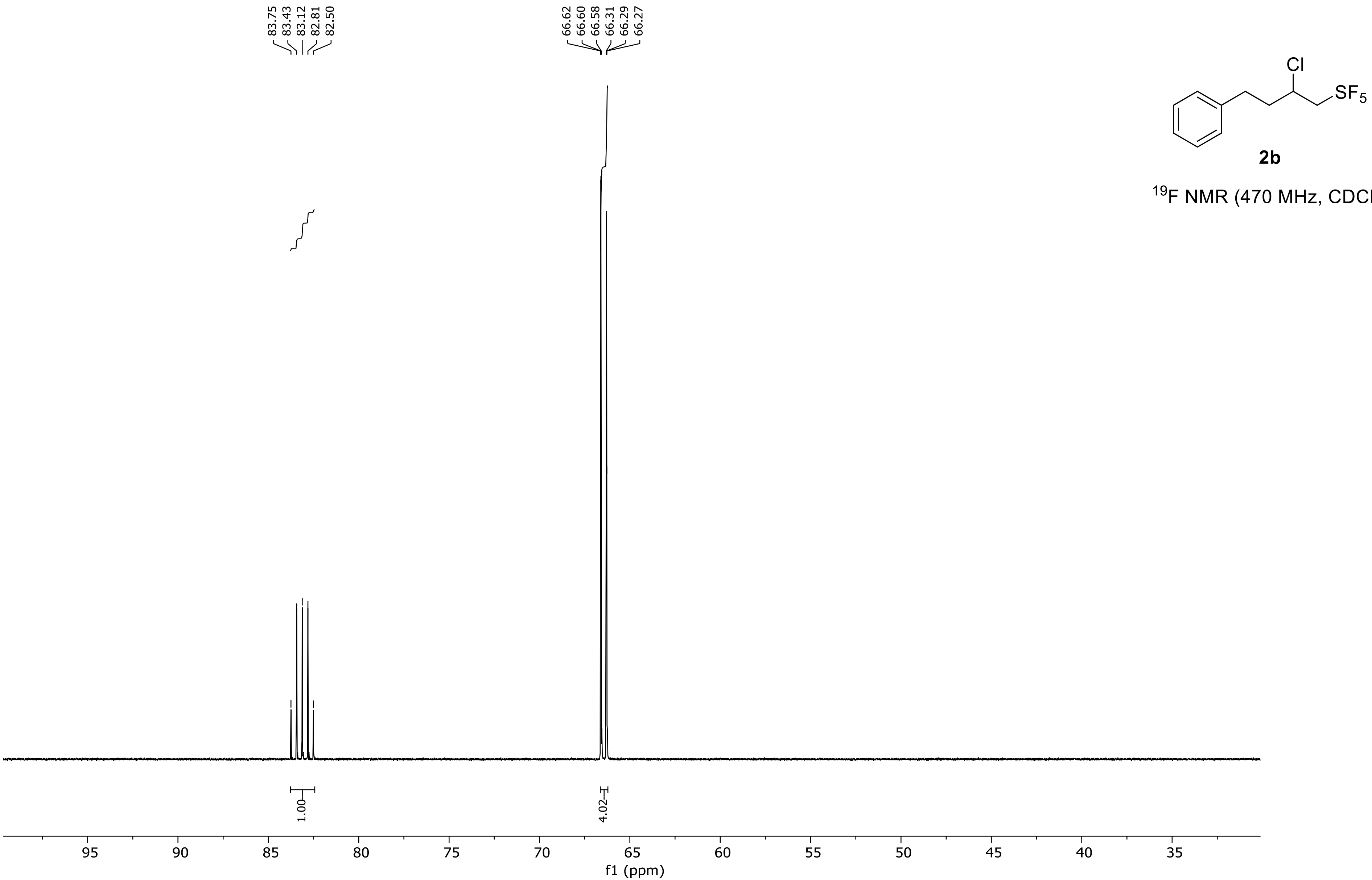

2b

^{19}F NMR (470 MHz, $CDCl_3$)

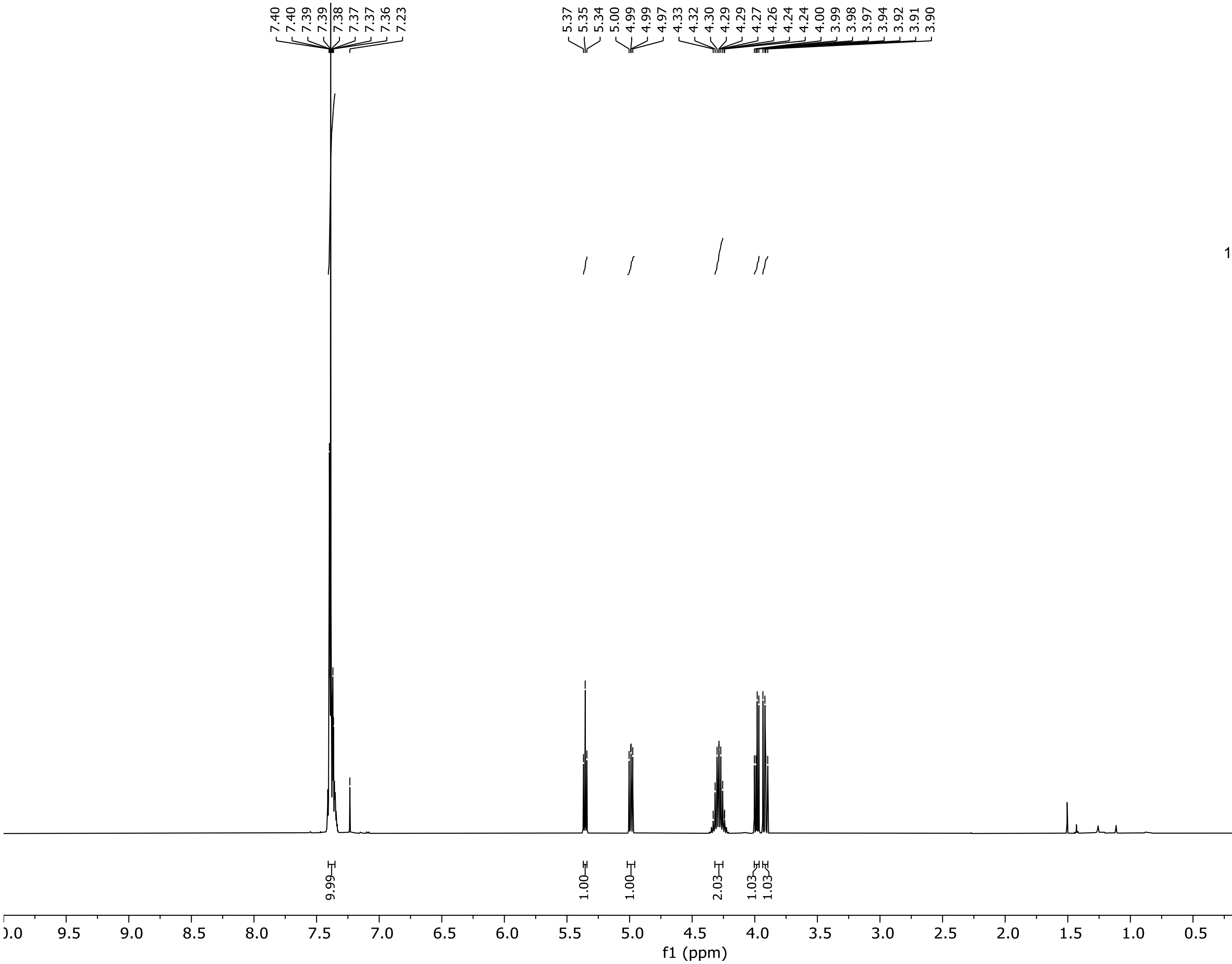

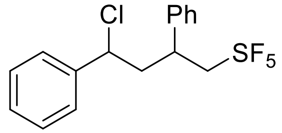

2d

^{1}H NMR (500 MHz, $CDCl_3$)

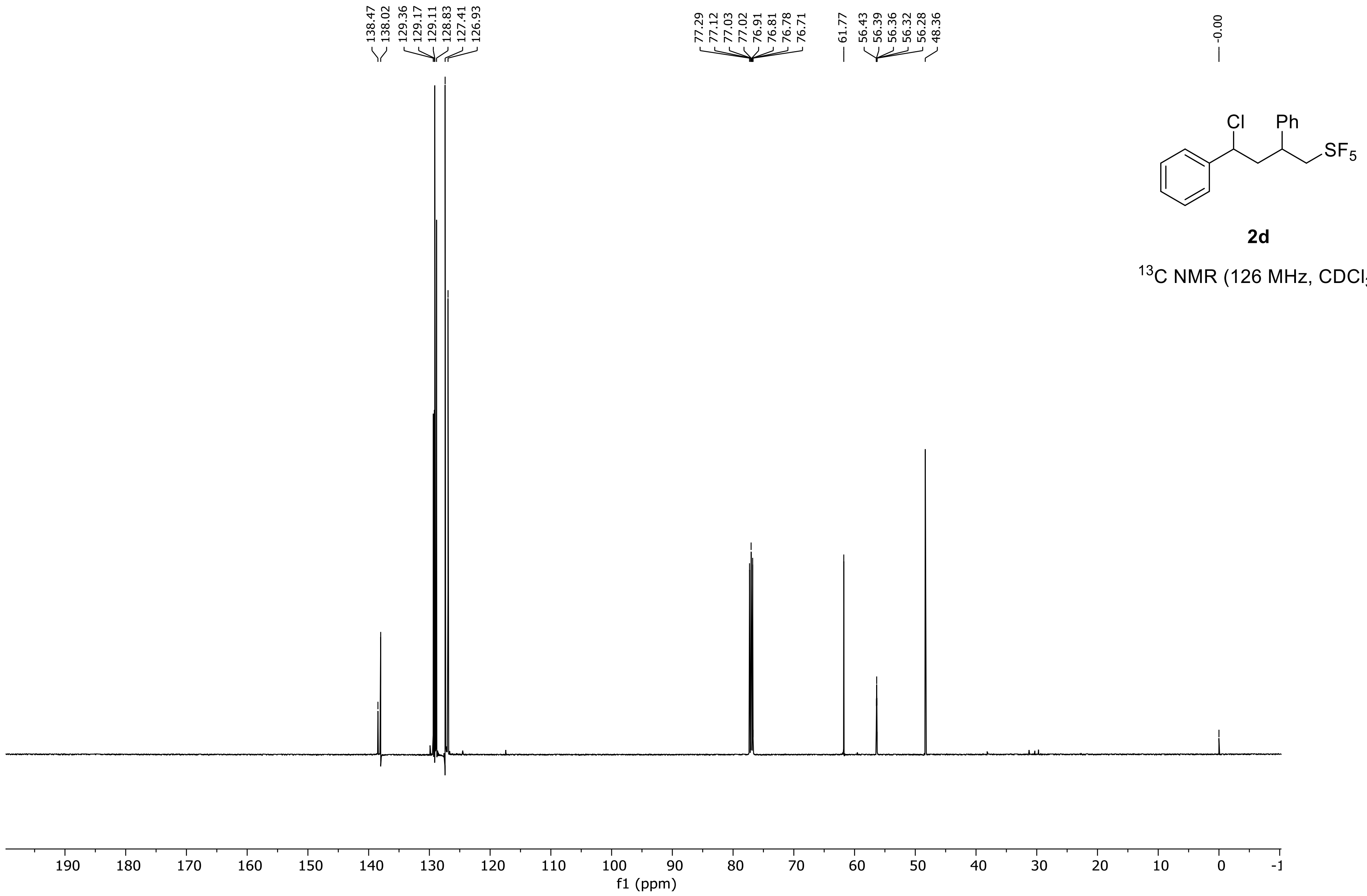

2d

^{13}C NMR (126 MHz, $CDCl_3$)

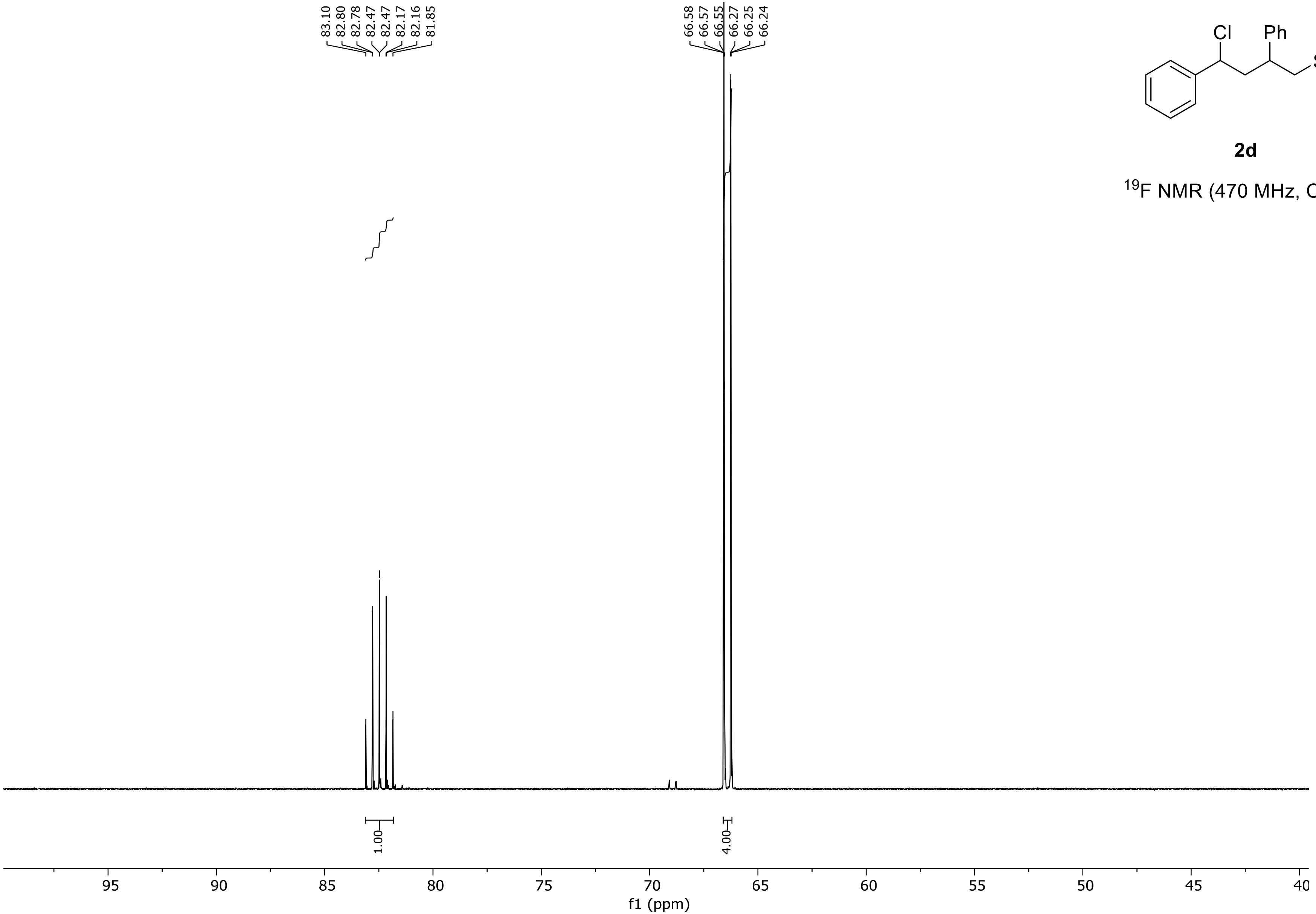

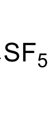

2d

^{19}F NMR (470 MHz, $CDCl_3$)

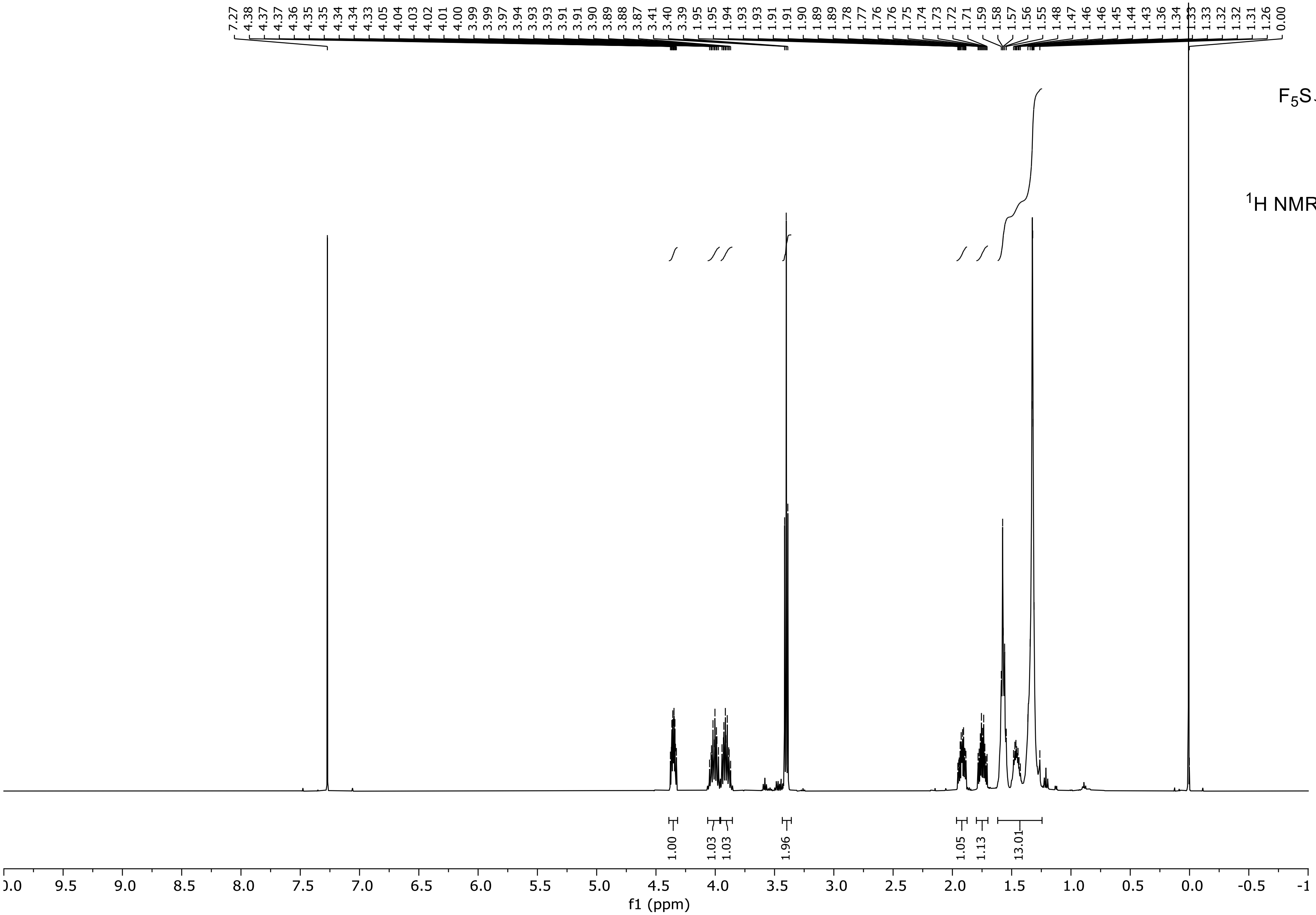

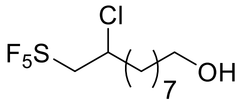

2e

^{1}H NMR (500 MHz, $CDCl_3$)

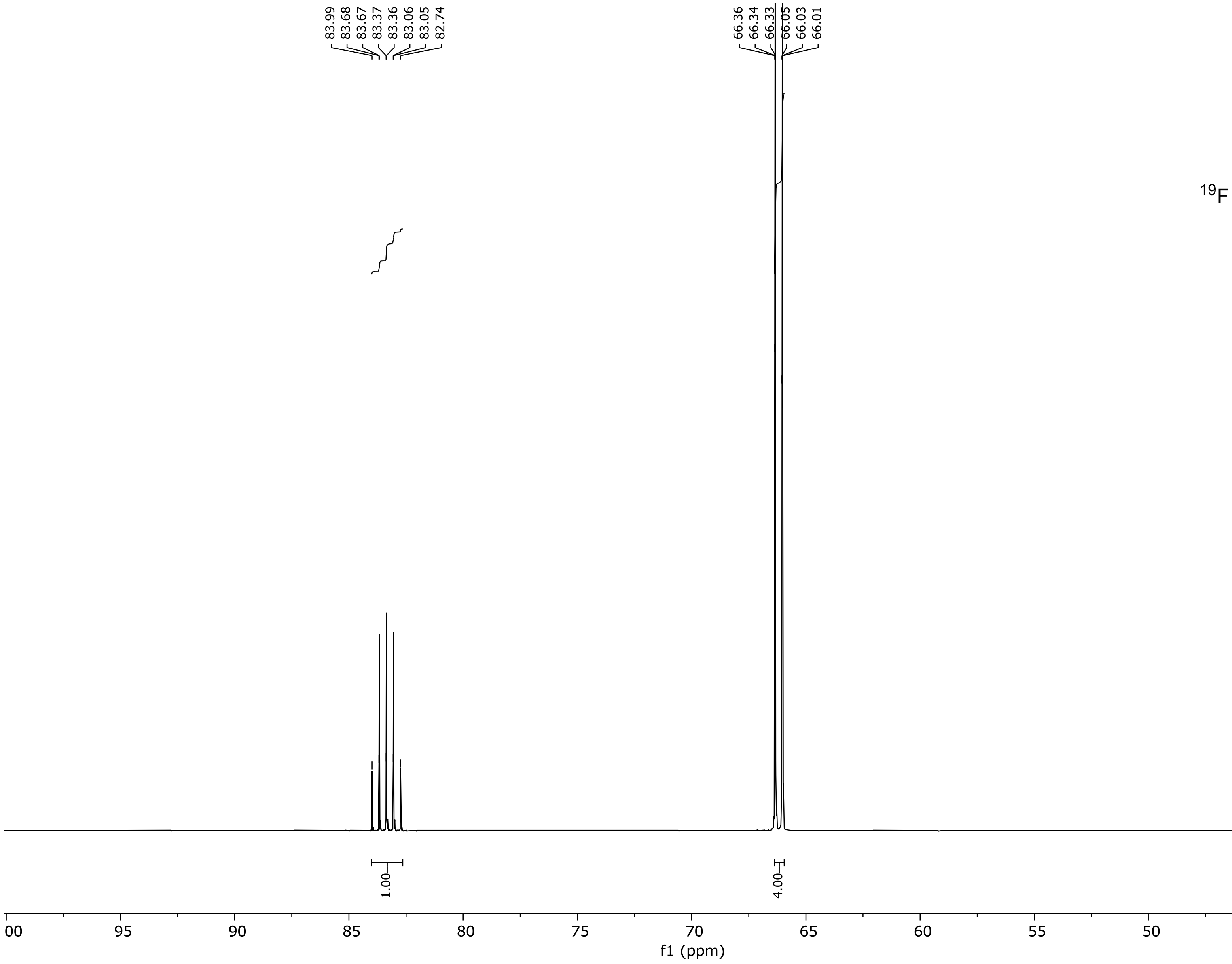

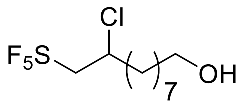

2e

^{19}F NMR (470 MHz, $CDCl_3$)

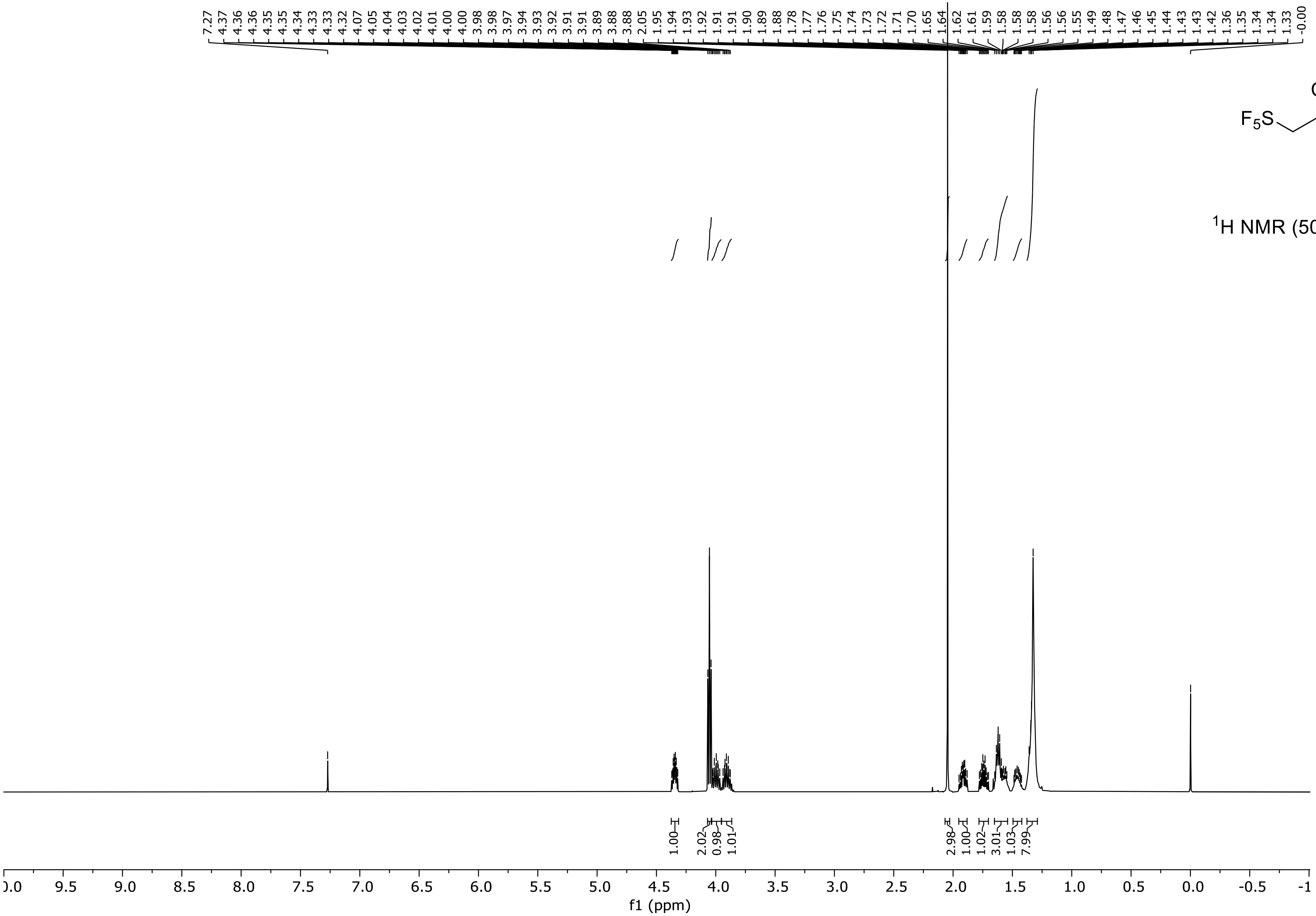

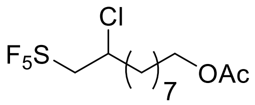

2f

^{1}H NMR (500 MHz, $CDCl_3$)

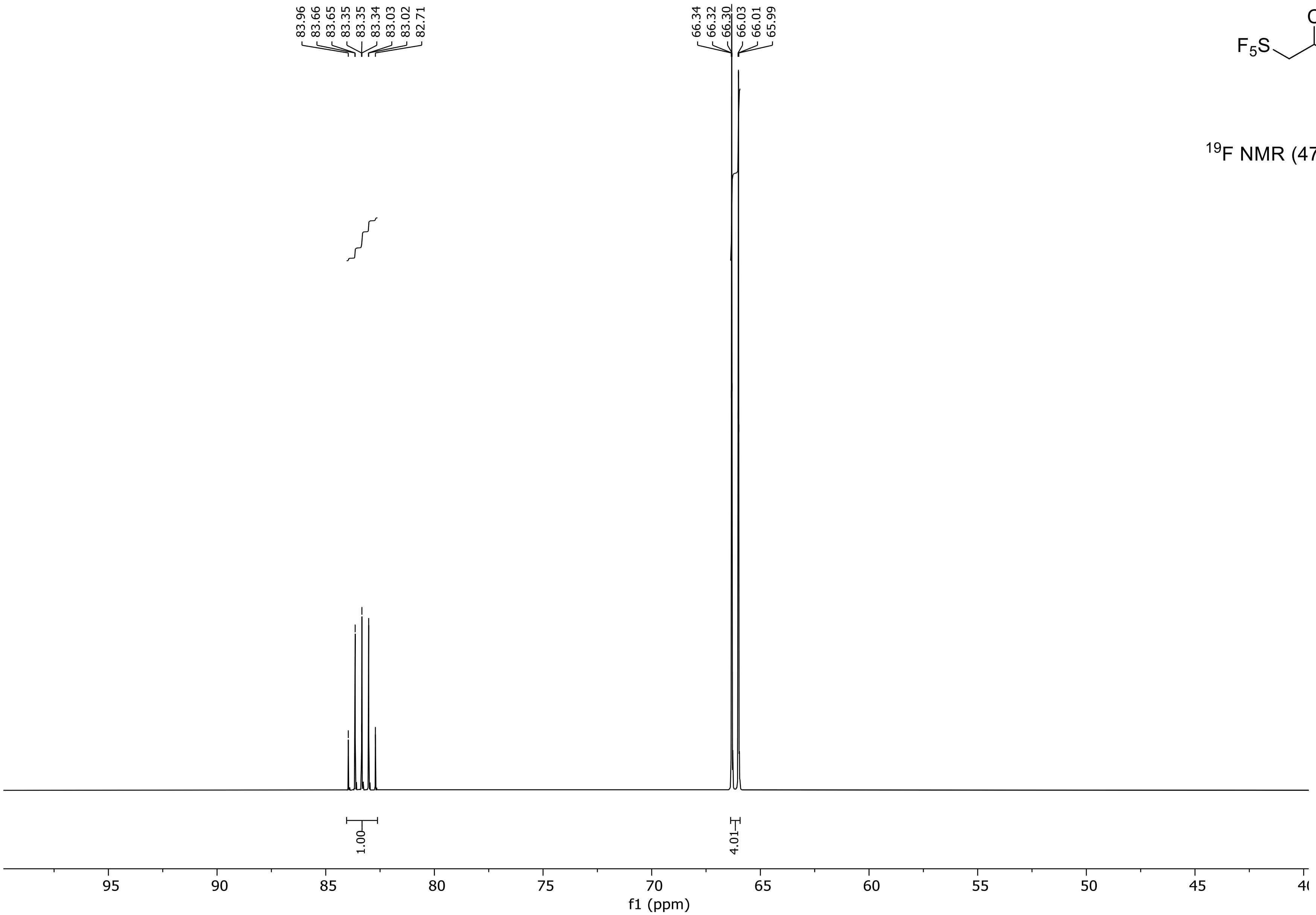

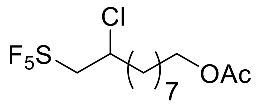

2f

^{19}F NMR (470 MHz, $CDCl_3$)

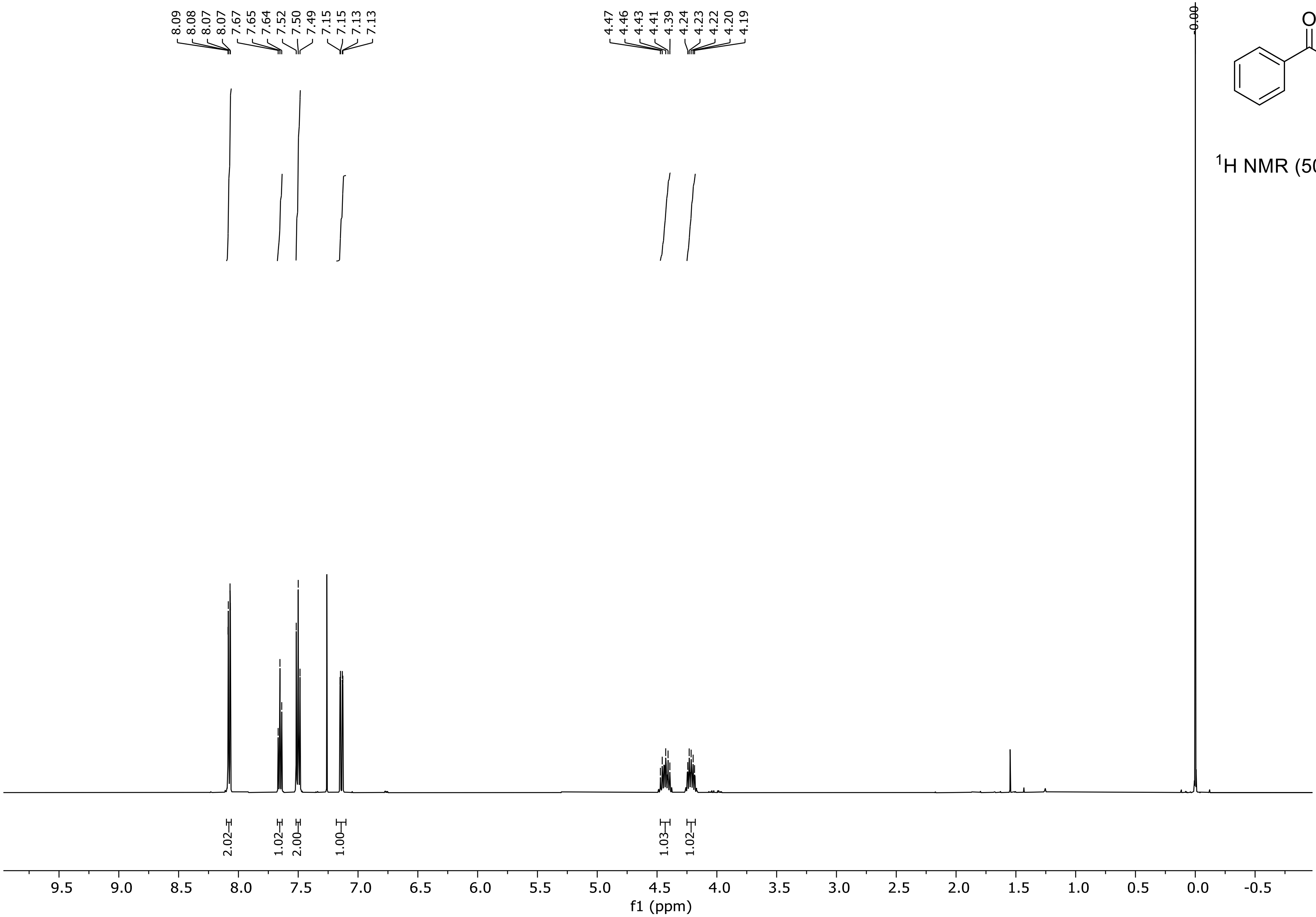

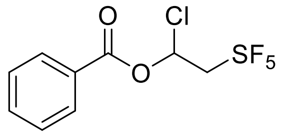

2g

^1H NMR (500 MHz, $CDCl_3$)

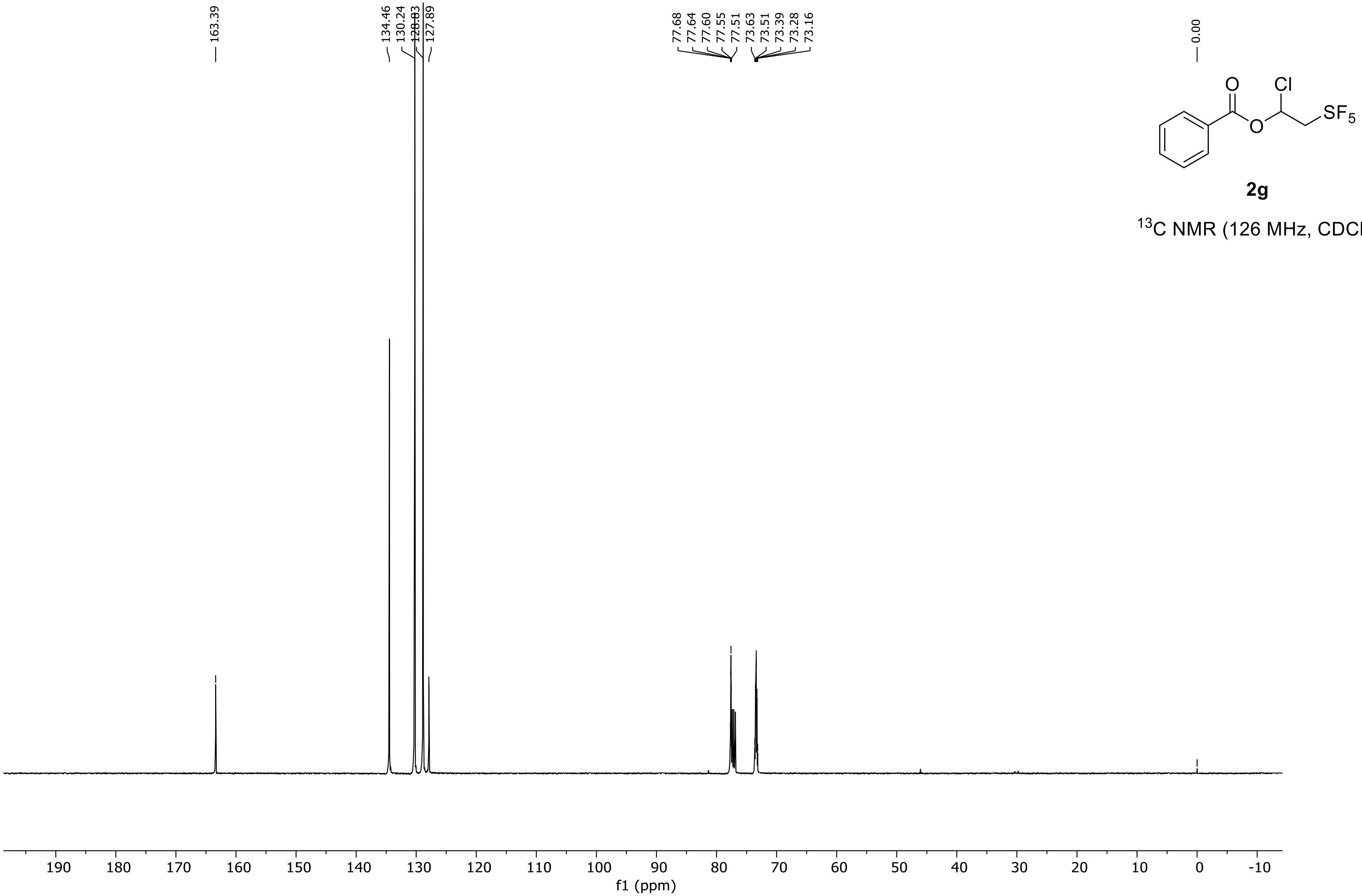

2g

^{13}C NMR (126 MHz, $CDCl_3$)

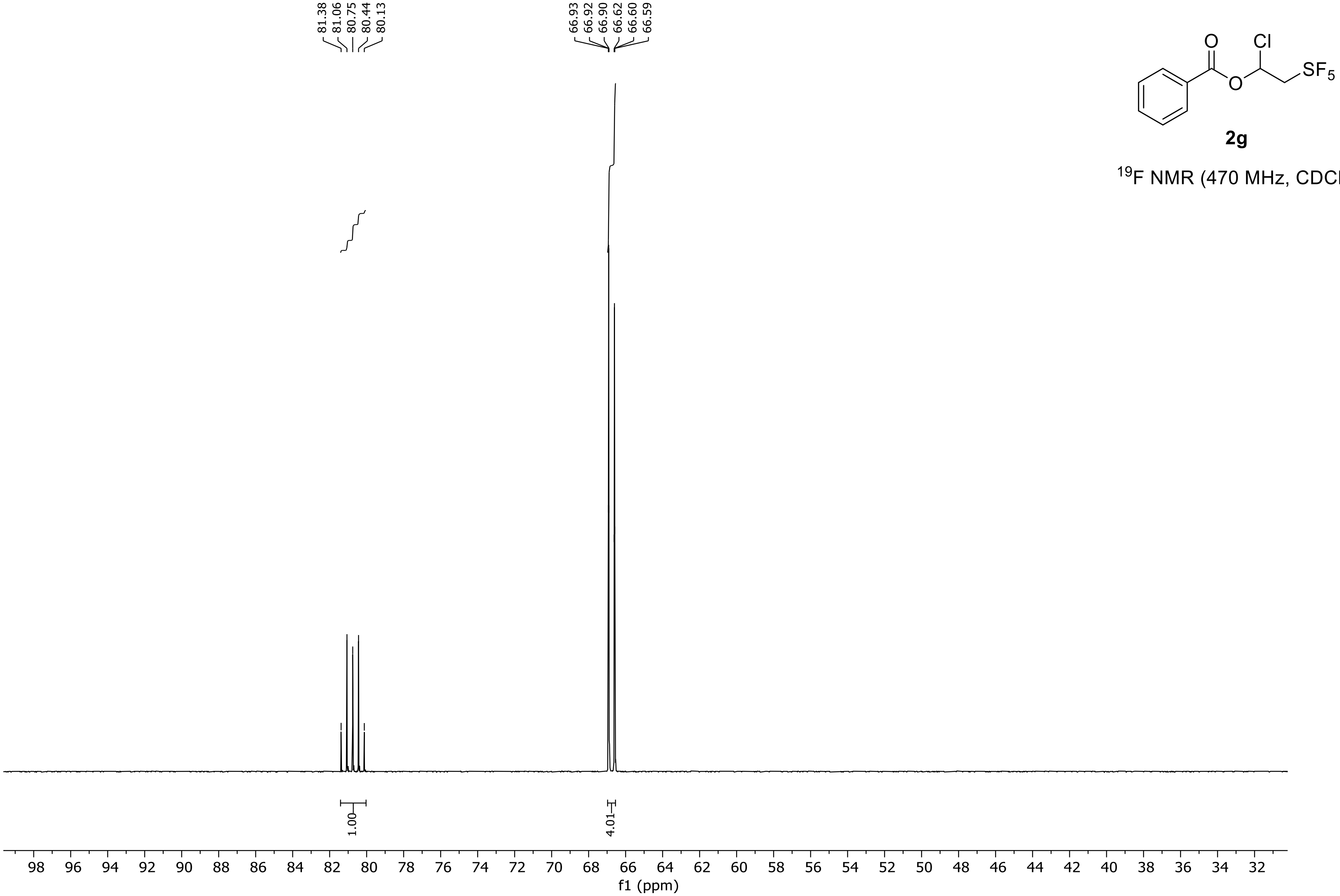

2g

^{19}F NMR (470 MHz, $CDCl_3$)

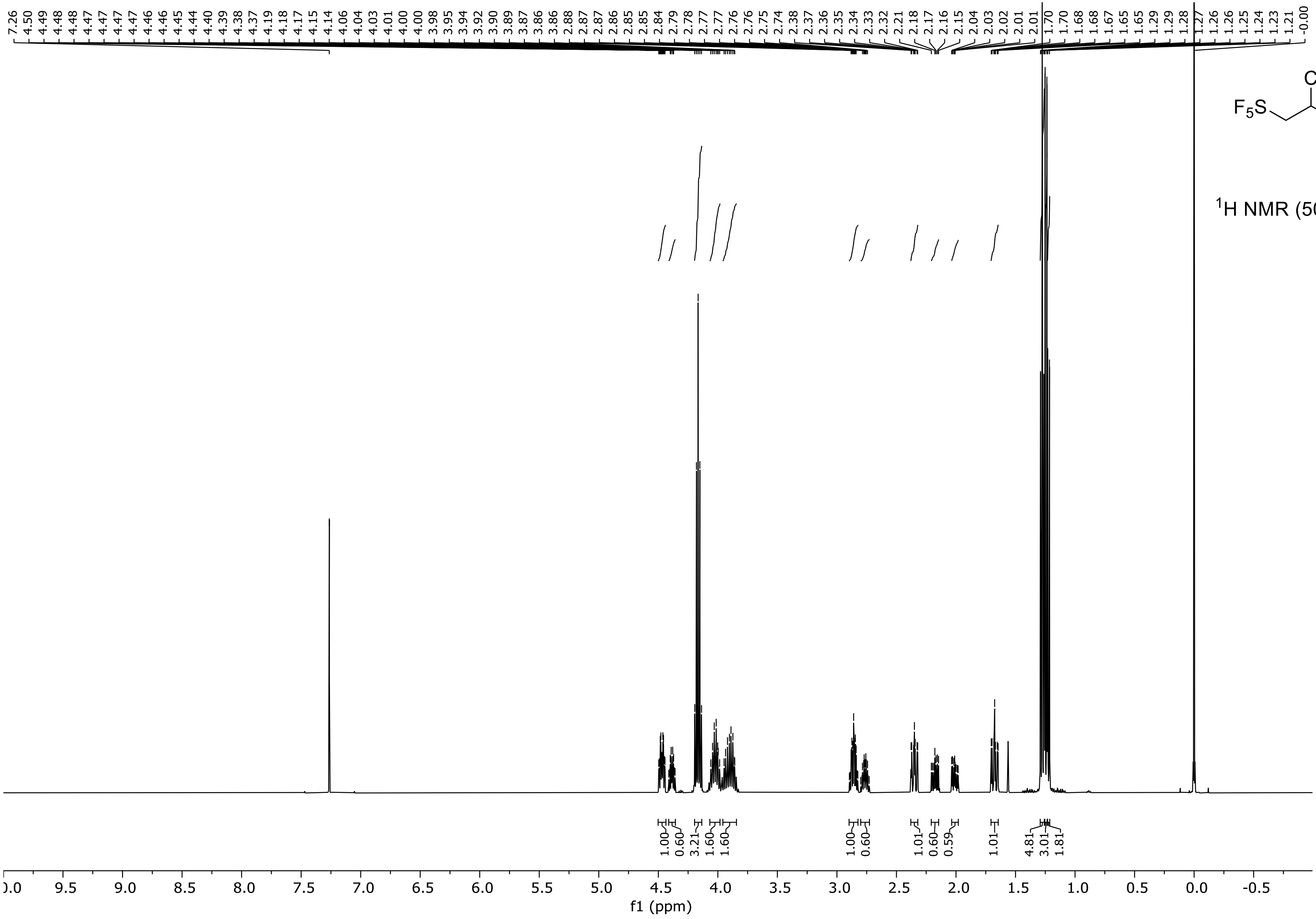

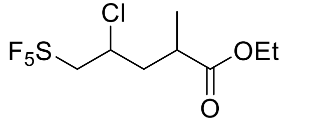

2h

^1H NMR (500 MHz, $CDCl_3$)

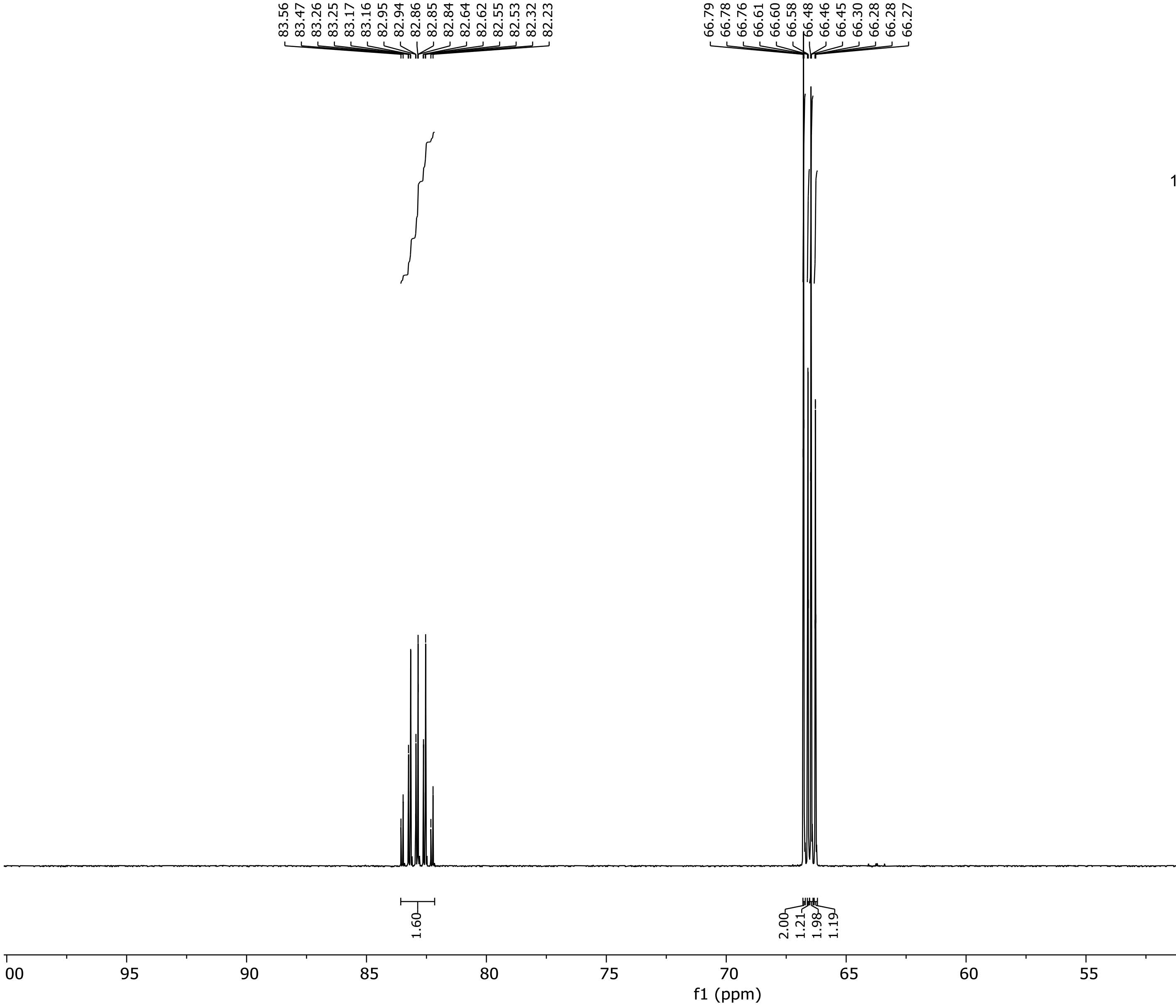

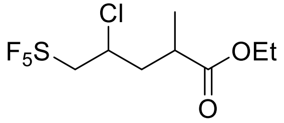

2h

^{19}F NMR (470 MHz, $CDCl_3$)

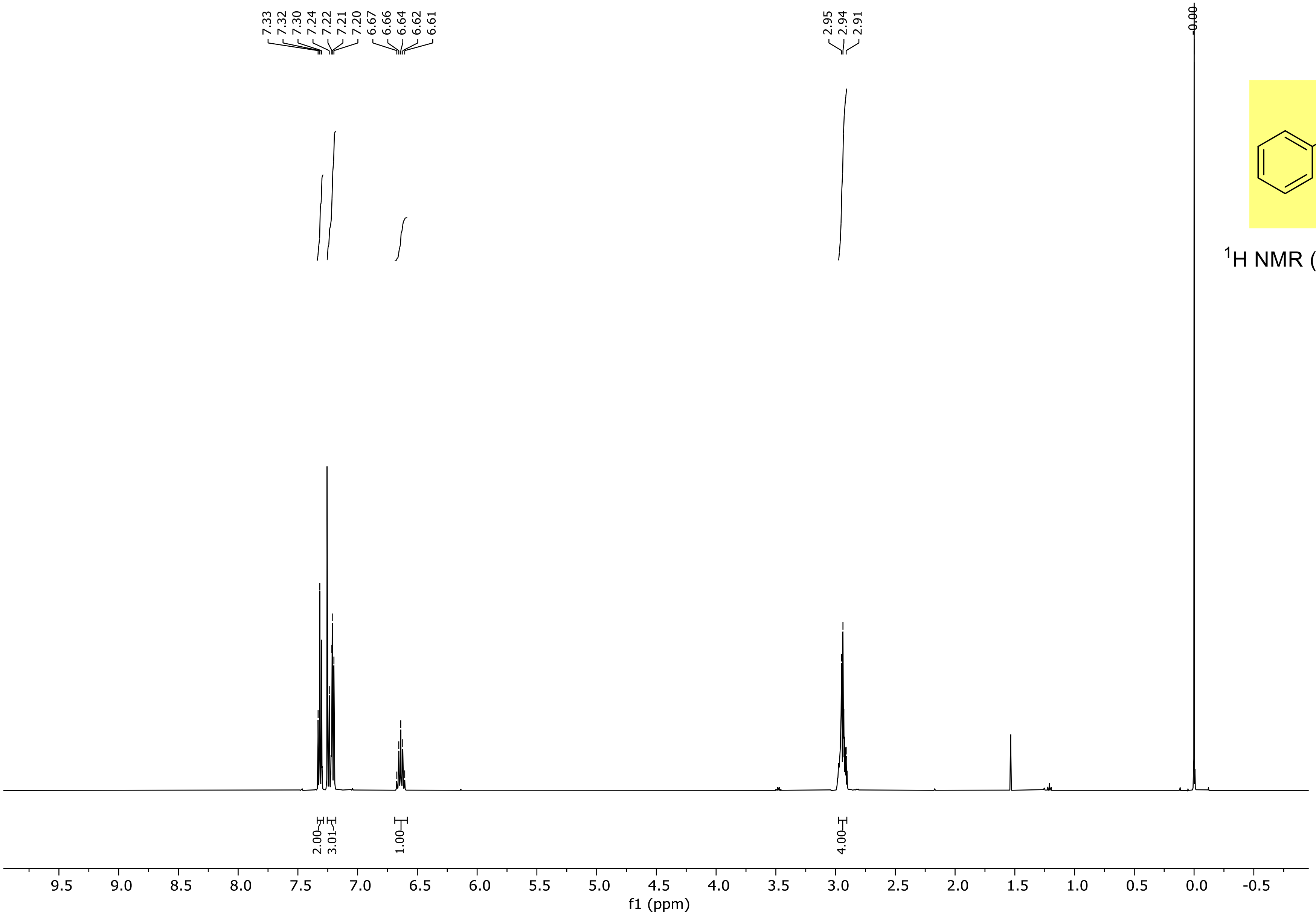

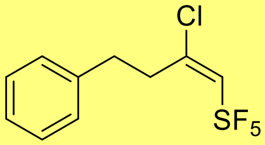

2i

^{1}H NMR (500 MHz, $CDCl_3$)

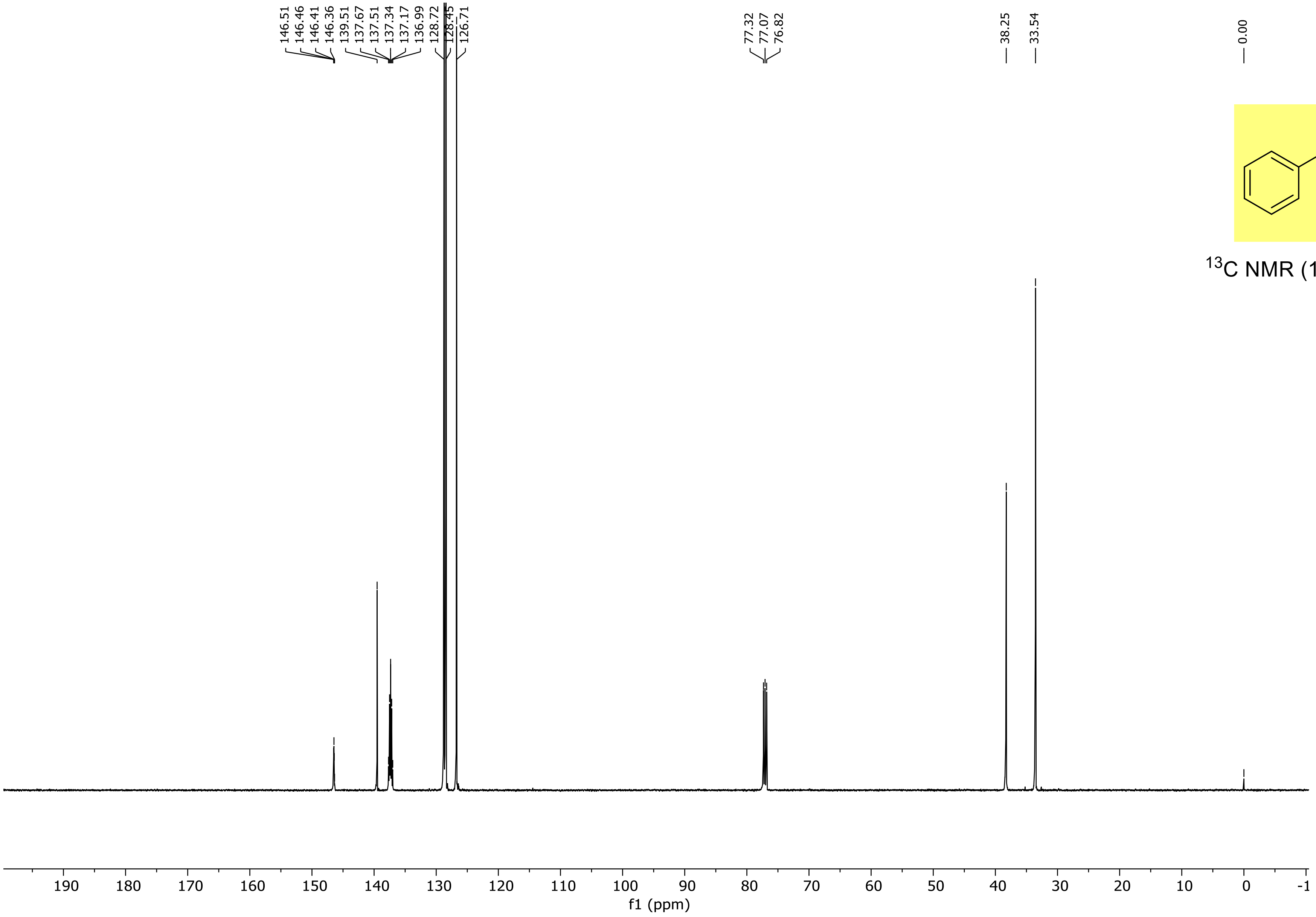

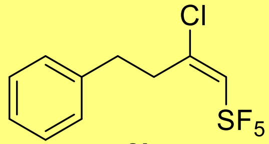

2i

^{13}C NMR (126 MHz, $CDCl_3$)

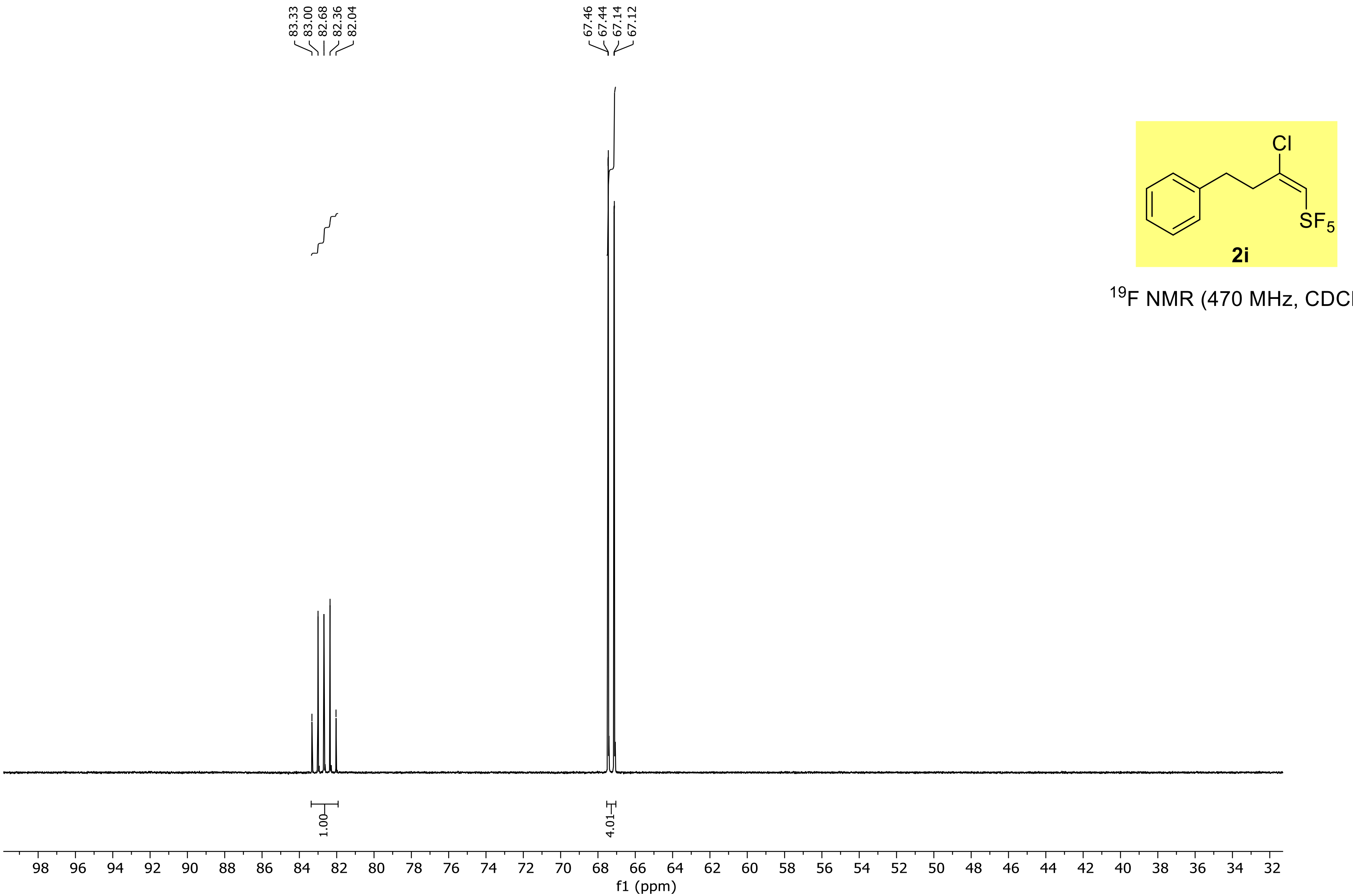

2i

^{19}F NMR (470 MHz, $CDCl_3$)

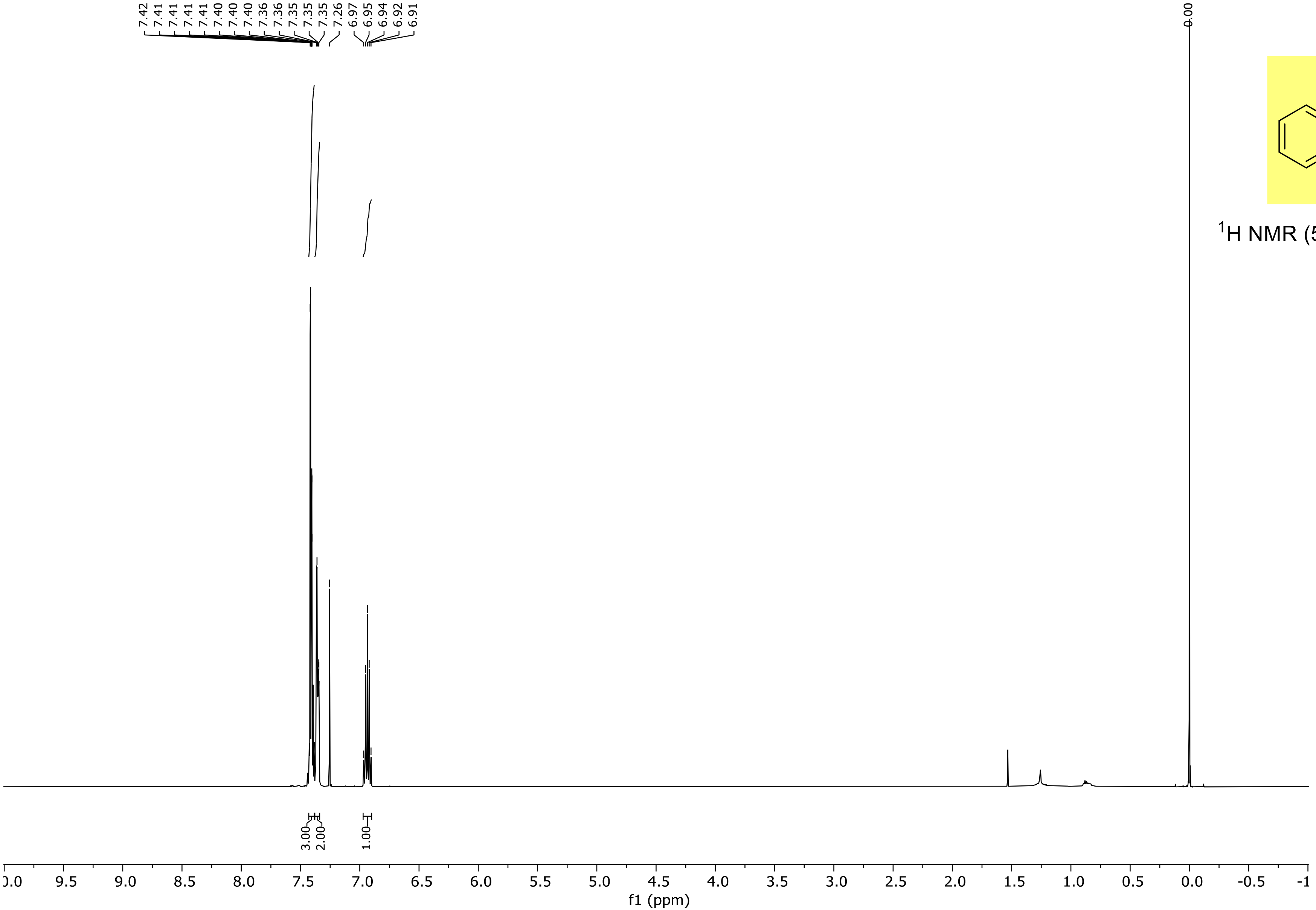

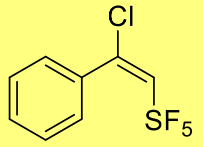

2j

^{1}H NMR (500 MHz, $CDCl_3$)

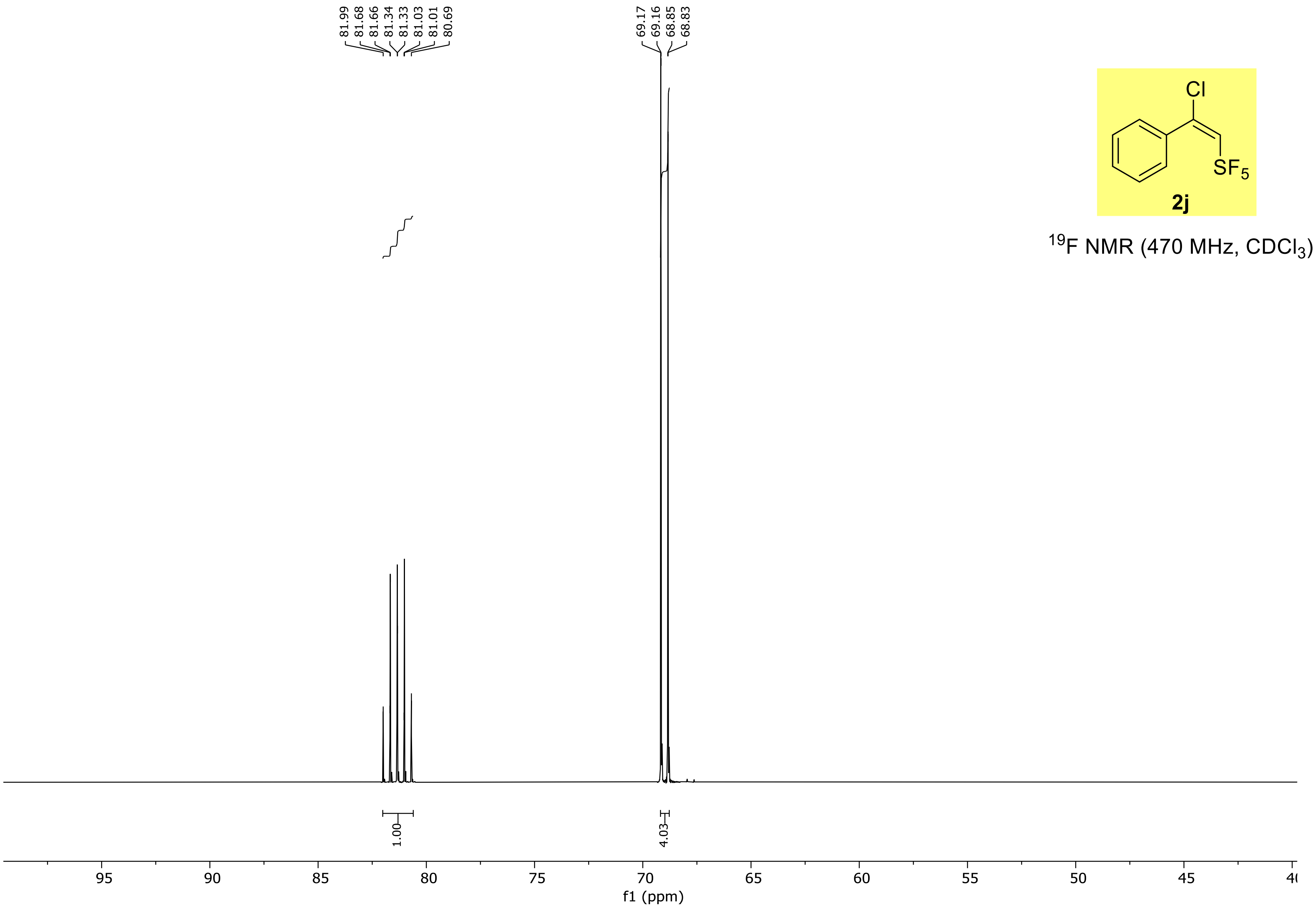
81.99
81.68
81.66
81.34
81.33
81.03
81.01
80.69
69.17
69.16
68.85
68.83
Cl
SF5
2j
19F NMR (470 MHz, CDCl3)
1.00
4.03
95
90
85
80
75
70
65
60
55
50
45
f1 (ppm)

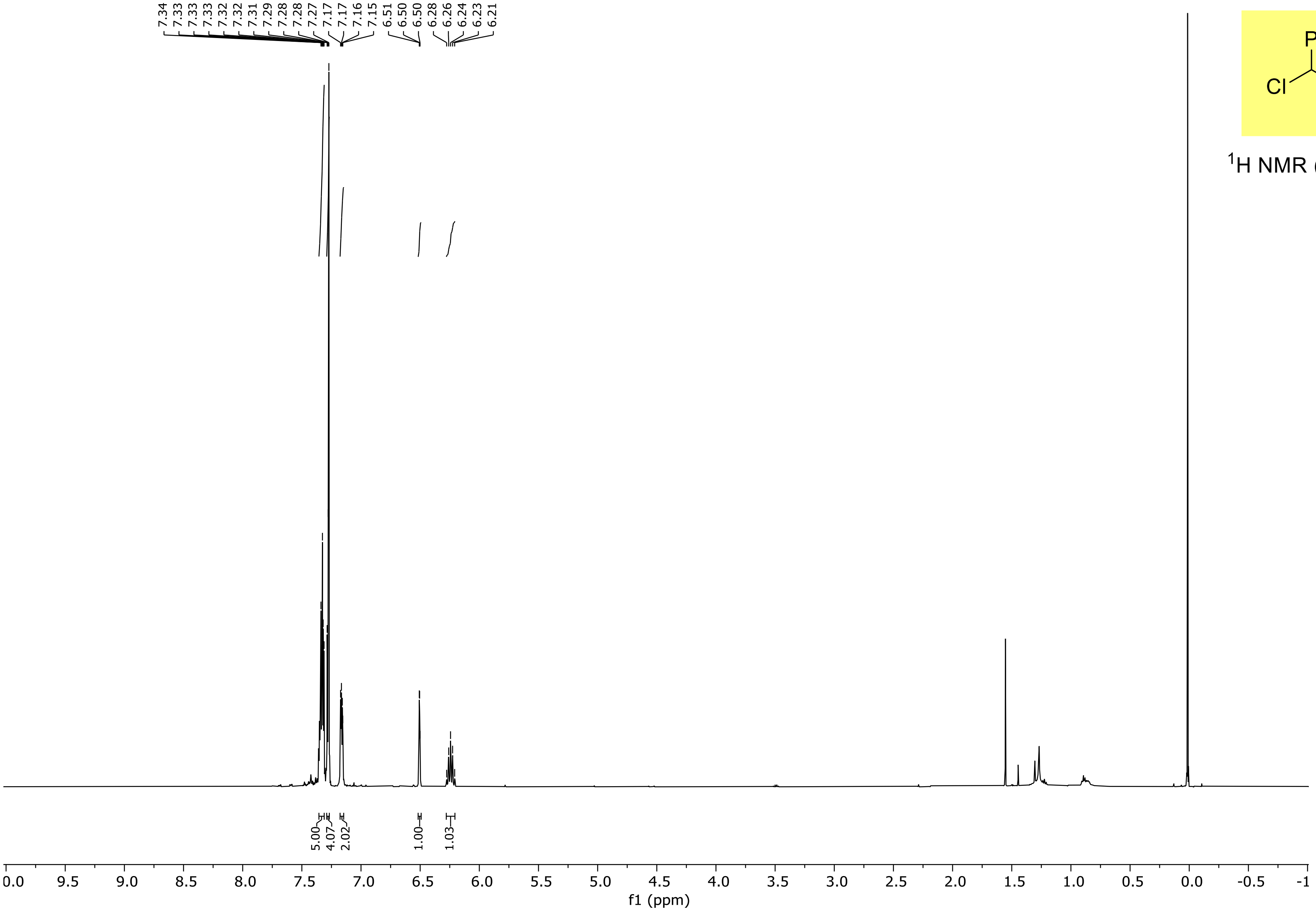

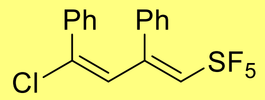

2k

^{1}H NMR (500 MHz, $CDCl_3$)

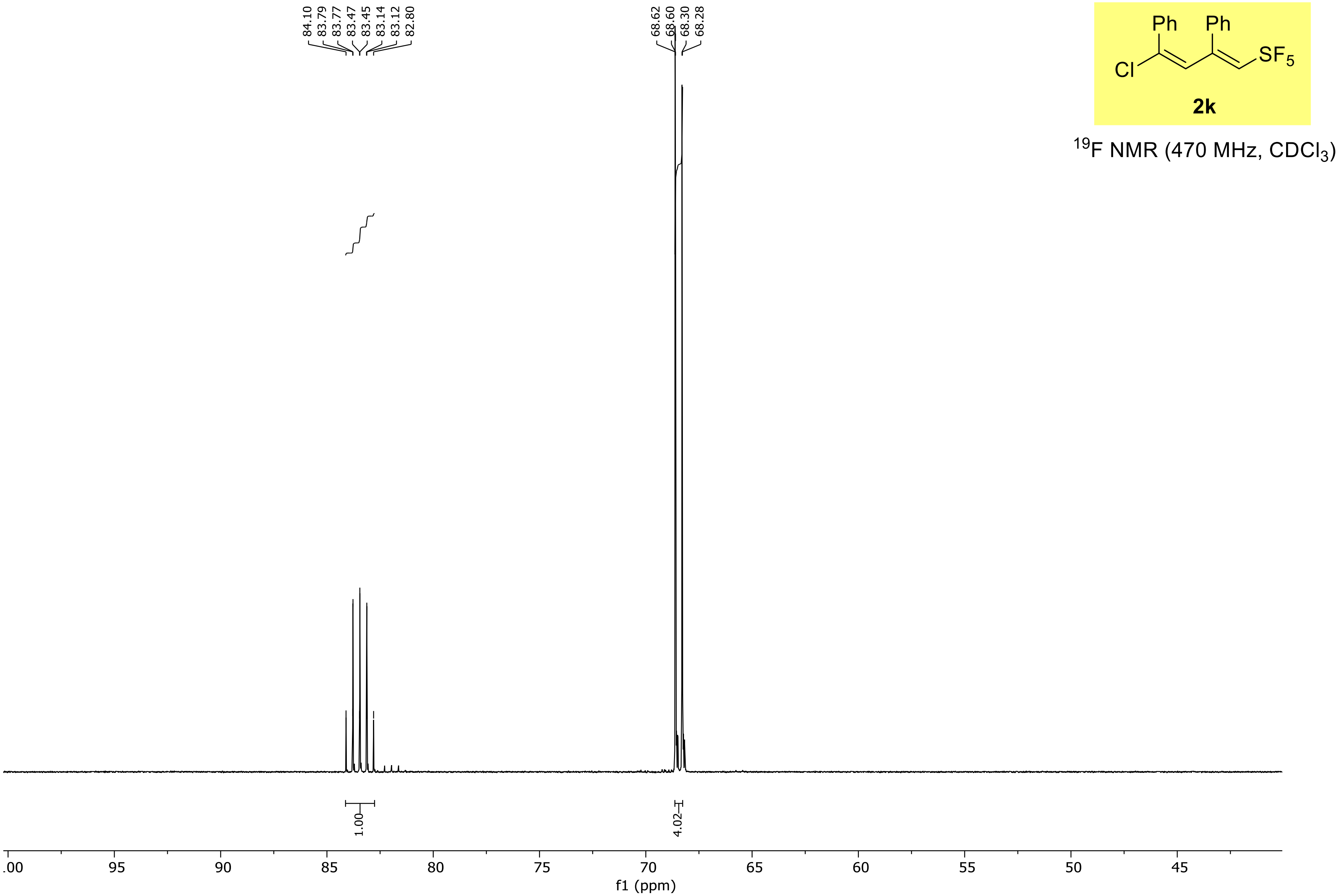
84.10
83.79
83.77
83.47
83.45
83.14
83.12
82.80
68.62
68.60
68.30
68.28
Ph
Ph
Cl
SF5
2k
19F NMR (470 MHz, CDCl3)
1.00
4.02
.00
95
90
85
80
75
70
65
60
55
50
45
f1 (ppm)

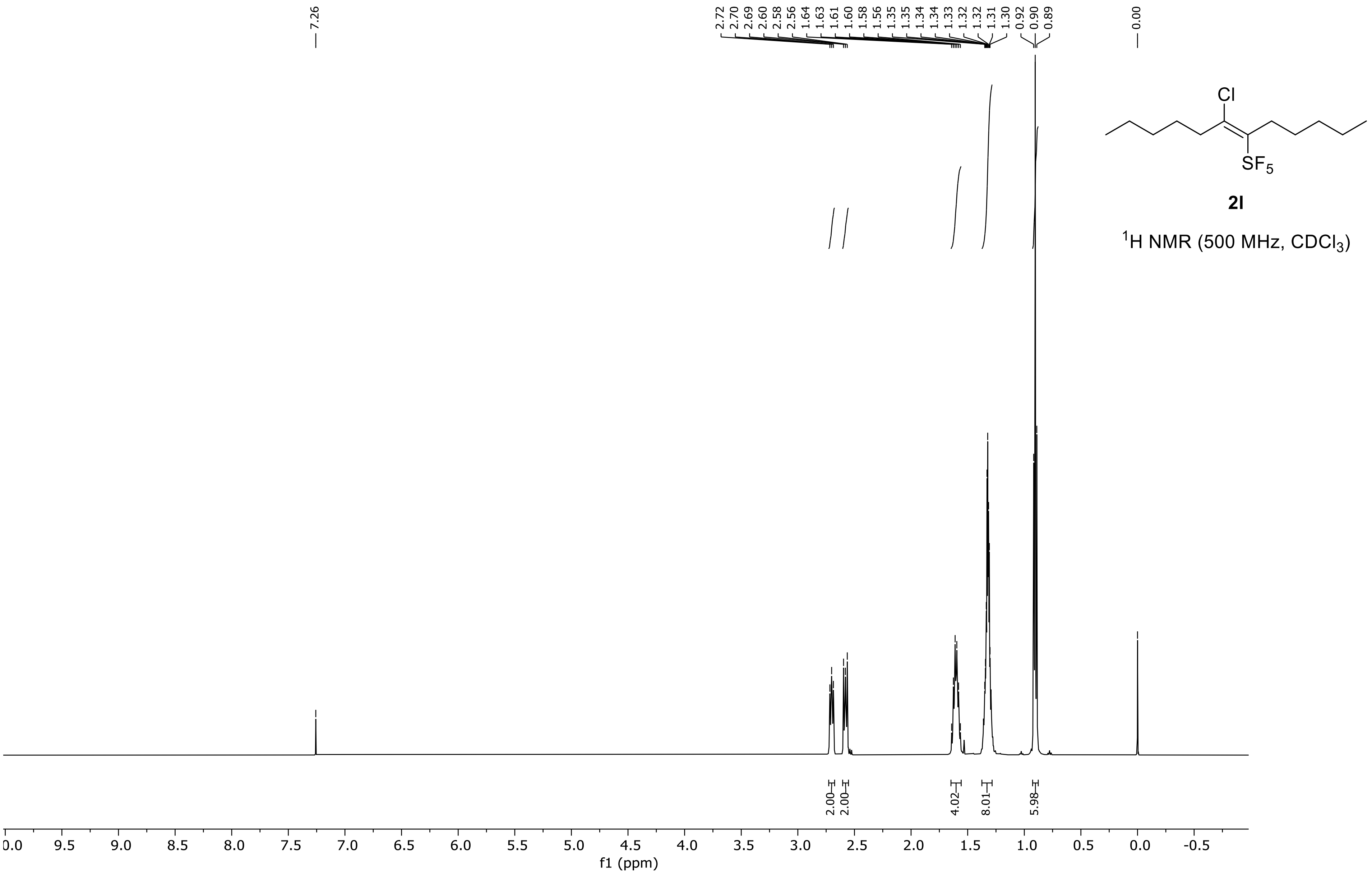
7.26
2.72 2.70 2.69 2.60 2.58 2.56 1.64 1.63 1.61 1.60 1.58 1.56 1.35 1.35 1.34 1.34 1.33 1.32 1.32 1.31 1.30 0.92 0.90 0.89
0.00
Cl
SF5
2l
1H NMR (500 MHz, CDCl3)
2.00 2.00 4.02 8.01 5.98
f1 (ppm)

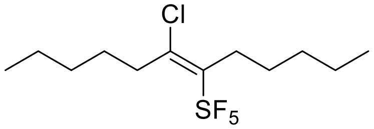

2l

^{13}C NMR (126 MHz, $CDCl_3$)

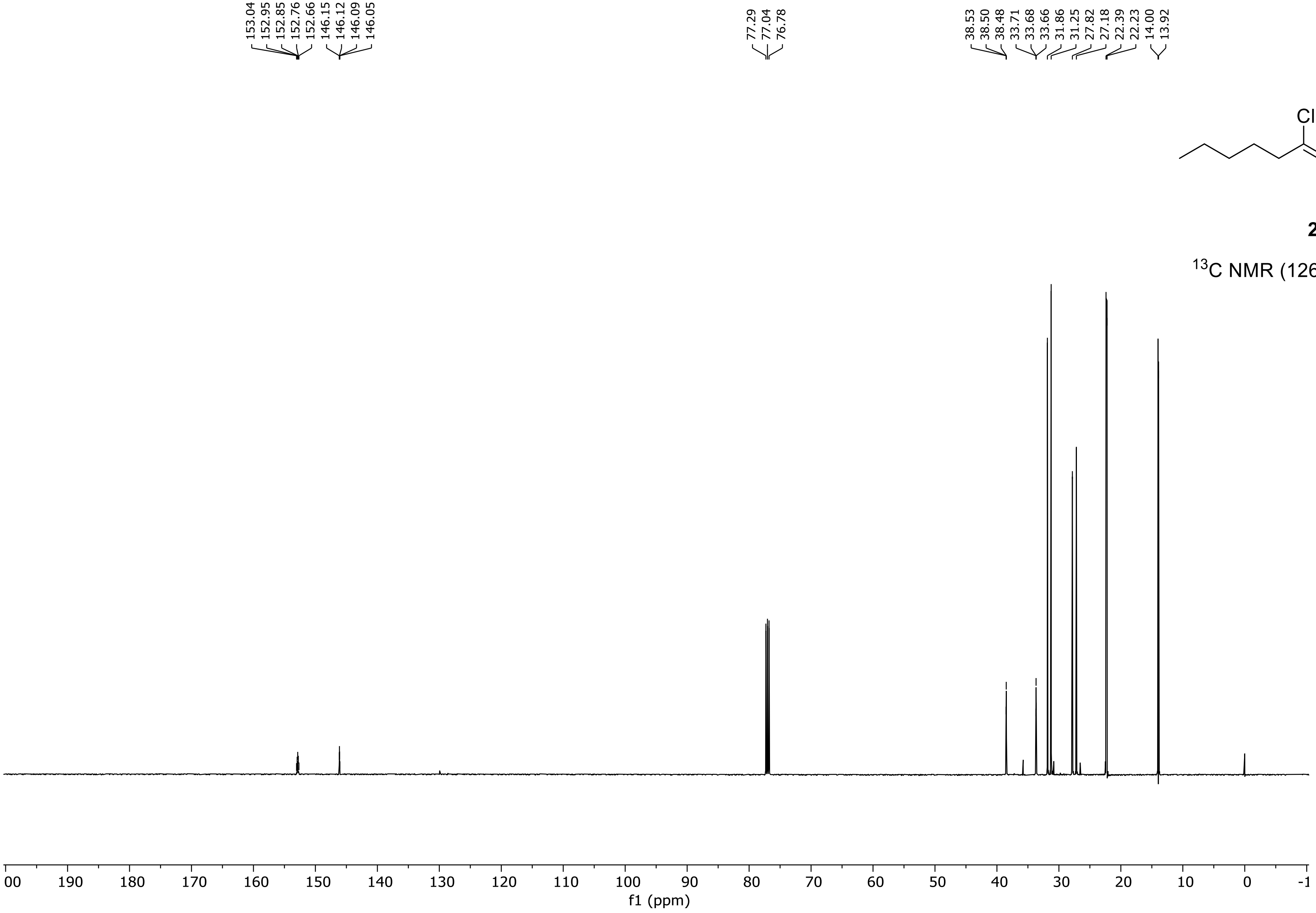

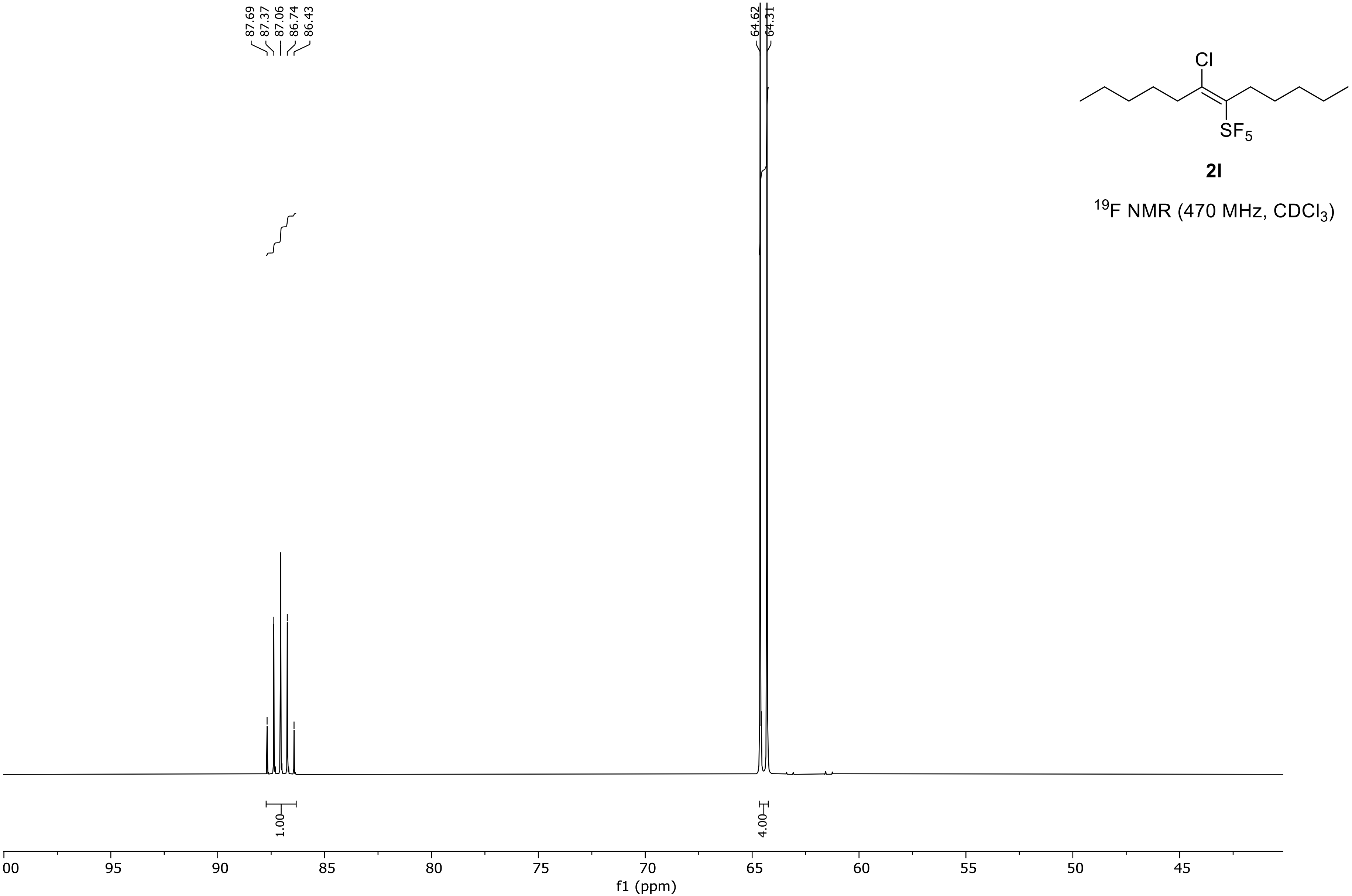

2l

^{19}F NMR (470 MHz, $CDCl_3$)
